# Transcriptome-wide characterization of the eIF4A signature highlights plasticity in translation regulation

**DOI:** 10.1186/s13059-014-0476-1

**Published:** 2014-10-02

**Authors:** Claudia A Rubio, Benjamin Weisburd, Matthew Holderfield, Carolina Arias, Eric Fang, Joseph L DeRisi, Abdallah Fanidi

**Affiliations:** Novartis Institutes for Biomedical Research, Emeryville, CA , 94608-2916 USA; University of California, San Francisco (UCSF), San Francisco, CA 94158 USA; Current address: Leidos Biomedical Research, Frederick National Laboratory for Cancer Research, Frederick, MD 21702 USA

## Abstract

**Background:**

Protein synthesis is tightly regulated and alterations to translation are characteristic of many cancers. Translation regulation is largely exerted at initiation through the eukaryotic translation initiation factor 4 F (eIF4F). eIF4F is pivotal for oncogenic signaling as it integrates mitogenic signals to amplify production of pro-growth and pro-survival factors. Convergence of these signals on eIF4F positions this factor as a gatekeeper of malignant fate. While the oncogenic properties of eIF4F have been characterized, genome-wide evaluation of eIF4F translational output is incomplete yet critical for developing novel translation-targeted therapies.

**Results:**

To understand the impact of eIF4F on malignancy, we utilized a genome-wide ribosome profiling approach to identify eIF4F-driven mRNAs in MDA-MB-231 breast cancer cells. Using Silvestrol, a selective eIF4A inhibitor, we identify 284 genes that rely on eIF4A for efficient translation. Our screen confirmed several known eIF4F-dependent genes and identified many unrecognized targets of translation regulation. We show that 5′UTR complexity determines Silvestrol-sensitivity and altering 5′UTR structure modifies translational output. We highlight physiological implications of eIF4A inhibition, providing mechanistic insight into eIF4F pro-oncogenic activity.

**Conclusions:**

Here we describe the transcriptome-wide consequence of eIF4A inhibition in malignant cells, define mRNA features that confer eIF4A dependence, and provide genetic support for Silvestrol’s anti-oncogenic properties. Importantly, our results show that eIF4A inhibition alters translation of an mRNA subset distinct from those affected by mTOR-mediated eIF4E inhibition. These results have significant implications for therapeutically targeting translation and underscore a dynamic role for eIF4F in remodeling the proteome toward malignancy.

**Electronic supplementary material:**

The online version of this article (doi:10.1186/s13059-014-0476-1) contains supplementary material, which is available to authorized users.

## Background

Energetically, protein synthesis is the most costly step on the path toward gene expression and is thus a rigidly controlled process. In eukaryotes, protein synthesis occurs in three phases: translation initiation, elongation and termination. Although translation is controlled at multiple stages, regulation is primarily exerted at initiation, the phase in which 80S ribosomes assemble onto mRNA transcripts. Regulation of initiation is mediated by multiple factors, many of which converge on the assembly of the eukaryotic initiation factor 4F (eIF4F). This heterotrimeric complex is composed of eIF4E, the rate-limiting protein which binds the 5′-7-methylguanosine cap on cellular mRNA transcripts; eIF4A, a DEAD-box RNA helicase; and eIF4G, a scaffolding protein which bridges eIF4E and eIF4A, and recruits eIF3 and the 43S pre-initiation complex. Formation of eIF4F is tightly controlled by multiple mitogenic signaling pathways, namely mitogen-activated protein kinase (MAPK) and phosphoinositide-3-kinase (PI3K)/Akt/mammalian target of rapamycin (mTOR), and has been shown to stimulate translation of mRNAs involved in cell proliferation, growth, survival, cell cycle progression, and DNA damage repair [1–3]. Moreover, components of the translation apparatus and the rate of protein synthesis are commonly increased in cancer [4,5], overexpression of translation initiation factors, in particular eIF4E and eIF4G, is transforming [6,7], and increased levels of PDCD4, a negative regulator of eIF4A, suppresses transformation [8,9]. Thus, eIF4F has the potential to impact malignant progression yet the mechanism by which increased eIF4F activity can cause transformation remains unclear. Likewise, the particular mechanisms by which different components of eIF4F induce malignancy are not well understood. Nevertheless, eIF4F is a point of convergence for parallel signaling pathways and the complex plays a pivotal role in cancer by integrating aberrant oncogenic signals and amplifying a translational output that can steer the cell toward malignancy.

Significant progress has been made toward understanding the machinery that drives protein synthesis. However, the underlying mechanisms by which individual eIF4F components contribute to translation regulation in the cell remain ambiguous. Emerging methods that allow for global dissection of translation have bolstered the long standing knowledge that translation is subject to considerable regulation and thus plays a key role in regulating gene expression [10–13]. Studies suggest that translation machinery may discriminate between particular mRNA transcripts [14–16] yet the features that might impart individual transcripts with a competitive advantage for eIF4F have not been clearly elucidated. One feature that likely influences the efficiency of translation is the secondary structure of 5′ UTRs. Indeed, engineered 5′ UTR secondary structures have been shown to negatively impact translation efficiency using synthetic reporter constructs [14,17]. Furthermore, eIF4F is required for unwinding 5′ UTR structure on certain mRNAs [18,19] and the degree of structure is proportional to the requirement for eIF4A RNA helicase activity *in vitro* [20]. These data commonly lead to the hypothesis that cellular mRNAs with complex 5′ UTRs must depend more heavily on the eIF4F complex for efficient translation.

eIF4A is an integral part of the heterotrimeric eIF4F complex and the only component with known enzymatic activity. While several eIF4A-regulated genes have been identified, in-depth studies have yet to provide a genome-wide description of the eIF4A target gene landscape. We sought to comprehensively define the cellular mRNAs regulated by eIF4A and investigate the mRNA features that confer dependence on this helicase by directly blocking its activity. To achieve this, we employed the potent and specific eIF4A inhibitor Silvestrol. This compound has been shown to selectively target the RNA helicase activity of eIF4A both *in vitro* and *in vivo*, thereby impairing translation initiation [21,22], and is known to have robust anti-cancer effects [23,24]. Moreover, Silvestrol preferentially inhibits translation of several weakly initiating mRNAs and its anti-oncogenic effects have been proposed to act through favored inhibition of malignancy-related mRNAs [23]. Here we use Silvestrol as a tool to dissect the translational output of eIF4A, define the features that bestow transcripts with a competitive advantage for eIF4A, and elucidate the mechanisms underlying the pro-oncogenic activity of eIF4F.

## Results

**Figure 1:**
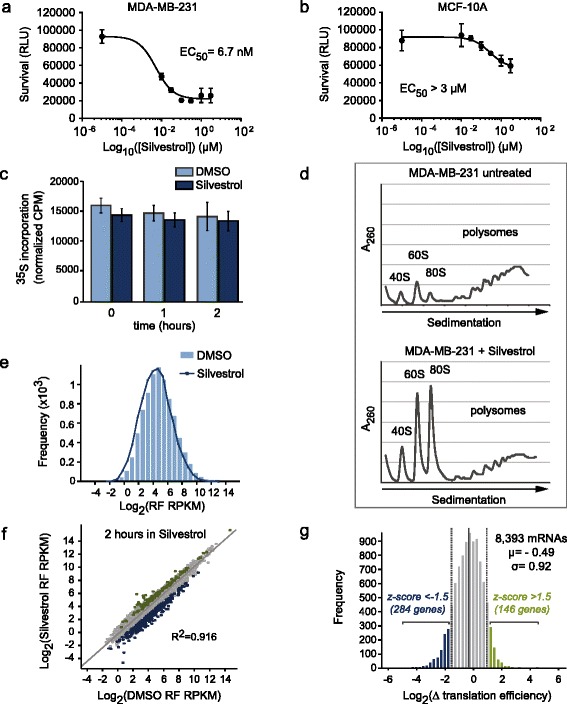
**eIF4A inhibition reveals genes under translational control in MDA-MB-231 cells. (a)** MDA-MB-231 breast cancer cells or **(b)** MCF-10A non-malignant luminal breast cells were treated with increasing concentrations of Silvestrol for 72 hours. Cell proliferation was measured by lysing in Cell Titer Glow reagent and measuring luminescence in relative light units (RLU EC_50_ MDA-MB-231 = 6 nM; EC_50_ MCF-10A >3 μM. **(c)** MDA-MB-231 cells were treated with DMSO or 25 nM Silvestrol, pulsed with ^35^S-methionine for 15 minutes prior to harvest and harvested at 0, 1 or 2 hours after treatment. Bar graph represents counts per minute (CPM) normalized to total protein. Error bars represent standard error (n = 3). **(d)** Polysome profiles of MDA-MB-231 cells treated with DMSO (top panel; 21% ribosomal subunits and 80S monosomes, 79% polysomes; n =2) or with 25 nM Silvestrol (bottom panel; 75% ribosomal subunits and 80S monosomes, 25% polysomes; n =2) for 2 hours. A_260_, the absorbance of light at 260 nm. **(e)** Distribution of ribosome footprint (RF) RPKM values from DMSO-treated MDA-MB-231 cells (mean Log_2_ RPKM value =4.78) compared with Silvestrol-treated cells (mean Log_2_ RPKM value =4.49). RPKM = reads per kilobase per million. **(f)** Scatter plot of RF densities (measured in RPKM) in cells treated with 25 nM Silvestrol versus DMSO for 1 or 2 hours. Silvestrol-sensitive genes are indicated in dark blue. Correlation coefficients for biological replicates (n =2) are DMSO r =0.945 and Silvestrol r =0.977. **(g)** Distribution of changes in translation efficiency (TE) between DMSO- or Silvestrol-treated cells. To calculate TE, RPKM values from RF RNAs were normalized to RPKM values from mRNA sequencing results generated from identical biological samples. Silvestrol-sensitive genes with decreased TE (z-score below -1.5) and Silvestrol-resistant genes (z-score >1.5) are indicated. Population mean indicated by a solid vertical line; dotted vertical lines indicate σ values above and below the mean.

As a model, we selected the triple-negative breast cancer cell line MDA-MB-231 for its known sensitivity to Silvestrol [23]. This allowed for careful optimization of the concentration and duration of compound treatment and provided a context in which to evaluate the contribution of eIF4A-dependent translation to malignancy. Exposure of MDA-MB-231 cells to increasing concentrations of Silvestrol impaired cell survival (Figure 1a) while the compound had minimal effects on survival of non-malignant MCF-10A cells (Figure 1b). Likewise, the effect of Silvestrol on global translation was measured by monitoring ^35^S-methionine incorporation into newly synthesized proteins. To mitigate off-target or secondary effects of drug treatment, we chose to treat cells with a low concentration of Silvestrol for a short time period. We selected a concentration of 25 nM Silvestrol, which had negligible effects on global translation after 2 hours of exposure (Figure 1c). To further evaluate the effect of eIF4A inhibition on the translation apparatus, we analyzed the polysome profiles of MDA-MB-231 cells left untreated or treated with 25 nM Silvestrol for 2 hours. Silvestrol treatment liberated 40S and 60S ribosomal subunits, dramatically increased the abundance of 80S monosomes and had minimal effects on the abundance of polysomes (Figure 1d). In contrast, treatment with harringtonine, which globally arrests ribosomes at initiation codons, caused an accumulation of ribosomal subunits and 80S monosomes and eliminated polysomes (Additional file 1). Taken together, these results suggested that Silvestrol blocked the translation of a subset of mRNA transcripts at initiation. To explore the possibility that a select mRNA subset is regulated by eIF4A, we employed the comprehensive, transcriptome-wide method of ribosome profiling [10–13,15,16]. This approach allows for the detailed analysis of transcriptome utilization by translation machinery through the use of next-generation sequencing to quantify ribosome-protected mRNA fragments (ribosome footprints (RFs)). MDA-MB-231 cells were treated with vehicle (DMSO) or 25 nM Silvestrol and cell lysates, collected at 1 and 2 hours after treatment, were RNase-digested and fractionated to collect monosomes. Mass spectrometry analysis of monosome fractions used for ribosome profiling revealed that the majority of peptides present were derived from ribosomal components in both DMSO- and Silvestrol-treated lysates, indicating that protected mRNAs isolated by our method were engaged by ribosomes (Table S1 in Additional file 2). Likewise, the average length of sequenced fragments in all samples was approximately 32 nucleotides, the expected size of a ribosome-protected mRNA fragment (Figure S2a in Additional file 3). We identified an average of 43 million reads that mapped to a total of 17,703 annotated mRNA transcripts (Figure S2b in Additional file 3). Of these, 8,393 genes were sufficiently represented in our sequencing data to provide reliable measurements of their translational status. Sequencing data were quantified by calculating the reads per kilobase per million (RPKM) value for each gene. The RPKM values generated from sequencing ribosome-protected fragments directly reflect the extent to which a given transcript is engaged by ribosomes [10]. Comparison of RF data from DMSO- and Silvestrol-treated cells revealed that genome-wide translation was not significantly reduced after exposure to compound for 2 hours (Figure 1e). To identify genes that depend on eIF4A for robust translation, the translation efficiency (TE) value of each transcript was calculated. TE values are generated by dividing RPKM values from RF data by RPKM values from mRNA-seq data, thereby normalizing translation to steady-state transcript abundance. The TE value thereby provides a quantitative measure of transcript-specific utilization by translation machinery and uncouples changes in transcription from changes due to translation regulation. We calculated the fold change in TE (∆TE) due to eIF4A inhibition by dividing TE values from Silvestrol-treated cells by those from DMSO-treated cells; thus, ∆TE = TE_Silvestrol_/TE_DMSO_ for each gene. Of the 8,393 genes whose translation was robustly measured, we identified 284 genes that showed markedly reduced translation as a result of eIF4A inhibition (z-score below -1.5; Table S2 in Additional file 2) and 146 genes that showed increased translation upon eIF4A inhibition (z-score above 1.5; Table S3 in Additional file 2) (Figure 1f-g; Figure S2c in Additional file 3).

To validate the observation that Silvestrol reduces translation of these genes, we monitored the polysome association of specific mRNAs. Polysomes from MDA-MB-231 cells treated with 25 nM Silvestrol or vehicle were fractionated by sucrose gradient and the resulting fractions were analyzed by quantitative RT-PCR for *CyclinD1*, *ARF6*, *BCL2*, *ROCK1*, *CDK6*, and *β-actin* Consistent with our ribosome profiling data, significant shifts in polysomes were observed for *CyclinD1*, *ARF6*, *BCL2*, *ROCK1*, and *CDK6*, but not for *β-actin* (Additional file 4). These data confirm that TE values obtained by ribosome profiling are consistent with changes in ribosome occupation on specific transcripts.

**Figure 2:**
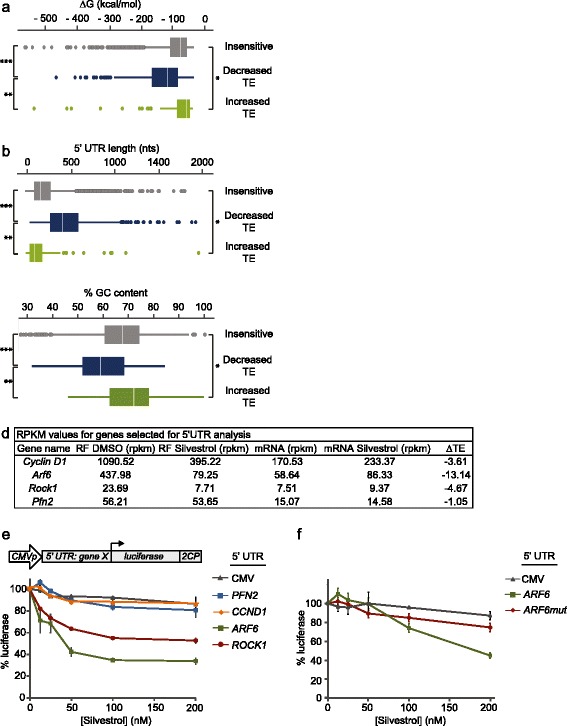
**Silvestrol-sensitive mRNAs are enriched with complex 5′ UTRs. (a)** Box plot showing distribution of free energy values for 5′ UTRs of Silvestrol-sensitive genes (Decreased, n =284, z-score below -1.5), genes increased in Silvestrol (n =146 genes, z-score >1.5), and genes insensitive to Silvestrol (n =6,303). ***P =2.53 × 10^-67^, **P =1.097 × 10^-27^ and *P =4.25 × 10^-7^. Energy values were predicted using the CONTRAfold algorithm [25]. **(b)** Box plot showing distribution of 5′ UTR length, L: ***P =1.39 × 10^-133^, **P =3.1 × 10^-44^ and *P =4.83 × 10^-10^. **(c)** Box plot showing distribution of GC content: ***P =1.02 × 10^-47^, **P =3.35 × 10^-23^ and *P =8.08 × 10^-6^. For genes with decreased TE: n =284, ∆G_mean_ = -103.9 kcal/mol, L_mean_ =493.4 nucleotides, GC_mean_ =59.8%. For insensitive genes: n =6,303, ∆G_mean_ = -54.72 kcal/mol, L_mean_ =223.6 nucleotides, GC_mean_ =66.9%. For genes with increased TE: n =142, ∆G_mean_ = -45.93 kcal/mol, L_mean_ =171.8 nucleotides, GC_mean_ =71.23%. Significance values were determined using the two-tailed Mann-Whitney U test **(d)** RPKM and TE values from ribosome profiling data for genes used in 5′ UTR analyses of *CyclinD1*, *ARF6*, *ROCK1* and *PFN2*. **(e)** Luciferase reporter constructs, stably transfected into 293 T cells, were treated with increasing concentrations of Silvestrol and luciferase expression was measured after 40 minutes; constructs bearing 5′ UTRs from Silvestrol-sensitive genes (*CyclinD1*, *ROCK1*, *ARF6*) or insensitive genes (*PFN2* or *CMV* alone) were compared. Triplicate values were obtained in each experiment; data presented were obtained from four independent experiments. **(f)** Luciferase reporter expression in stably transfected 293 T cells treated with increasing concentrations of Silvestrol; constructs bearing *ARF6wt* 5′ UTR or *ARF6mut* 5′ UTR were compared; data presented were obtained from two independent experiments with measurements taken in triplicate.

The translation initiation complex eIF4F has been shown to regulate genes with exogenous structural elements added to their 5′ UTRs [14,17–19]. Since Silvestrol inhibits the RNA helicase component of eIF4F, we surmised that the 5′ ends of Silvestrol-sensitive genes would be more structured than genes insensitive to Silvestrol. Using CONTRAfold, a statistical learning structure prediction algorithm [25], we evaluated the secondary structure of 5′ UTRs derived from the 284 mRNAs that showed reduced translation upon Silvestrol treatment. To control for this analysis, we analyzed 5′ UTRs from Silvestrol-insensitive genes (n =6303) in parallel with those from the 284 Silvestrol-sensitive genes. We found that the pool of 5′ UTRs from Silvestrol-sensitive genes was enriched with structured elements when compared to the control pool. 5′ UTRs from mRNAs with z-scores below -1.5 had more negative free energy values than those with z-scores above -1.5 (Figure 2a; Figure S4a in Additional file 5). The difference in free energy values between genes with decreased TE in Silvestrol and insensitive genes was highly significant (*P* =2.53 × 10^-67^) and a difference in distribution of ∆G values from genes with increased TE was also notable compared with the insensitive pool (*P* =4.25 × 10^-7^) (Figure 2a). Evaluation of 5′ UTRs for length and percentage GC content revealed that mRNAs with decreased TE had markedly longer 5′ UTRs than those from insensitive genes (*P* =1.39 × 10^-133^) while those from genes with increased TE were comparatively shorter (*P* =4.82 × 10^-10^; Figure 2b). Likewise, the overall percentage GC content was significantly lower in the 5′ UTRs of mRNAs with decreased TE (*P* =1.02 × 10^-47^) and slightly higher in those from genes with increased TE (*P* =8.08 × 10^-6^) (Figure 2c). These data suggest that eIF4A-dependent genes have a 5′ UTR signature characterized by long, structured sequences with relatively low overall GC content.

To evaluate the contribution of gene-specific 5′ UTRs to eIF4A-dependence we created reporter constructs bearing select 5′ UTRs derived from our analyses. We made use of the short-lived luciferase reporter, *luc2CP*, which contains two protein-destabilizing sequences, hCL1 and hPEST, that reduce the half-life of luciferase to approximately 30 minutes (Figure S4b in Additional file 5). This allowed us to uncouple the contribution of protein stability to our measurement and monitor Silvestrol-sensitive changes in translation on a short time scale. We placed the *luc2CP* gene under control of the CMV promoter and fused the *luc2CP* open reading frame to the 5′ UTR sequences from three Silvestrol-sensitive genes, *CyclinD1*, *ARF6* and *ROCK1*, and one Silvestrol-insensitive gene, *PFN2* (Figure 2e; FigureS4c-e in Additional file 5). These constructs were stably expressed in 293T cells and evaluated for sensitivity to Silvestrol by measuring luciferase activity in the presence of increasing amounts of Silvestrol after 40 minutes of exposure to compound. Reporter constructs bearing highly structured 5′ UTRs, from *ARF6* (∆G = -264.5 kcal/mol) and *ROCK1* (∆G = -313 kcal/mol), were significantly more sensitive to Silvestrol than unstructured controls, CMV alone and *PFN2* (∆G = -115.57 kcal/mol) (Figure 2f; Table S4 in Additional file 2). These differences were directly attributable to changes in translation since mRNA levels were unaffected by the presence of Silvestrol (Figure S4f in Additional file 5). In addition, treatment with hippuristanol, a second inhibitor of eIF4A, produced similar results in our reporter assay (Additional file 6). Notably, the relatively unstructured 5′ UTR of *CyclinD1* (∆G = -47 kcal/mol) (Figure S6a,b in Additional file 7) had little impact on the sensitivity of our reporter to eIF4A inhibition.

To further assess the role of 5′ UTR complexity in the Silvestrol-sensitivity of our reporter, we sought to disrupt structured regions within the 5′ UTR of *ARF6*. To this end, we predicted regions of structure by determining the ∆G value of 20-nucleotide fragments across the length of the *ARF6* 5′ UTR. The resulting ∆G values were plotted across the 5′ UTR to reveal short regions of complex structure (Figure S6c in Additional file 7). Evaluation of the percentage GC content across the length of the 5′ UTR revealed that localized structures in the 5′ UTR correlated well with clusters of high GC content (Figure S6d in Additional file 7). Based on these predictive analyses, a series of single nucleotide changes were made to the *ARF6* 5′ UTR to remove base-pairing interactions in structured regions. Additionally, a long-range base-pairing interaction expected to obscure the *ARF6* translation start codon was disrupted by mutagenesis. The resulting 5′ UTR, termed *ARF6mut*, was identical in length and overall GC content but its localized structures and GC-rich peaks were removed or reduced (Figure S6e-f in Additional file 7; see the mutated residues in the 'Oligonucleotide' section in Materials and methods). The *ARF6mut* 5′ UTR had an increased ∆G value of -142.4 kcal/mol (compared with the wild-type ∆G_*ARF6* 5′ UTR_ of -264.5 kcal/mol). The introduction of *ARF6mut* into our *luc2CP* reporter decreased the sensitivity of our reporter construct to Silvestrol (Figure 2f), indicating that reducing structure in the 5′ UTR of *ARF6* decreased eIF4A-dependency of the reporter. Taken together, these data demonstrate that structured regions in the 5′ UTR of Silvestrol-sensitive *ARF6* mRNA confer dependence on eIF4A for translation initiation and that the degree of structure correlates with increased sensitivity to eIF4A inhibition.

**Figure 3:**
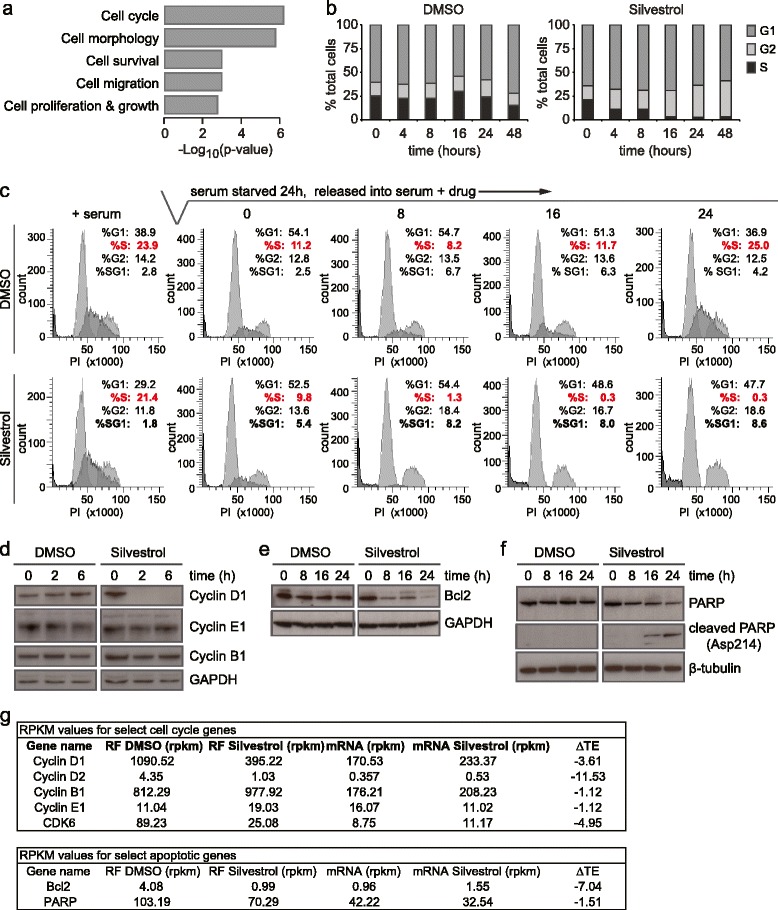
**eIF4A regulates cell cycle progression and apoptosis. (a)** Gene ontology (GO) analysis predictions of the effects of Silvestrol on cancer-related pathways in MDA-MB-231 cells. **(b)** Cell cycle progression of MDA-MB-231 cells was monitored after treatment with DMSO (left) or Silvestrol (right) for 24 hours. Cells were pulsed with bromodeoxyuridine (BrDU) for 30 minutes prior to harvest. Upon harvest, cells were fixed, stained with anti-BrDU-fluorescein isothiocyanate (FITC) antibody and propidium iodide (PI) and analyzed by fluorescence-activated cell sorting (FACS) **(c)** MDA-MB-231 cells were synchronized by serum starvation for 24 hours then released from starvation in the presence of DMSO (top panels) or Silvestrol (bottom panels). The cell cycle was monitored over time by BrDU incorporation and PI staining as in Figure 3a. **(d)** MDA-MB-231 cells were treated with DMSO or Silvestrol for 0, 2 or 6 hours prior to lysis. Lysates were analyzed by Western blotting for proteins indicated. **(e,f)** MDA-MB-231 cells were treated with DMSO or Silvestrol from 0, 8, 16 and 24 hours prior to lysis; lysates were analyzed by Western blotting for indicated proteins. **(g)** Tables showing RPKM values from ribosome profiling data for genes implicated in cell cycle progression (top) and apoptosis (bottom).

Gene ontology (GO) analysis of Silvestrol-sensitive genes revealed that a large proportion of eIF4A-dependent genes were involved in cell cycle progression (*P* =7.37 × 10^-7^; Figure 3a). Since the cell cycle is often misregulated in cancer, we explored the biological consequence of Silvestrol treatment by examining the impact of eIF4A inhibition on cell cycle progression. Asynchronous MDA-MB-231 cells were treated with Silvestrol and the cell cycle was monitored by bromodeoxyuridine (BrDU) incorporation and propidium iodide (PI) staining followed by flow cytometry. Silvestrol treatment prevented MDA-MB-231 cells from entering S phase and caused accumulation in the G2 phase of the cell cycle (Figure 3b). To further explore the block to S phase transition, we investigated the effect of Silvestrol in synchronized cells. MDA-MB-231 cells were arrested in early G1 by serum starvation, then released from starvation by the addition of serum-containing medium in the presence or absence of Silvestrol and cell cycle progression was monitored over time. Upon release from serum starvation, cells accumulated in G1 phase, which coincided with a robust block to S phase entry in the presence of Silvestrol. This block was accompanied by a significant increase in sub-G1 cells, indicating the onset of cell death during prolonged drug treatment (Figure 3c, bottom panels). Indeed, GO analysis of our ribosome profiling data predicted that Silvestrol treatment would impair cell survival (*P* =1.12 × 10^-3^; Figure 3a). In this assay, we also observed that cells remaining in S phase after serum starvation were able to enter G2, indicated by a rise in the percentage of G2 cells 24 hours after release in the presence of Silvestrol (Figure 3c, bottom panels). Thus, Silvestrol prevented cells from entering S phase but not G2.

Based on our ribosome profiling data, we identified eIF4A-regulated genes that act at the G1/S phase transition. In particular, the translation but not transcription of Cyclin D1, Cyclin D2 and CDK6 was sensitive to Silvestrol treatment (Figure 3g). Upon Silvestrol treatment, we observed a rapid loss of Cyclin D1 protein but no loss of Cyclin E1 or Cyclin B1 protein (Figure 3d). Likewise, we observed a significant, Silvestrol-mediated loss of the anti-apoptotic factor Bcl2 (Figure 3e), attributable to a block in *BCL2* translation rather than transcription (Figure 3g). Loss of Bcl2 protein was accompanied by cleavage of PARP (Figure 3f) and occurred concomitantly with the observed increase in sub-G1 cells (Figure 3c). Together, these data suggest that inhibition of eIF4A in MDA-MB-231 cells primarily blocks cell cycle progression at the G1/S phase transition, likely through loss of Cyclin D1, Cyclin D2 and Cdk6, and induces apoptosis, in part, by inhibiting translation of *BCL2*.

Besides targeting genes important for cell cycle progression and survival, our data indicated that Silvestrol had a significant effect on genes involved in cell migration (*P* =1.13 × 10^-3^; Figure 3a). Indeed, inhibition of eIF4F was recently shown to impair migration of highly metastatic cancers [26]. To explore this further, we measured the migration of MDA-MB-231 cells in a trans-well migration assay. In this assay, cell migration was inhibited at low concentrations of Silvestrol (Figure S7a in Additional file 8) and decreased in a dose-dependent manner. Furthermore, a scratch wound assay showed a block to wound closure in the presence of Silvestrol (Figure S7b,c in Additional file 8). Among the genes we identified as Silvestrol-sensitive, *ARF6* is a master regulator of cell migration in MDA-MB-231 cells [27,28]. In our study, inhibition of cell migration was accompanied by a loss of Arf6 protein in a time-dependent manner similar to the impairment of migration (Figure S7d in Additional file 8).

**Figure 4:**
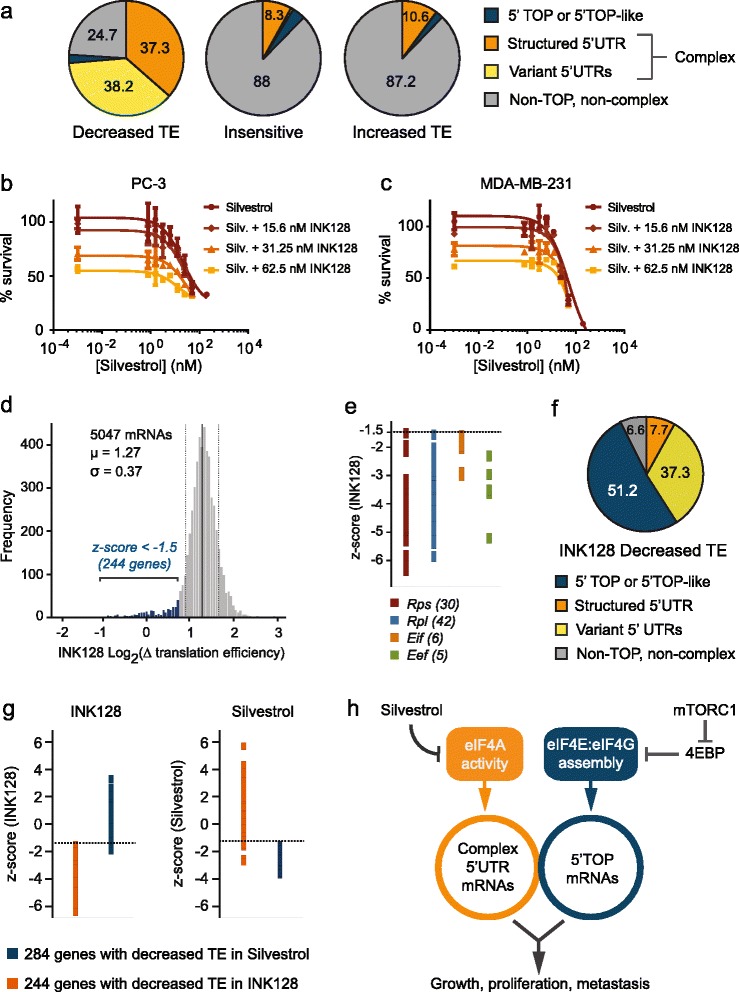
**eIF4A regulates the TE of a discrete mRNA subset with common features. (a)** 5′ UTR sequences from Silvestrol-responsive genes were analyzed for secondary structure, variant 5′ UTRs and 5′ TOP sequences. Complex 5′ UTRs are defined as those with significant secondary structure (∆G < -104 kcal/mol) or annotated 5′ UTR variants. Genes with decreased TE: 37.3% structured, 38.2% variant, 2.3% 5′ TOP or 5′ TOP-like, 24.7% non-TOP and non-complex. Insensitive TE genes: 8.3% structured, 0.3% variant, 3.7% 5′ TOP or 5′ TOP-like, 88% non-TOP and non-complex. Genes with increased TE: 10.6% structured, 0% variant, 2.1% 5′ TOP or 5′ TOP-like, 87.2% non-TOP and non-complex. **(b,c)** PC-3 (b) or MDA-MB-231 (c) cells were treated with increasing concentrations of Silvestrol in combination with INK128 at fixed doses: 15.6 nM, 31.25 nM and 62.5 nM. Cell viability was measured by CellTiter-Glo after 3 days. **(d)** Distribution of changes in TE upon INK128 treatment of MDA-MB-231 cells. INK128-sensitive genes with decreased TE (z-score < -1.5) are indicated. Population mean indicated by a solid vertical line; dotted vertical lines indicate σ values above and below the mean. **(e)** z-scores for INK128-dependent changes in TE for known 5′ TOP mRNAs. The dotted line is drawn at z-score = -1.5. **(f)** 5′ UTR characteristics for genes with INK128-dependent reduction in TE; 51.2% 5′ TOP or 5′ TOP-like, 37.3% variant, 7.7% structured and 6.6% non-TOP and non-complex. **(g)** z-scores of transcripts in INK128-treated (left panel) or Silvestrol-treated (right panel) MDA-MB-231 cells. Each point represents a single gene; genes in orange had decreased TE in INK128 while those in blue had decreased TE in Silvestrol. Dotted lines are drawn at z-score = -1.5. **(h)** A model proposing that the subset of mRNAs most sensitive to eIF4A inhibition is distinct from the pool of mRNAs regulated via mTOR-mediated assembly of eIF4E and eIF4G.

Recently, an in-depth investigation into the genome-wide program of mTOR-regulated genes identified a subset of growth-related mRNAs regulated by eIF4E via the 4E-binding proteins 4E-BP1/2. This pool of mRNAs was overwhelmingly characterized by the presence of 5′ TOP and 5′ TOP-like sequences adjacent to the 5′-m^7^GTP cap and lacked mRNAs with long, structured 5′ UTRs [15,16]. Unexpectedly, we found that the population of eIF4A-dependent mRNAs did not carry 5′ UTRs bearing 5′ TOP sequences, but was rather characterized by long, complex 5′ UTRs containing significant structure and an increased prevalence of 5′ UTR sequences with alternative transcription start sites (TSSs) or 5′ UTR introns. We collectively term these ‘variant 5′ UTRs’ (Figure 4a). Taken together, these data suggest that diminishing eIF4F assembly through 4E-BP1/2 is not equivalent to direct abrogation of eIF4A helicase activity. These intriguing observations imply that the subset of eIF4A-dependent mRNAs is distinct from those which rely on efficient eIF4F assembly for translation. To explore this idea, we selected two cell lines, MDA-MB-231 and PC-3 prostate cancer cells, for their known sensitivity to inhibition of either eIF4A or mTOR. We first treated MDA-MB-231 or PC-3 cells with DMSO, Silvestrol or INK128, a catalytic mTOR inhibitor, and analyzed lysates by Western blot for two eIF4A-sensitive proteins, Cyclin D1 and Arf6. We found that neither Arf6 nor Cyclin D1 protein production was affected by INK128 treatment of MDA-MB-231 (Figure S8a in Additional file 9) or PC-3 cells (Figure S8b in Additional file 9). This clue that eIF4A and mTOR/4E-BPs differentially affect translation lead us to hypothesize that catalytic inhibition of mTOR in combination with Silvestrol would have additive rather than synergistic or epistatic activity compared with Silvestrol alone. To test this, cells were treated with Silvestrol in combination with one of two catalytic mTOR inhibitors, PP242 or INK128 [15], and survival was measured 72 hours after exposure. The inclusion of INK128 had an additive effect on cell survival compared with treatment with Silvestrol alone in both cell lines (Figure 4b,c; Figure S9a,b in Additional file 10). Likewise, treatment with PP242 was additive in combination with Silvestrol as further inhibition of survival in both cell types was observed even at very low concentrations of compound (Figure S9c-f in Additional file 10). Dose-matrix plots of these data (lower panels of Figure S9a,b,e,f in Additional file 10) are best described by a highest single agent- or Bliss-like masking model in which dual inhibition of targets across two intersecting pathways is required for full inhibition [29].

To evaluate whether mTOR inhibition specifically reduces the translation of a different mRNA subset from that regulated by eIF4A, we performed ribosome profiling of MDA-MB-231 cells treated with INK128. We identified 244 transcripts that showed reduced TE in the presence of INK128 (Figure 4d). This pool of mTOR-dependent genes shared only one member in common with those showing decreased TE in the presence of Silvestrol and was identical to the pool of genes identified as mTOR-dependent by Hsieh *et al*. [15] and Thoreen *et al*. [16] (Table S6 in Additional file 2). Moreover, INK128-sensitive transcripts were predominantly composed of known 5′ TOP mRNAs (Figure 4e) as well as transcripts bearing 5′ TOP-like sequences [16,30] and largely unstructured 5′ UTRs (Figure 4f). Notably, we found that variant 5′ UTRs occurred in INK128-dependent mRNAs at nearly the same frequency as in those showing decreased TE in Silvestrol (Figure 4f, compared with Figure 4a), though none of these genes were common to both pools (Table S6 in Additional file 2). Indeed, the TE of INK128-sensitive genes was not reduced in Silvestrol-treated cells, nor did Silvestrol-sensitive genes show reduced TE upon INK128 treatment (Figure 4g). Taken together, these data support a model in which the pool of mRNAs that are regulated through the assembly of eIF4E with eIF4F components is discrete from the mRNA subset that depends on eIF4A activity for efficient translation (Figure 4h).

## Discussion

Here we confirm and refine the long-standing hypothesis that the eIF4F heterotrimer selectively regulates the translation of mRNAs with long, structured 5′ UTRs and provide a genome-wide account of genes that rely on eIF4A activity for translation. Previous studies have shown that structured RNA elements derived from exogenous sources increased dependency on eIF4F when added to 5′ UTR reporters [14,17–20]. However, our work provides the first evidence that this principal is upheld in the context of the entire transcriptome and the first demonstration that structured 5′ UTRs derived directly from cellular transcripts confer dependence on eIF4A for efficient translation.

While our data confirm that structured 5′ UTRs require eIF4A activity, this work indicates that the length and structure of the 5′ UTR are not the only features that confer eIF4A dependence. In addition to increased secondary structure and length, we observed that 38.16% of Silvestrol-sensitive genes have the capacity to encode multiple 5′ UTR variants while only 0.3% of insensitive genes carry a variant 5′ UTR (Figure 4a; Table S4 in Additional file 2). These variants are characterized by the presence of alternative TSSs or 5′ UTR introns that are not prevalent in Silvestrol-insensitive genes. This unforeseen observation suggests that modification of the 5′ UTR, resulting from the use of alternative TSSs or alternative splicing events, could convey different modes of translational control on otherwise similar mRNA transcripts. One attractive hypothesis is that alternative splicing of 5′ UTR sequences facilitates translation of spliced mRNAs through recruitment of eIF4A3. In support of this, evidence suggests that the role of eIF4A3 in translation includes the ATP-dependent rearrangement of protein-protein or protein-mRNA complexes since this nuclear isoform of eIF4A binds mRNAs at exon junctions [31–33]. Understanding how and when alternative TSSs or splicing events are used to generate 5′ UTR variants and exploring the mechanisms by which such variants are regulated by the translation machinery are intriguing avenues for future investigation.

The observation that translation of *CyclinD1*, which bears a relatively short, unstructured 5′ UTR, was strongly regulated in an eIF4A-dependent manner was unanticipated. Indeed, eIF4A has been shown to enhance pre-initiation complex scanning even on mRNAs with unstructured 5′ UTRs [19]. Consistent with this, we observed that the less-structured 5′ UTR of *CyclinD1* is unable to impart Silvestrol-sensitivity to our reporter yet the translation of *CyclinD1* itself is sensitive to eIF4A inhibition (Figure 3c). These observations suggest that some determinants of eIF4A dependence, which govern the translation of *CyclinD1* and possibly other transcripts with unstructured 5′ UTRs, have yet to be discovered. Alternatively, the observed decrease in *CyclinD1* translation could be the result of an indirect effect, whereby the translation of a regulatory factor governing *CyclinD1* is itself modulated by Silvestrol.

While this manuscript was under revision, a similar study using Silvestrol in T-cell acute lymphoblastic leukemia was published by Wolfe *et al*. [34]. The results of this study agree with our findings that eIF4A regulates transcripts with long, structured 5′ UTRs and show that an additional 9- or 12-nucleotide guanine quartet, (CGG)_4_, marks eIF4A-dependent transcripts. In our study, approximately 25% of 5′ UTRs from genes with reduced TE in MDA-MB-231 cells contain this repeat. Notably, an intact (CGG)_4_ motif is present in both the *ARF6* and *ARF6mut* 5′ UTR reporter constructs (see Materials and methods), which display differential sensitivities to eIF4A inhibition. Thus, additional 5′ UTR structural features contribute significantly to eIF4A-mediated translation. While the guanine quartet marks a subset of eIF4A-dependent transcripts, our data indicate that the presence of a (CGG)_4_ motif in the 5′ UTR is not a sole determinant of eIF4A dependence.

Finally, we observed that many 5′ UTRs from Silvestrol-insensitive mRNAs have extremely low ∆G values and some of these values overlap with those of Silvestrol-sensitive genes. One possible explanation is that helicases with a redundant function in translation, such as DHX29, might serve to unwind highly structured mRNAs when eIF4A activity is compromised [35].

In addition to describing mRNA features that contribute to eIF4A dependence, transcriptome-wide ribosome profiling of Silvestrol-treated cells provided genetic insight into the physiological consequences of eIF4A inhibition. Silvestrol-related compounds have been suggested to block progression through the cell cycle [36] yet the genetic basis for this block has not been well explored. We found that ribosome occupancy of specific transcripts, such as *CyclinD1*, *CyclinD2*, *CDK6* and *CDK8*, which regulate cell cycle progression, is reduced by Silvestrol treatment (Figure 3g; Table S2 in Additional file 2) and that Cyclin D1 protein levels drop significantly upon eIF4A inhibition. Notably, Cyclin E1 and Cyclin B1 production was unaffected by Silvestrol, demonstrating that loss of Cyclin D1 is specific and cannot be attributed to the short half-life of the protein. Furthermore, Silvestrol-like compounds induced apoptosis through Caspase 9 cleavage in LNCaP cells [36]. We found that Silvestrol caused a significant loss of ribosomes on the *BCL2* transcript and a loss of Bcl2 protein (Table S2 in Additional file 2; Figure 3e,g; ∆TE = -7.04). Depletion of Bcl2 correlated with an observed increase in sub-G1 cells (Figure 3c bottom panels) and cleavage of PARP (Figure 3f), suggesting that eIF4A mediates cell survival, at least in part, by regulating *BCL2* translation. Finally, a previous study found that eIF4F inhibition impaired cell migration in highly metastatic TM15 breast cancer cells [26]. Accordingly, our work indicates that cell migration is regulated in an eIF4A-dependent manner (Figure 3a; Additional file 6). We show that Silvestrol strongly blocks ribosome assembly on *ARF6* (Table S2 in Additional file 2; ∆TE = -13.14), a key regulator of migration, and causes loss of Arf6 protein (Figure S5d in Additional file 6). Indeed, silencing *ARF6* in MDA-MB-231 cells has been shown to impair migration and invasion [27] and *ARF6* overexpression in conjunction with a partner, *Gep100*, triggered invasion in non-invasive MCF-7 cells [28]. Our data indicate that eIF4F mediates cell migration through eIF4A yet, taken jointly with our cell survival results, the impact of eIF4A inhibition on migration cannot be separated from the induction of cell death. Since apoptosis becomes evident in MDA-MB-231 cells 8 hours after exposure to Silvestrol (Figure 3c,f), measurements of cell migration taken beyond 8 hours in compound should be interpreted with caution. Nevertheless, the novel finding that *ARF6* translation relies on eIF4A activity highlights the central role of this helicase in controlling metastatic potential in MDA-MB-231 cells. Our findings illustrate the impact of Silvestrol on cellular processes with pro-oncogenic properties and highlight translation initiation as a principal regulatory node in malignant progression.

Importantly, the helicase activity of eIF4A *in vitro* is known to be strongly stimulated by its association with other initiation factors, namely eIF4G, eIF4B and eIF4H [37,38]. Thus, eIF4A requires interaction with partner proteins for efficient activity. Our findings suggest that abrogating eIF4A activity is not equivalent to interrupting the assembly of eIF4E and eIF4G, since inhibition of both appears to be required to achieve a maximal block to eIF4F function. If disrupting the integrity of eIF4E-eIF4G interaction were equivalent to abolishing eIF4A activity, we would not have expected the effects of Silvestrol and mTOR inhibitors to be additive. Furthermore, the pool of transcripts most dependent on eIF4A for translation is distinct from those impacted by mTOR inhibition, which abrogates eIF4E-eIF4G assembly. This holds true both in the identity of genes which comprise each pool and in the features that define their dependency. These observations may be explained by one or more of the following possibilities. First, different isoforms of the canonical eIF4F complex may exist that have variable activity and/or differential target transcripts. Indeed, eIF4G2, a homolog of eIF4G, significantly impacts protein synthesis [39] but does not bind eIF4E and is not required for translation of 5′ TOP mRNAs [16,40]. Second, stimulation of eIF4A activity could occur through an eIF4E-independent mechanism, thereby diminishing the requirement for eIF4E:eIF4G assembly on certain transcripts. Third, eIF4E and eIF4G may have the capacity to assemble and recruit translation machinery in the absence of eIF4A. This would suggest that eIF4A is only stringently required on transcripts with long, structured 5′ termini and, possibly, 5′ UTR introns. Fourth, association of eIF4A in the eIF4F complex may be sufficient for activation of eIF4F-mediated translation even in the absence of helicase activity. In this scenario, the active helicase would only be required when scanning initiation complexes encounter structured regions in target transcripts. Alternatively, association of eIF4A with certain unstructured or short 5′ UTRs, with or without associated factors, might negatively impact translation. Under these circumstances, blocking eIF4A activity would relieve inhibition and may underlie the observation that a small subset of transcripts exhibit increased TE when exposed to Silvestrol. These intriguing possibilities warrant further dissection of the interactions between initiation factors, their assembly on target mRNAs and the role of mRNA elements in mediating translation regulation within the cell. Such studies will be required to fully understand the individual contributions of eIF4F components to translation initiation.

## Conclusions

Here we provide the first genome-wide profile of the translational signature of eIF4A and show that this helicase regulates the translation of specific mRNAs involved in proliferation, survival and metastasis, consistent with the notion that eIF4F acts at an important node in tumorigenesis. We also find that the population of eIF4A-dependent mRNAs is distinct from those that depend on 4E-BP-mediated assembly of eIF4E and eIF4G. In support of this notion we observed that catalytic mTOR inhibitors, which block the assembly of eIF4F, additively blocked cell proliferation when used in combination with Silvestrol. These observations raise the possibility that anti-cancer therapies aimed at blocking translation would be more effective if multiple governing factors were inhibited in combination. Additionally, the effect of a single agent targeting the translation apparatus could be overcome by increased activity of a parallel pathway, thereby increasing the likelihood of acquired resistance. We propose that eIF4F target mRNAs have differential requirements for individual components of the complex, and possibly other initiation factors, that allow for dynamic regulation of their translation. If this were true, alterations to the balance of each subunit in eIF4F could uniquely influence malignant transformation by impacting the translation of discrete mRNA subsets. This could lead to altered proteomic landscapes with signatures that differ depending on which eIF4F component drives transformation. Genome-wide dissection of the translational output of individual eIF4F constituents and other initiation factors will be critical for our understanding of malignant progression and will guide future efforts aimed at targeting this central hub in cancer signaling. Moreover, further analyses of cancer-specific translational profiles will likely uncover novel biomarkers for malignant subtypes and enlighten the mechanisms of resistance to current cancer therapies.

## Materials and methods

### Cell proliferation assays

Cells were seeded at a density of 1 × 10^3^ cells/well in 96-well plates and treated with Silvestrol at the following final concentrations: 3, 1, 0.3, 0.1, 0.03, 0.01, and 0 μM. Plates were incubated for 3 days and cell viability was measured using CellTiter-Glo luminescent viability reagent (Promega, Sunnyvale, CA USA).

### Metabolic labeling

MDA-MB-231 cells were seeded at a density of 3 × 10^5^ cells/well in 6-well format and grown overnight. 24 hours after seeding, cells were washed in 1× phosphate-buffered saline (PBSand media was replaced with RPMI lacking methionine; cells were incubated in the absence of methionine for 1 hour. Methionine-free medium containing Silvestrol was added to each well and cells were pulsed with 0.1 mCi/ml ^35^S-methionine/cysteine (EasyTag EXPRESS^35^S Protein Labeling Mix, Perkin Elmer #NEG77200, Santa Clara, CA, USA) for 10 minutes prior to harvest. Cells were lysed in RIPA buffer (50 mM Tris-HCl pH7.5, 150 mM NaCl, 2 mM EDTA pH8, 1% TritonX-100, 1% sodium deoxycholate, 0.1% SDS) containing complete EDTA-free protease inhibitors (Roche #11836170001, Indianapolis, IN, USA), and phosphatase inhibitors (PhosSTOP, Roche #04906837001). Total protein was trichloroacetic acid (TCA precipitated and the radioactive counts were measured by scintillation counting. Protein content in cell lysates was quantified using BCA reagent (Thermo Scientific #23228, Rockford, IL, USA) and used to normalize radioactive counts in TCA precipitated protein.

### Polysome analysis and fractionation

Cells were seeded at a density of 4 × 10^6^ cells/plate in 15 cm plates and grown overnight. Cells were then treated with DMSO or Silvestrol (25 nM) for 2 hours prior to harvest. At time of harvest, 100 μg/ml cycloheximide (Sigma-Aldrich, St. Louis, MO, USA) was added to each plate and incubated for 1 minute. Cells were washed twice in ice-cold 1× PBS +100 μg/ml cycloheximide and lysed in ice-cold polysome lysis buffer (12.5 mM Tris pH7, 7.5 mM Tris pH8, 15 mM MgCl_2_, 200 mM NaCl, 1 mM DTT, 8% glycerol, 100 μg/ml cycloheximide, 24 U/ml Turbo DNase I (Life Technologies #AM2238, Grand Island, NY, USA), 1% TritonX-100 ). Sucrose gradients (10 to 50%) were created by layering 10% and 50% sucrose solutions (15 mM Tris pH8, 100 mM KCl, 3 mM MgCl_2_, 1 mM DTT, 100 μg/ml cycloheximide, 20 U/ml Superase Inhibitor (Ambion #AM2696), 10 or 50% RNase-free sucrose) into Seton tubes (Seton Scientific #7030, Los Gatos, CA, USA) followed by mixing with the BioComp Gradient maker set at an 81.5° angle at 16 rpm (BioComp Instruments, Fredericton, NB, Canada). Lysates were layered on top of each gradient and subjected to ultracentrifugation in an SW41-Ti rotor at 35,000 rpm at 4°C for 3 hours. Polysome profiles were analyzed using a BioComp Gradient Station at constant speed with optical monitoring at a 260 nm wavelength. For polysome fractionation experiments, 1 ml fractions were collected and stored at -80°C for subsequent RNA isolation followed by quantitative RT-PCR.

### Preparation of samples for ribosome profiling

Ribosome profiling was performed as previously described [11,41] with some modifications. Briefly, MDA-MB-231 cells were treated with Silvestrol (25 nM for 1or 2 hours), DMSO (1:4,000 for 1 or 2 hours) or INK128 (200 nM for 2 hours). After treatment cells were washed twice in cold PBS containing cycloheximide (100 μg/ml) and lysed in lysis buffer (20 mM Tris pH7, 1% TritonX-100, 220 mM NaCl, 15 mM MgCl_2_, 1 mM DTT, 8% glycerol, 100 μg/ml cycloheximide (Sigma), 24 U/ml Turbo DNAse (Ambion)). Lysates were clarified, treated with RNAse I (Ambion) and overlaid on a 34% sucrose cushion. Monosomes were isolated by centrifugation at 69,000 rpm for 4 hours in a TLA110 rotor. Ribosome protected RNA fragments (RFs) were isolated from monosome fractions by acidic-phenol extraction and used to generate libraries for sequencing. The genome-aligned RF profiles in Additional file 3 represent the count of bases occupying the ribosomal P-site, calculated by mapping the p-offset for sequencing reads.

### Preparation of samples for mRNA-Seq

Total RNA was extracted from MDA-MB-231 cells using Trizol (Life Technologies #15596026, Grand Island, NY, USA) following the manufacturer’s guidelines. Polyadenylated RNA was isolated from the total fraction using Oligotex mRNA kit (Qiagen #70022, Germantown, MD, USA). The resulting mRNA was partially fragmented by alkaline hydrolysis with sodium carbonate to generate approximately 150-nucleotide fragments on average. RNA fragments of 40 to 100 nucleotides were isolated by gel extraction and used to generate libraries for mRNA-Seq. The genome-aligned mRNA profiles in Additional file 3 represent counts of the 5′-terminal bases of sequencing reads.

### Library generation and sequencing

Strand-specific libraries were generated as described [11,41] with modifications described in Stern-Ginnosaur *et al*. [13]. Samples were sequenced on the Illumina HiSeq 2000 using the TruSeq SBS Kit v3 50 cycles (Illumina #FC-401-3002, San Diego, CA, USA), yielding single end reads that were 50 base pairs in length.

### Sequencing data analysis

Sequence analysis was performed as described [13]. Briefly, linker and polyA sequences were removed from the 3′ end reads prior to alignment. Reads were then aligned with Bowtie [42] with no more than two mismatches allowed end-to-end (-v2) and with reporting up to 16 alignments (-a –m -16). Sequences aligning to rRNA were discarded and the remaining reads were aligned in parallel to the human genome (hg19) as well as to UCSC KnownGene canonical transcripts [43]. These alignments were merged and uniquely mapped reads were counted to calculate the mRNA and ribosome footprint RPKM value. Genomic positions with non-uniquely aligned reads as well as positions that overlapped with more than one KnownGene canonical transcript were excluded from the RPKM calculation. When computing counts for mRNA-Seq data, the count was assigned to the genomic position corresponding to the middle of each read. For RF-Seq reads, counts were assigned to the genomic location that corresponded to the 5′-most base present in the P-site of the ribosome which protected the mRNA fragment from RNase I digestion. The location of the P-site in RF reads was calculated based on the mapped distance from the 5′-terminal base to the base occupying the P-site of the translating ribosome [11,13,41]. The RPKM values per gene for mRNA-Seq and RF-Seq were visualized using Spotfire Analytic software (TIBCO, Boston, MA, USA). The genome-aligned RF profiles in Additional file 3 represent the count of bases occupying the ribosome P-site; corresponding mRNA profiles represent counts of the 5′ terminal bases of sequenced reads.

### 5′ UTR analysis

5′ UTR sequences were retrieved from Ensemble using RefSeq mRNA numbers as query. Folding energies of the resulting sequences were analyzed using CONTRAfold [25] and McCaskill [44] RNA structure prediction algorithms.

### Reporter constructs

The CMV promoter was amplified by PCR and cloned into the SacI restriction site of pGL4.25 to create vector pGL4.25CMV. The promoter was subsequently mutagenized at a single site to remove an internal NcoI site and create vector pMH2. 5′ UTR sequences from *CyclinD1* and *ROCK1* were reverse transcribed from total RNA isolated from MDA-MB-231 cells (SuperScript III, Invitrogen #18080-051) and the resulting cDNA was amplified by high-fidelity PCR using 5′ UTR-specific oligonucleotides. The 5′ UTRs for *ARF6*, *ARF6mut* and *PFN2* were synthesized by Blue Heron Biotechnology (Bothell, WA, USA). 5′ UTRs were subcloned into the NcoI site of vector pMH2 to create vectors pCR300, pCR301, pCR302, pCR303, and pCR304 (Table S5 in Additional file 2).

### Luciferase assays

293 T cells were co-transfected with 5′ UTR-bearing *luc2CP* vectors plus pBabePuro at a ratio of 20:1. Medium was replaced with DMEM +2 μg/ml puromycin 48 hours post-transfection and cells were grown to select for puromycin-resistant colonies. Stably transfected cells were seeded in 96 well plates at 4 × 10^4^ cells/well and allowed to settle overnight. Medium was removed and replaced with 100 μl/well of medium containing 0, 12.5, 25, 50, 100 or 200 nM Silvestrol or 0, 0.03125, 0.0625, 0.125, 0.25, 0.5, 1, 2, 4 μM hippuristanol and incubated for 40 minutes. Silvestrol-containing medium was removed and cells were lysed in 20 μl/well Passive Lysis Buffer (Promega E194A). Luciferase assays were performed by adding 100 μl/well Luciferase Assay Reagent (Promega E1501) and incubating for 10 minutes at room temperature before measuring luminescence. For *luc2CP* mRNA analysis, 4 × 10^5^ cells/well were seeded in 6-well plates and grown overnight. Cells were then treated with the indicated amounts of Silvestrol for 40 minutes and lysed using Trizol reagent. RNA was isolated from Trizol lysates using standard methods. The abundance of *luc2CP* and *β-actin* mRNA was subsequently analyzed by quantitative RT-PCR.

### RNA isolation from polysome fractions

Prior to RNA extraction from polysome fractions, 5 ng of Luciferase Control RNA (Promega) was added to each fraction. Samples were treated with 200 μg/ml Proteinase K for 1 hour at 50°C and RNA was extracted using the standard hot acid phenol method. RNAs were precipitated using isopropanol and resuspended in 10 mM Tris pH7.

### Quantitative RT-PCR

Total RNA was converted to cDNA using SuperScript III reverse transcriptase (Invitrogen) with oligo dT primers according to the manufacturer’s instructions. Transcript levels were measured by quantitative PCR using SYBR green FAST PCR mix (Applied Biosystems, Foster City, CA) and oligo pairs specific to each transcript. In polysome fractionation experiments, transcript levels were normalized to luciferase control RNA abundance. To measure luciferase reporter mRNA (*luc2CP* mRNA) levels, transcript abundance was normalized to *β-actin* mRNA measurements.

### Cell cycle assays

MDA-MB-231 cells were seeded in 6-well dishes at 3.5 × 10^5^ cells/well and grown overnight. At the start of the experiment, growth medium was replaced with medium containing 25 nM Silvestrol. Cells were pulsed with 1 μM BrDU for 30 minutes before harvesting by trypsinization at the indicated time points. To measure sub-G1 populations, medium was collected along with adherent cells at time of harvest. BrDU-pulsed cells were pelleted, washed in 1× PBS and fixed using ice-cold 70% ethanol. Fixed cells were permeabilized on ice in 1× PBS containing 0.1 M HCl and 0.5% Triton X-100, washed and then boiled for 10 minutes. Cells were then incubated with 5 μg/ml anti-BrDU-fluorescein isothiocyanate (FITC) antibody (BD Biosciences #347583, San Jose, CA, USA) at room temperature for 30 minutes, washed, resuspended in PI/RNase solution (BD Biosciences #550825) and analyzed by fluorescence-activated cell sorting (FACS).

### Western blotting

MDA-MB-231 cells were treated with 25 nM Silvestrol and lysed in RIPA buffer (50 mM Tris-HCl pH7.5, 150 mM NaCl, 2 mM EDTA pH8, 1% TritonX-100, 1% sodium deoxycholate, 0.1% SDS) containing complete EDTA-free protease inhibitors (Roche #11836170001), and phosphatase inhibitors (PhosSTOP, Roche #04906837001). Lysates were quantified using BCA reagent (Thermo Scientific #23228). Lysates were subjected to SDS-PAGE (NuPAGE) using 15 to 25 μg of cell lysate per well and transferred to PVDF (Invitrogen).

### Antibodies

The following antibodies were purchased from commercial suppliers: CyclinD1 (Cell Signaling #2926, Beverly, MA, USA), CyclinB1 (Cell Signaling #4135), CyclinE1 (Cell Signaling #4129), Bcl2 (BD Pharmingen #551097), Parp(Asp214) (Cell Signaling #9541), Arf6 (Cell Signaling #5740), GAPDH (Santa Cruz Biotechnology#32233, Santa Cruz, CA, USA), β-Tubulin (Cell Signaling #2146).

### Cell lines

Cell lines used in this study were obtained from ATCC. Cells were tested for mycoplasma by the Novartis Institutes for Biomedical Research and were mycoplasma-free.

### Oligonucleotides

#### For amplifying 5′ UTRs

oARF6fwd: 5′^_^CATCTCCATGGGCAGAACTGGGAGGAGGAGT^_^3′; oARF6rev: 5′^_^GACCTCCATGGCGCGTCGGAGGAGCCGGGGCCG^_^3′; oCCND1fwd:_5′^_^GACCTCCATGGGCTTAACAACAGTAACGTCACACGG^_^3′; oCCND1rev:_5′^_^CAGCTCCATGGCTGGGGCTCTTCCTGGGCAG^_^3′; oROCK1fwd:_5′^_^GACCTCCATGGGCGCUGGUUCCCCTTCCGAGCGT^_^3′; oROCK1rev:_5′^_^CATCTCCATGGGTGTTGCTGCTGCTGTGACAATGCCCT^_^3′; oPFN2fwd:_5′^_^GAATCTCCATGGGCCGCTGGTTTGTCAGCC^_^3′; oPFN2rev:_5′^_^CTCGAGCCATGGCTTCGAGCCCTTCGC^_^3′; oARFmut_ampF:_5′^_^CCATGGTTGGGACGTGCACTGGCAGCCGGC^_^3′; oARFmut_ampR:_5′^_^GTGGGAGTTGCCTCCTAAGCTAATATGTGC^_^3′

#### For quantitative PCR

ARF6_qP_F8: 5′^_^ATGTTGCAGGTGAGATGTGGT^_^3′,

ARF6_qP_R8: 5′^_^TACCTGCTCCAGTCACCAATG^_^3′,

ActB_qP_F1: 5′^_^AGCCTCGCCTTTGCCGA^_^3′,

ActB_qP_R1: 5′^_^GCGCGGCGATATCATCATC^_^3′,

BCL2_qP_F3: 5′^_^TTCTGCCCCTGCCAAATCTT^_^3′,

BCL2_qP_R3: 5′^_^CATCTGAGAACCTCCTCGGC^_^3′,

CCND1_qP_F2: 5′^_^TGAGGGACGCTTTGTCTGTC^_^3′,

CCND1_qP_R2: 5′^_^CTTCTGCTGGAAACATGCCG^_^3′,

CDK6_qP_F2: 5′^_^ACCTGCCCCTTACTCTGACT^_^3′,

CDK6_qP_R2: 5′^_^AGCACCCAGTAAGACATCCAG^_^3′,

ROCK1_qP_F1: 5′^_^TGAAAGCCGCACTGATGGAT^_^3′,

ROCK1_qP_R1: 5′^_^GCCATGAGAAAACACATTGCAG^_^3′,

Luciferase_F: 5′^_^ATCCGGAAGCGACCAACGCC^_^3′,

Luciferase_R: 5′^_^GTCGGGAAGACCTGCCACGC^_^3′,

Luc2CP_F: 5′^_^ATCCACCTTAACAGCCACGG^_^3′,

Luc2CP_R: 5′^_^CAGGGTGTCTATCCATGCCG^_^3′.

#### 5′ UTR sequences

Mutated residues are indicated by lower case lettering in the *ARF6mut* sequence. The (CGG)_4_ motif is shown in bold.

*ARF6wt*:_5′^_^AGAACUGGGAGGAGGAGUUGGAGGCCGGAGGGAGCCCGCGCUCGGGGCGGCGGCUGGAGGCAGCGCACCGAGUUCCCGCGAGGAUCCAUGACCUGACGGGGCCCCGGAGCCGCGCUGCCUCUCGGGUGUCCUGGGUCGGUGGGGAGCCCAGUGCUCGCAGGCCGGCGGGCGGGCCGGAGGGCUGCAGUCUCCCUCGCGGUGAGAGGAAGGCGGAGGAGCGGGAACCGCGGCGGCGCUCGCGCGGCGCCUGCGGGGGGAAGGGCAGUUCCGGGCCGGGCCGCGCCUCAGCAGGGCGGCGGCUCCCAGCGCAGUCUCAGGGCCCGGGUGGCGGCGGCGACUGGAGAAAUCAAGUUGUGCGGUCGGUGAUGCCCGAGUGAGCGGGGGGCCUGGGCCUCUGCCCUUAGGAGGCAACUCCCACGCAGGCCGCAAAGGCGCUCUCGCGGCCGAGAGGCUUCGUUUCGGUUUCG**CGGCGGCGGCGG**CGUUGUUGGCUGAGGGGACCCGGGACACCUGAAUGCCCCCGGCCCCGGCUCCUCCGACGCGCCAUG^_^3′.

*ARF6mut*:_5′^_^CCAUGGuUGGGAcGuGcAcUgGcAGcCgGcAGaGAGCCCGCcCaCcGcuacGCGGCUuacccgucCcgAgCcAcUaggCcCGAGGAUCCAUGACCUGACGGGGCCCCGGAGCCGCGCUGCCUCUCGGGUGUCCUGGGUCGGUGGGGAGCCCAGUGCUCGCAGGCCGGCGGGCGGGCCGGAGGGCUGCAGUCUCCCUCGCGGUGAGAGGAAGGCGGAGGAGCGGGAACCGCGGCucuagaCGCGCGGCGCCUGCGGGGGGAAGGGCAGUUCCGGGCCGGGCCGCGCCUCAGCAGGGCGGCGGCUCCCAGCGCAGUCUCAGGGCCCGGGUGGCGGCGGCGACUGGAGAAAUCAAGUUGUGCGGUCGGUGAUGCCCGAGUGAGCGuauacagUcGaCaUaUuagCUUAGGAGGCAACUCCCACGCAGGCCGCAAAGGCGCUCUCGCGGCCGAGAGGCUUCGUUUCGGUUUCG**CGGCGGCGGCGG**CGUUGUUGGCUGAGGGGACCCGGGACACCUGAAUGCCCCCGGCCCCGGCUCCUCCGACGCGCCAUG^_^3′.

*CyclinD1*:_5′^_^CCAUGGGCUUAACAACAGUAACGUCACACGGACUACAGGGGAGUUUUGUUGAAGUUGCAAAGUCCUGGAGCCUCCAGAGGGCUGUCGGCGCAGUAGCAGCGAGCAGCAGAGUCCGCACGCUCCGGCGAGGGGCAGAAGAGCGCGAGGGAGCGCGGGGCAGCAGAAGCGAGAGCCGAGCGCGGACCCAGCCAGGACCCACAGCCCUCCCCAGCUGCCCAGGAAGAGCCCCAGCCAUG^_^3′.

*ROCK1*:_5′^_^GCUGGUUCCCCUUCCGAGCGUCCGCGCCCCGCAUGCGCAGUCUGCCCCGGCGGUCUCCGUUUGUUUGAACAGGAAGGCGGACAUAUUAGUCCCUCUCAGCCCCCCUCGCCCCACCCCCCAGGCAUUCGCCGCCGCGACUCGCCCUUUCCCCGGCUGGGACCGCAGCCCCUCCCAGAAGCUCCCCCAUCAGCAGCCGCCGGGACCCAACUAUCGUCUUCCUCUUCGCCCGCUCUCCAGCCUUUCCUCUGCUAAGUCUCCAUCGGGCAUCGACCUCGCCCUGCCCCACCGGACACCGUAGCAGCAGCCCCAGCAGCGACGGGACAAAAUGGGAGAGUGAGGCUGUCCUGCGUGGACCAGCUCGUGGCCGAGACUGAUCGGUGCGUCGGGCCGGGCCGAGUAGAGCCGGGGACGCGGGGCUAGACCGUCUACAGCGCCUCUGAGCGGAGCGGGCCCGGCCCGUGGCCCGAGCGGCGGCCGCAGCUGGCACAGCUCCUCACCCGCCCUUUGCUUUCGCCUUUCCUCUUCUCCCUCCCUUGUUGCCCGGAGGGAGUCUCCACCCUGCUUCUCUUUCUCUACCCGCUCCUGCCCAUCUCGGGACGGGGACCCCUCCAUGGCGACGGCGGCCGGGGCCCGCUAGACUGAAGCACCUCGCCGGAGCGACGAGGCUGGUGGCGACGGCGCUGUCGGCUGUCGUGAGGGGCUGCCGGGUGGGAUGCGACUUUGGGCGUCCGAGCGGCUGUGGGUCGCUGUUGCCCCCGGCCCGGGGUCUGGAGAGCGGAGGUCCCCUCAGUGAGGGGAAGACGGGGGAACCGGGCGCACCUGGUGACCCUGAGGUUCCGGCUCCUCCGCCCCGCGGCUGCGAACCCACCGCGGAGGAAGUUGGUUGAAAUUGCUUUCCGCUGCUGGUGCUGGUAAGAGGGCAUUGUCACAGCAGCAGCAACACCCAUG^_^3′

*PFN2*:_5′^_^CGCUGCGGUAAGGAGCAGCCGCCACAGGCACAGCCGCUUCGCAGCCUCCCGCCGCUGGUUUGUCAGCCCCGCGGCUGCGGGCGGCCGGGCGGCCGAGCGCGCUCUGAGGUUCGUCCCUCAUCGCUGAACCCGCGUCCUCCCGCCGCAGCUCCUCGGGGAGGGGGGCGGUCGGUGCCUGCGCAGAGCCGCCUCCUCCCCGCCCCCGCCCCGCCUCCCCCCGCGCCGCCGCCGCCCGCUACCGCCGCCGCCGCCGCUGCGCCUGCUGCUCCUCGCCGUCCGCGCUGCAGUGCGAAGGGCUCGAGCCAUG^_^3′.

### Mass spectrometry

#### Digestion of monosome pellets

Monosome preparations pelleted in ultracentrifuge tubes were washed with 1 ml ice-cold ethanol by addition of the ethanol followed by a 5 minute incubation on ice and aspiration of the ethanol via a vacuum trap. The pellet was allowed to air dry. Monosome pellet was dissolved in 100 μl of a solution of 20 mM ammonium bicarbonate, 8 M urea and 2 mM Tris(2-carboxyethyl)phosphine hydrochloride. MS-Grade Lys C (Thermo) was dissolved in water to a concentration of 0.5 μg/μl and 2 μl was added to the dissolved pellet. The Lys C digestion was incubated for 4 hours at room temperature. The digestion was further diluted with an additional 98 μl of water. MS-Grade Trypsin (Thermo) was also dissolved in water to a concentration of 0.5 μg/μl and 2 μl was added to the diluted Lys C digestion. This trypsin digestion was incubated overnight at room temperature.

#### Microscale LC-MS/MS and mascot ions search of trypsin digestion samples

Digestion samples were prepared for liquid chromatography-tandem mass spectrometry (LC-MS/MS) by acidification. The digestion sample (20 μl) was acidified by the addition of 2 μl of 10% formic acid; 20 μl of this sample was injected onto a 2.1 × 50 mm Extend-C18 column (1.8 μm beads; Agilent, Santa Clara, CA, USA) at 400 μl/minute flow rate. Elution of peptides was over a 51 minute gradient from 5 to 40% acetonitrile with 0.1% formic acid as the modifier, and a flow rate of 200 μl/minute. Data acquisition was on an Agilent 6530 Q-ToF instrument equipped with a dual electrospray source, and a reference mass of 922.009798 enabled. MS/MS spectra were acquired in Auto MS/MS mode with triggering of precursor ions of +2 charge state and higher and individual ions were excluded for 9 seconds after triggering an MS/MS scan. Up to five precursors were selected per MS scan, and MS/MS scans were for 50,000 counts or 333 mS, whichever came first. LC-MS/MS data were exported to a Mascot Generic Format file using MassHunter Qual 5.0 (Agilent) and searched against SwissProt 51.6 with taxonomy limited to human, without modifications other than variable pyroglutamate formation at Q and E, MS tolerance of 10 ppm and MS/MS tolerance of 0.1 Da.

#### Nano LC-MS/MS and mascot ions search of trypsin digestion samples

Digestion samples were prepared for LC-MS/MS by acidification. The digestion sample (10 μl) was acidified by the addition of 10 μl of 1% formic acid; 4 μl of this sample was injected onto a 4 mm 40 nL trap column at a flow rate of 3 μl/minute and analyzed on a 75 μm × 150 mm column containing Zorbax 300SB-C18 (5 μm beads) at a flow rate of 400 nl/minute. Elution of peptides was over a 100 minute gradient from 5 to 40% acetonitrile with 0.1% formic acid as the modifier. Data acquisition was on an Agilent 6550 Q-ToF instrument equipped with the ChipCube source, and a reference mass of 1221.990637 enabled. MS survey scans were acquired at a rate of 5 Hz, and MS/MS spectra were acquired in Auto MS/MS mode with triggering of precursor ions of +2 charge state and higher. Individual ions were excluded for 18 seconds after triggering an MS/MS scan. Up to five precursors were selected per MS scan, and MS/MS scans were for 25,000 counts or 200 mS, whichever came first. LC-MS/MS data were exported to a Mascot Generic Format file using MassHunter Qual 5.0 and searched against SwissProt 51.6 with taxonomy limited to human, without modifications other than variable pyroglutamate formation at Q and E, MS tolerance of 5 ppm and MS/MS tolerance of 0.1 Da.

### Sliding window analysis

5′ UTR sequences were retrieved from Ensemble using RefSeq mRNA names as query. Sequences were broken into 20-nucleotide fragments in a stepwise manner beginning from the first nucleotide (n) and proceeding in 1 nucleotide steps (n +1, n +2, n +3, and so on) through the length of the UTR. The structure of each fragment was analyzed using the CONTRAfold algorithm and the resulting free energy values were plotted based on their position across the length of the UTR, as were the percentage GC values.

### Trans-well migration assay

Migration assays were performed using trans-well migration chambers (BD Biosciences). MDA-MB-231 cells were seeded into upper chambers at a density of 0.5 × 10^5^ cells in serum-free medium. Medium containing 5% fetal bovine serum was aliquoted to lower wells and cells were incubated for 22 hours. When measuring migration in the presence of Silvestrol, compound was included in the medium in both upper and lower chambers at the concentrations indicated. To assess cell migration after 22 hours, upper chambers were transferred to wells containing 1× PBS +8 μM Calcenein AM (Life Technologies) and incubated for 40 minutes. Fluorescence was evaluated by excitation at 485 nM wavelength followed by measuring emission at 520 nM.

### Scratch wound assay

Cells were seeded into 24 well plates at a density of 6 × 10^5^ cells/well in RPMI supplemented with 10% fetal bovine serum and grown for 24 hours to generate a monolayer. The following day cells were scratched to produce a wound in the monolayer and wound closure was monitored by microscopy using the Incucyte Live Cell Imaging system (Essen Biosciences, Ann Arbor, MI, USA).

### Data availability

The next generation sequencing data presented in this paper have been deposited in NCBI’s Gene Expression Omnibus [45] and are accessible through GEO Series accession number GSE61375 [46]. The mass spectrometry data have been deposited to the ProteomeXchange Consortium [47] via the PRIDE partner repository [48] with the dataset identifier PXD001310 and DOI 10.6019/PXD001310.

## Additional files

Additional file 1: Figure S1. MDA-MB-231 polysome profiles in the presence of translation inhibitors. MDA-MB-231 cells were treated with DMSO, 25 nM Silvestrol or 1 μg/ml harringtonine for 30 minutes. Lysates were fractionated by ultracentrifugation through a sucrose gradient and polysome distribution was analyzed by monitoring absorbance of light at 260 nm (A_260_). Additional file 2: Table S1. Mass spectrometry of monosome fractions from MDA-MB-231 cells. LC-MS/MS mass spectrometry was performed using monosome fractions from **(a)** DMSO- or **(b)** Silvestrol-treated MDA-MB-231 cells. Proteins were identified by Mascot using peptide sequences as query; those with 98% confidence or greater are listed for each replicate sample. **Table S2: Genes showing reduced TE in Silvestrol**. Table of 284 genes identified by ribosome profiling as having reduced TE in the presence of 25 nM Silvestrol. Genes are organized based on fold change in TE (∆TE) values. **Table S3: Genes showing increased TE in Silvestrol**. Table of 146 genes identified by ribosome profiling as having increased TE in the presence of 25 nM Silvestrol. Genes are organized based on fold change in TE (∆TE) values. **Table S4: 5′ UTR characteristics of Silvestrol-sensitive and -insensitive genes.** Table showing free energy values (∆G), length and percentage GC content for 5′ UTRs from genes that are sensitive (decreased ∆TE) or insensitive to 25 nM Silvestrol. ∆G values were predicted using two algorithms, CONTRAfold or McCaskill [44]. The correlation coefficient for these data sets is r =0.914. Genes bearing variant 5′ UTR isoforms are indicated with an asterisk. **Table S5: Table of vectors used in luciferase reporter assays.** Vector pMH2 was constructed from pGL4.25 with CMV promoter in place of the minimal promoter. Vectors pCR300, pCR301, pCR302, pCR303, and pCR304 were constructed by adding the indicated 5′ UTRs to vector pMH2. See Materials and methods for cloning information. **Table S6: Genes showing reduced TE in INK128**. Table of 244 genes that showed reduced TE in MDA-MB-231 cells treated with 200 nM INK128 for 2 hours. The gene also found to have reduced TE after Silvestrol treatment is indicated by asterisks. Additional file 3: Figure S2. Select data from ribosome profiling sequencing. **(a)** The frequency of read lengths from RNA-Seq libraries generated by ribosome footprinting (RF). **(b)** Table of sequencing statistics for sample generated by next-generation sequencing. Coverage was computed using the Picard HsMetrics tool for every exon in the KnownGene database. Exons with fewer than two reads were excluded. Abbreviations: br, biological replicate; RF, ribosome footprint. **(c)** Scatter plot of RF densities (measured in RPKM) in MDA-MB-231 cells treated with 25 nM Silvestrol versus DMSO for 1 hour. Silvestrol-sensitive genes are indicated in dark blue. Additional file 4: Figure S3. Polysome analysis of select Silvestrol-sensitive transcripts. Polysomes from 25 nM Silvestrol- or DMSO-treated cells were fractionated and analyzed for the abundance of transcripts for *β-actin*, *CyclinD1*, *ARF6*, *ROCK1*, *CDK6* and *BCL2*. Data presented are mean values ± standard error (n =2). Additional file 5: Figure S4. Structured 5′ UTRs from Silvestrol-sensitive transcripts, used to evaluate 5′ UTR contribution to translation of a luciferase reporter. **(a)** Free energy values for the 5′ UTRs of Silvestrol-sensitive genes; genes are separated into three classes based on TE values: Decreased TE (n =284), insensitive (n =299) and increased TE (n =146). Energy values were predicted using the CONTRAfold algorithm. The pool of genes with decreased TE values are enriched with structured 5′ UTRs. **(b)** Luciferase production from *CMV*-driven *luc2CP* (pMH2) in the presence of cycloheximide (100 μg/ml) or Silvestrol (50 nM) over time. Half-life of Luc2CP protein is 30.99 minutes. In each experiment, samples were assayed in triplicate; data were derived from two independent experiments. **(c-e)** Plots of sequencing data derived from genes selected for 5′ UTR assessment. Data represent the non-normalized frequency of either the 5′end (mRNA) or P-site (RF) of each read found in sequencing data. **(f)** *Luc2CP* mRNA levels in Silvestrol-treated 293 T cells bearing the indicated reporter constructs. *Luc2CP* abundance was measured by quantitative PCR and normalized to *β-actin* mRNA levels. Data represent mean values ± standard error of the mean (n =3). Additional file 6: Figure S5. Luciferase expression from 5′ UTR reporter constructs upon treatment with hippuristanol. **(a)** Luciferase reporter constructs, stably transfected into 293 T cells, were treated with increasing concentrations of hippuristanol and luciferase expression was measured after 40 minutes; constructs bearing 5′ UTRs from Silvestrol-sensitive genes (*CyclinD1*, *ROCK1*, *ARF6*) or insensitive genes (*PFN2* or *CMV* alone) were compared. **(b)** Luciferase expression from reporter constructs bearing *ARF6wt* 5′ UTR or *ARF6mut* 5′ UTR, stably transfected into 293 T cells. Cells were treated with increasing concentrations of hippuristanol and measurements were taken after 40 minutes of exposure. Data presented were obtained from two independent experiments with measurements taken in triplicate. Additional file 7: Figure S6. Sliding window structure analysis of select 5′ UTRs. **(a,b)** Free energy values (a) and percentage GC content (b) of 20-nucleotide fragments of the 5′ UTR of *CyclinD1* plotted along the length of the UTR from the TSS to the translation start (AUG). **(c-f)** Free energy values (c,e) and percentage GC content (d,f) of the 5′ UTR of *ARF6* (c,d) and *ARF6mut* (e,f) plotted along the length of the UTR from the TSS to the translation start (AUG). Each asterisk denotes a structured region that was mutated in *ARF6* to create *ARF6mut*. Additional file 8: Figure S7. Silvestrol treatment impairs migration of MDA-MB-231 cells. **(a)** Migration of MDA-MB-231 cells was measured in a trans-well migration assay in the presence of increasing amounts of Silvestrol. Plots represent Calcenein AM fluorescence of cells which migrated across a barrier. **(b)** MDA-MB-231 cells were grown to a confluent monolayer and scratched to produce a wound. Wound closure was monitored over time by microscopy. **(c)** Quantification of wound closure (represented in (b)) over time. **(d)** Western blot for Arf6 protein after treatment with 25 nM Silvestrol. Additional file 9: Figure S8. Translation of Silvestrol-sensitive transcripts is not disrupted by mTOR inhibition. **(a)** MDA-MB-231 cells were treated with vehicle (DMSO) or 200 nM INK128 for the indicated times and Western blots were performed for Arf6 (top panels) and Cyclin D1 (bottom panels). **(b)** PC-3 cells were treated with vehicle (DMSO), 200 nM INK128 or 25 nM Silvestrol for the indicated times. Western blots were performed for Arf6, Cyclin D1 and phosphorylated 4E-BP1/2 (Ser65). Additional file 10: Figure S9. Combinatorial treatment with Silvestrol and mTOR inhibitors show additivity in blocking cancer cell proliferation. **(a,b)** Isobolograms (top panels) and dose matrices (bottom panels) of MDA-MB-231 (a) and PC-3 (b) cells treated with serial combinations of Silvestrol and INK128. **(c,d)** MDA-MB-231 (c) or PC-3 (d) cells were treated with increasing concentrations of Silvestrol in combination with PP242 at fixed doses: 50 nM, 125 nM and 250 nM. Cell viability was measured by CellTiter-Glo after 3 days. **(e,f)** Isobolograms (top panels) and dose matrices (bottom panels) of MDA-MB-231 (e) and PC-3 (f) cells treated with serial combinations of Silvestrol and PP242.

## Abbreviations

BrDU: bromodeoxyuridine
DMEM: Dulbecco's modified Eagle medium
DMSO: dimethyl sulfoxide
DTT: dithiothreitol
eIF4F: eukaryotic initiation factor 4 F
GO: gene ontology
LC-MS/MS: liquid chromatography-tandem mass spectrometry
MS: mass spectrometry
MS/MS: tandem mass spectrometry
mTOR: mammalian target of rapamycin
PBS: phosphate-buffered saline
PI: propidium iodide
RF: ribosome footprint
RPKM: reads per kilobase per million
TCA: trichloroacetic acid
TE: translation efficiency
TSS: transcription start site
UTR: untranslated region
