# Supplementary figures and images for: Transcriptome-wide characterization of the eIF4A signature highlights plasticity in translation regulation
Source: Genome Biol. 2014 Oct 2;15(10):476. doi: 10.1186/s13059-014-0476-1 (PMC4203936; doi:10.1186/s13059-014-0476-1)

Figure S1

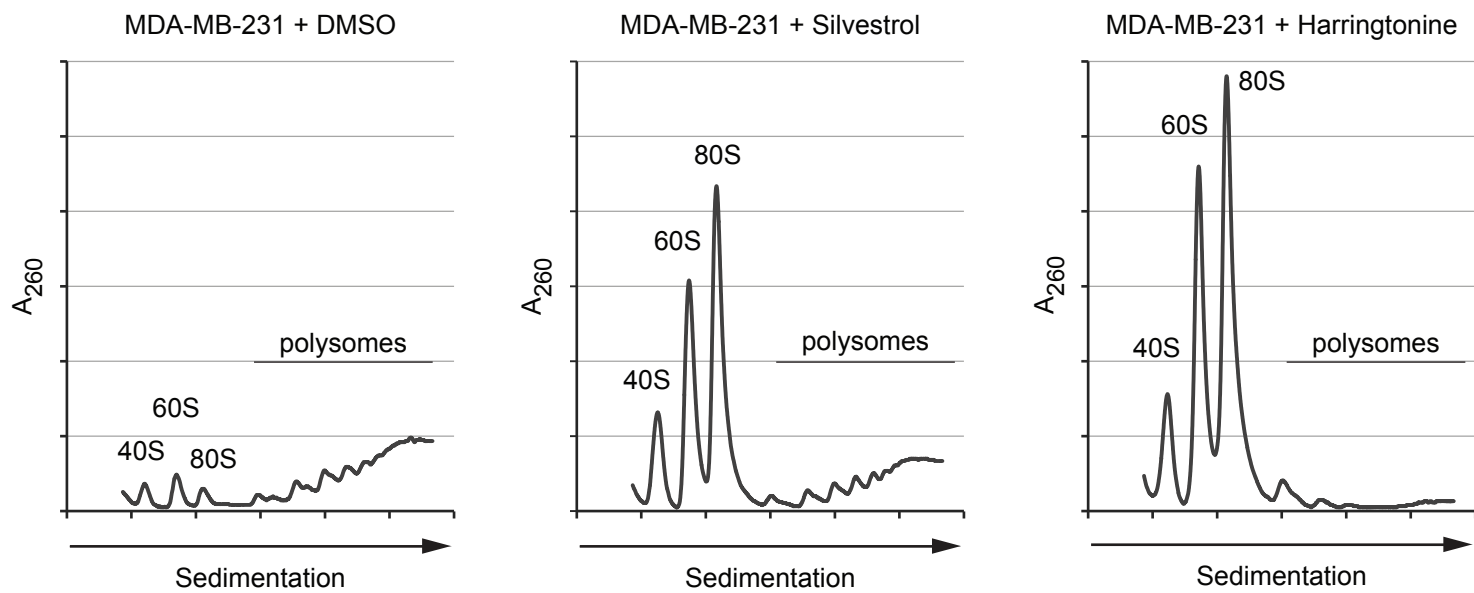

Supplement: Additional file 1: Figure S1. — MDA-MB-231 polysome profiles in the presence of translation inhibitors. MDA-MB-231 cells were treated with DMSO, 25 nM Silvestrol or 1 μg/ml harringtonine for 30 minutes. Lysates were fractionated by ultracentrifugation through a sucrose gradient and polysome distribution was analyzed by monitoring absorbance of light at 260 nm (A260). [file 13059_2014_476_MOESM1_ESM.pdf]

Figure S2

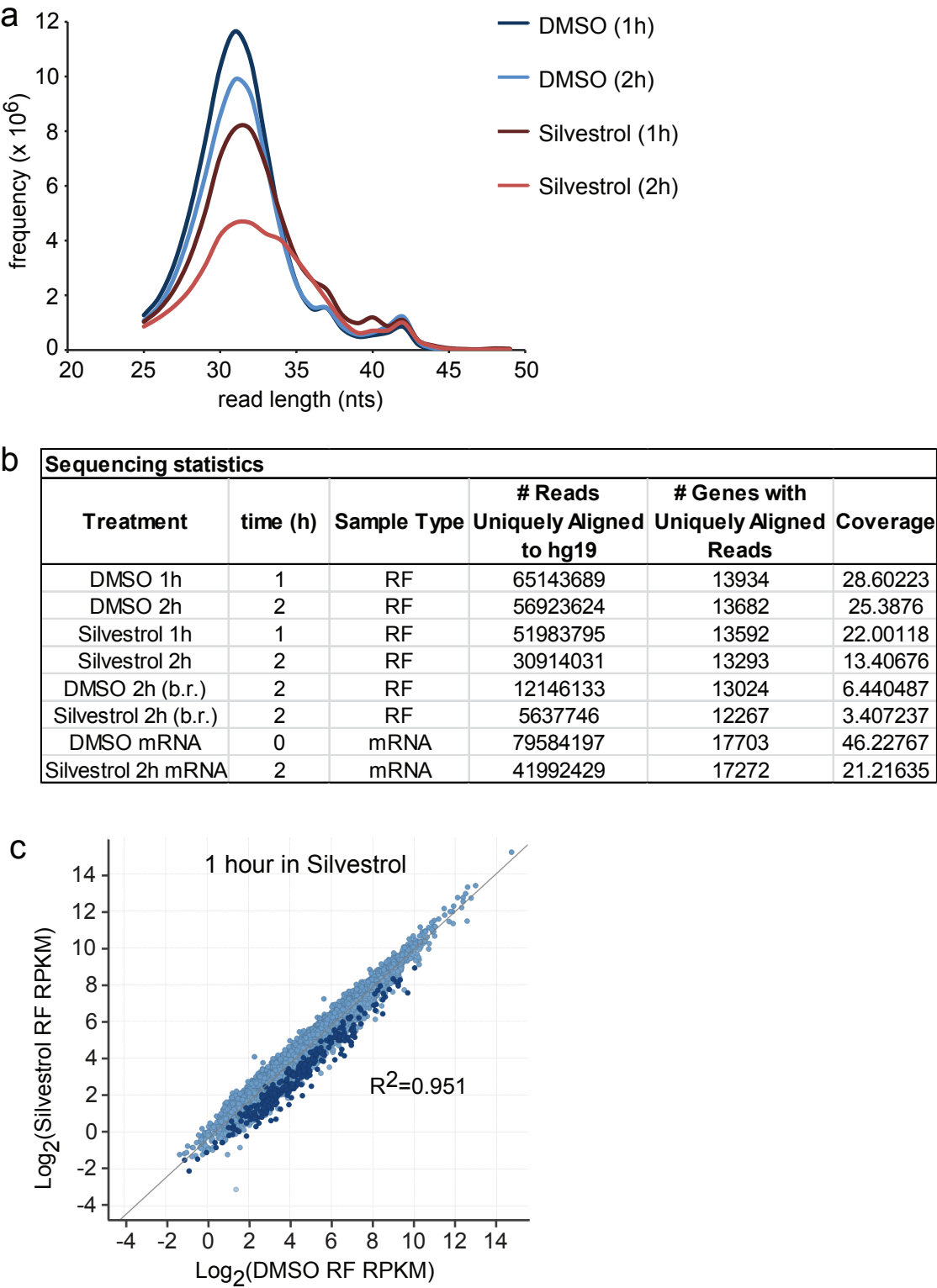

Supplement: Additional file 3: Figure S2. — Select data from ribosome profiling sequencing. (a) The frequency of read lengths from RNA-Seq libraries generated by ribosome footprinting (RF). (b) Table of sequencing statistics for sample generated by next-generation sequencing. Coverage was computed using the Picard HsMetrics tool for every exon in the KnownGene database. Exons with fewer than two reads were excluded. Abbreviations: br, biological replicate; RF, ribosome footprint. (c) Scatter plot of RF densities (measured in RPKM) in MDA-MB-231 cells treated with 25 nM Silvestrol versus DMSO for 1 hour. Silvestrol-sensitive genes are indicated in dark blue. [file 13059_2014_476_MOESM3_ESM.pdf]

Figure S3

DMSO  
Silvestrol

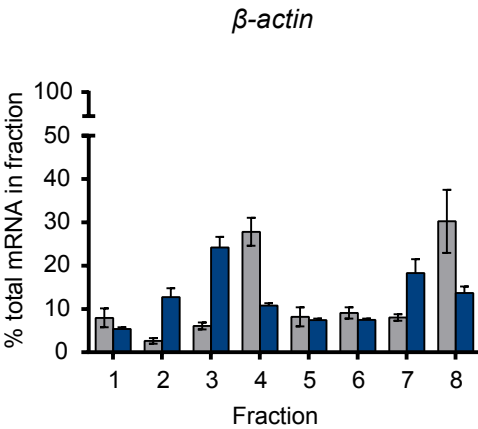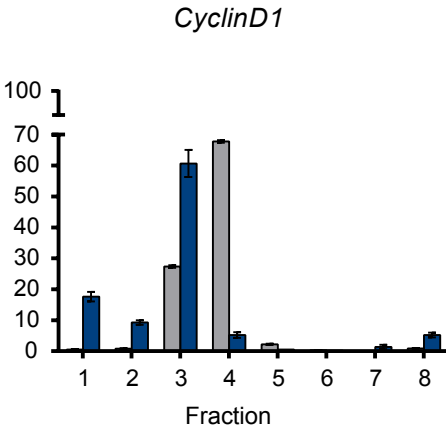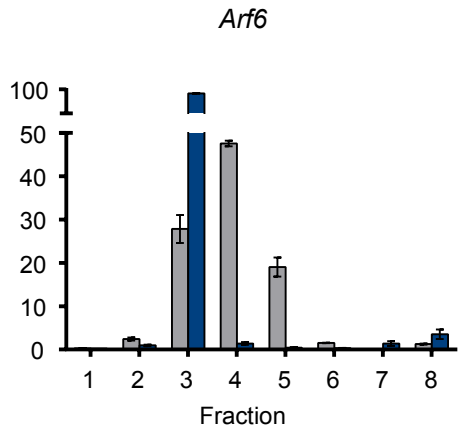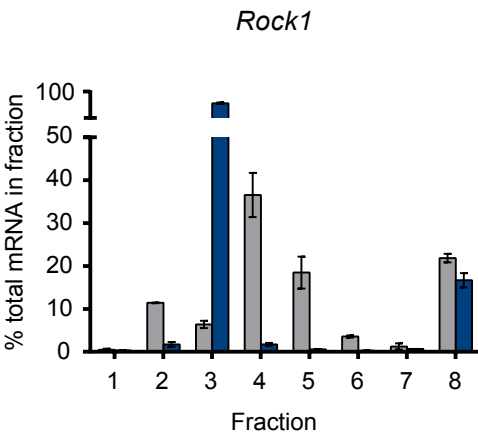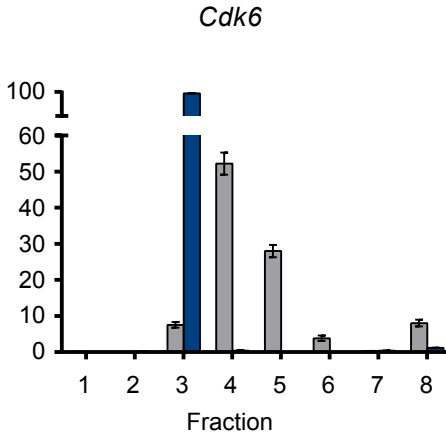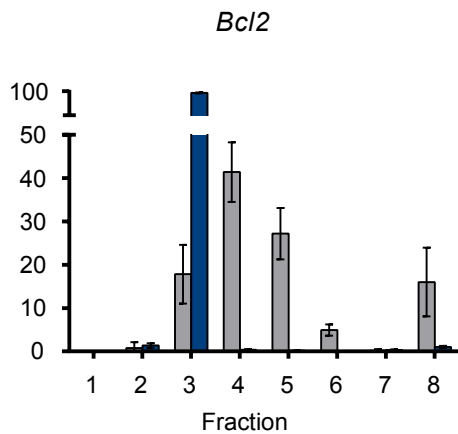

Supplement: Additional file 4: Figure S3. — Polysome analysis of select Silvestrol-sensitive transcripts. Polysomes from 25 nM Silvestrol- or DMSO-treated cells were fractionated and analyzed for the abundance of transcripts for β-actin, CyclinD1, ARF6, ROCK1, CDK6 and BCL2. Data presented are mean values ± standard error (n =2). [file 13059_2014_476_MOESM4_ESM.pdf]

Figure S4

a ● Decreased TE ● Insensitive ● Increased TE

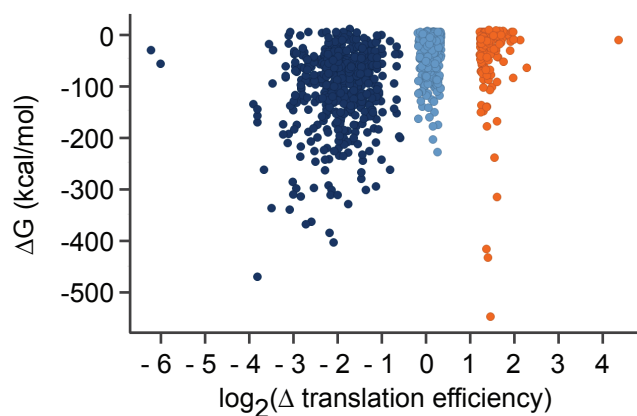

b

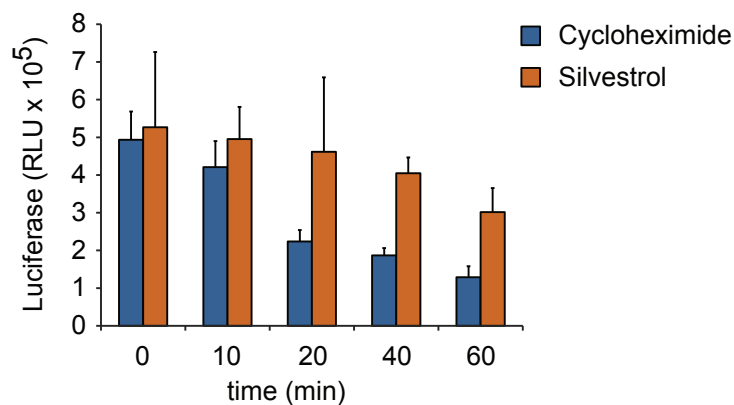

c

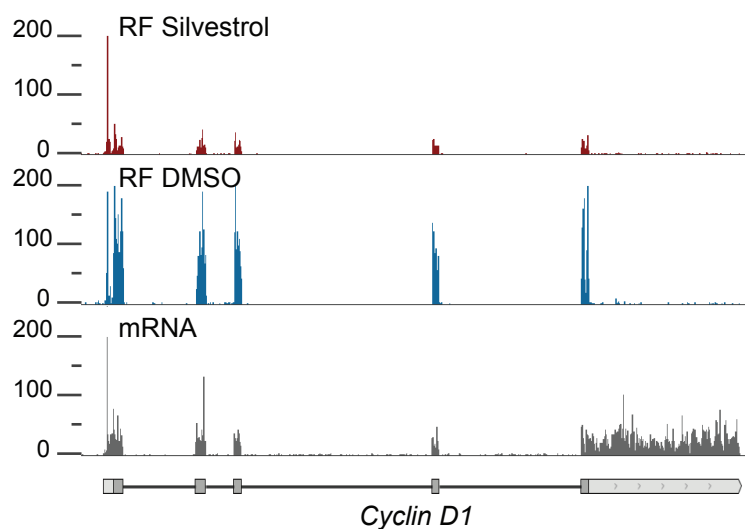

d

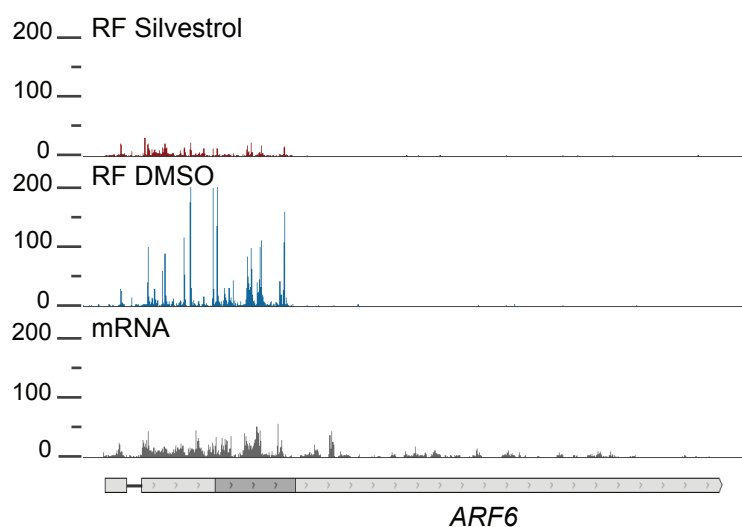

e

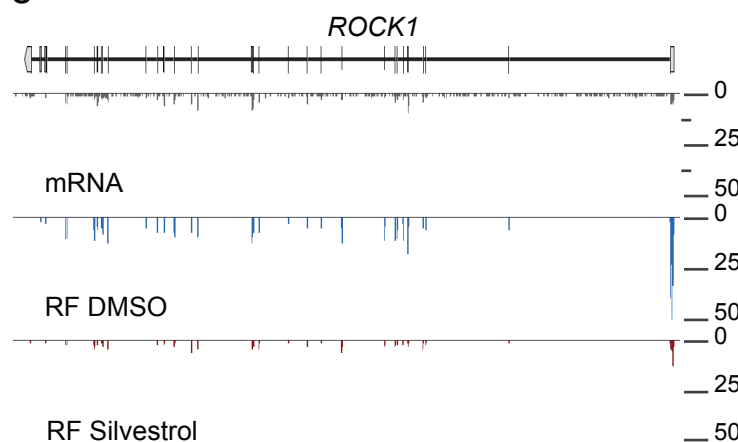

f

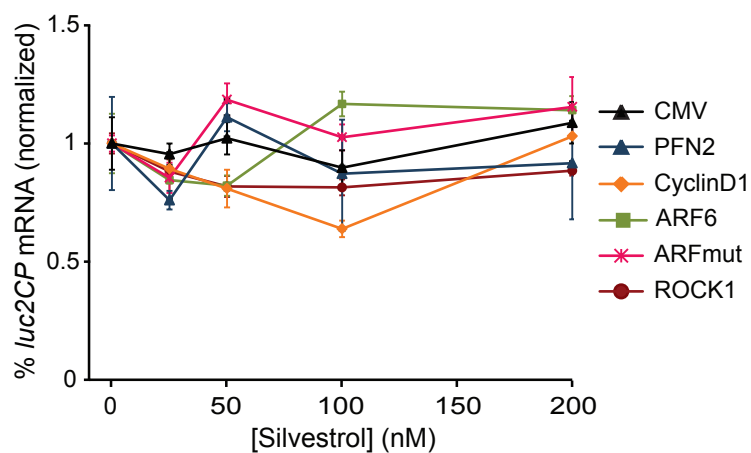

Supplement: Additional file 5: Figure S4. — Structured 5′ UTRs from Silvestrol-sensitive transcripts, used to evaluate 5′ UTR contribution to translation of a luciferase reporter. (a) Free energy values for the 5′ UTRs of Silvestrol-sensitive genes; genes are separated into three classes based on TE values: Decreased TE (n =284), insensitive (n =299) and increased TE (n =146). Energy values were predicted using the CONTRAfold algorithm. The pool of genes with decreased TE values are enriched with structured 5′ UTRs. (b) Luciferase production from CMV-driven luc2CP (pMH2) in the presence of cycloheximide (100 μg/ml) or Silvestrol (50 nM) over time. Half-life of Luc2CP protein is 30.99 minutes. In each experiment, samples were assayed in triplicate; data were derived from two independent experiments. (c-e) Plots of sequencing data derived from genes selected for 5′ UTR assessment. Data represent the non-normalized frequency of either the 5′end (mRNA) or P-site (RF) of each read found in sequencing data. (f) Luc2CP mRNA levels in Silvestrol-treated 293 T cells bearing the indicated reporter constructs. Luc2CP abundance was measured by quantitative PCR and normalized to β-actin mRNA levels. Data represent mean values ± standard error of the mean (n =3). [file 13059_2014_476_MOESM5_ESM.pdf]

Figure S5

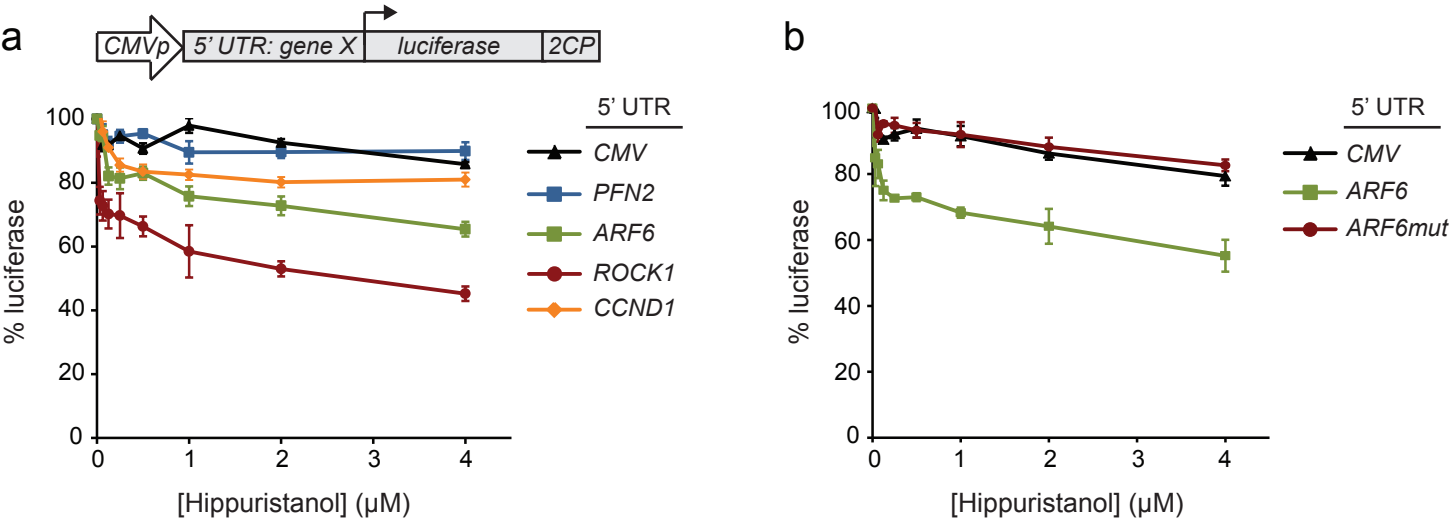

Supplement: Additional file 6: Figure S5. — Luciferase expression from 5′ UTR reporter constructs upon treatment with hippuristanol. (a) Luciferase reporter constructs, stably transfected into 293 T cells, were treated with increasing concentrations of hippuristanol and luciferase expression was measured after 40 minutes; constructs bearing 5′ UTRs from Silvestrol-sensitive genes (CyclinD1, ROCK1, ARF6) or insensitive genes (PFN2 or CMV alone) were compared. (b) Luciferase expression from reporter constructs bearing ARF6wt 5′ UTR or ARF6mut 5′ UTR, stably transfected into 293 T cells. Cells were treated with increasing concentrations of hippuristanol and measurements were taken after 40 minutes of exposure. Data presented were obtained from two independent experiments with measurements taken in triplicate. [file 13059_2014_476_MOESM6_ESM.pdf]

Figure S6

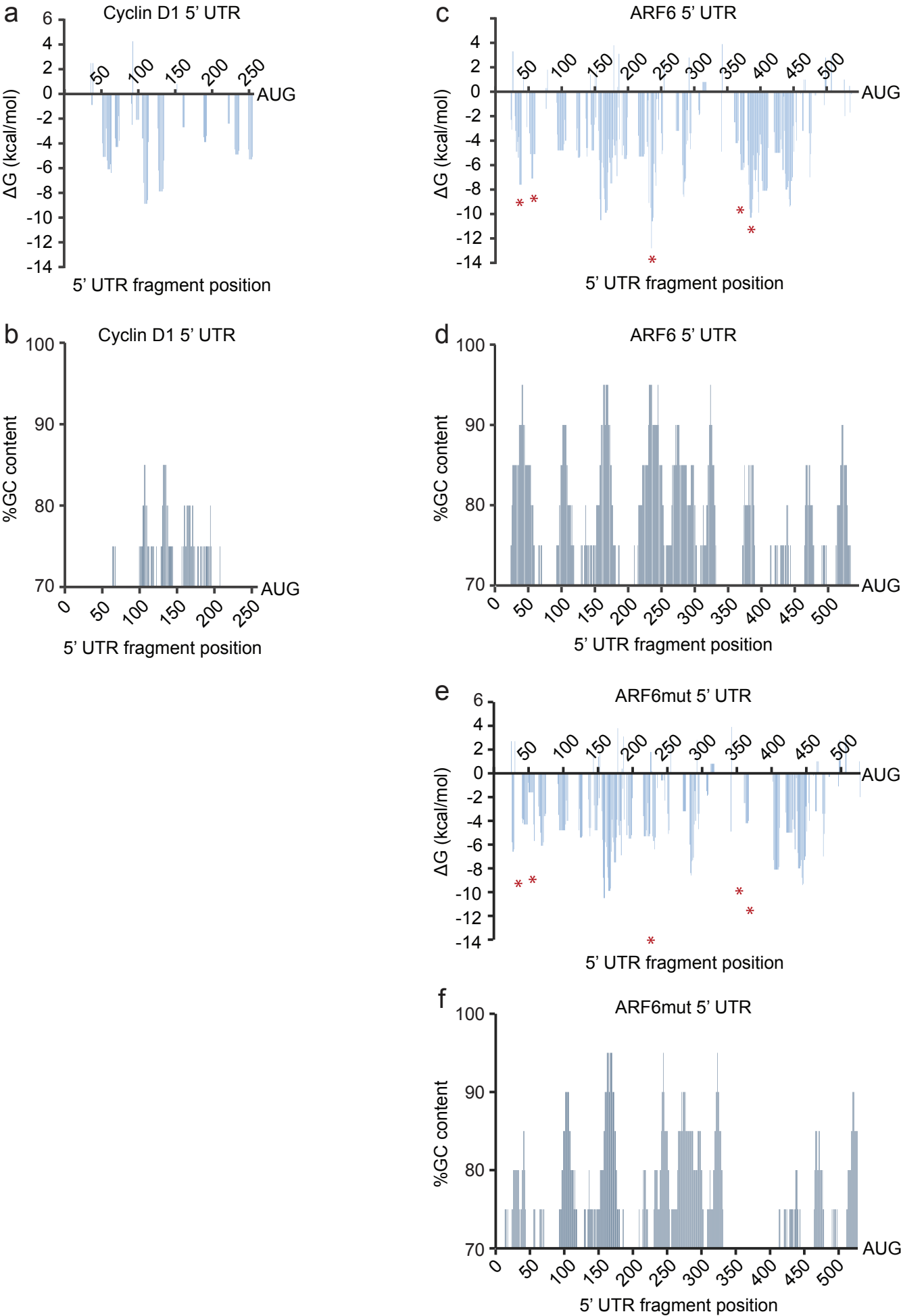

Supplement: Additional file 7: Figure S6. — Sliding window structure analysis of select 5′ UTRs. (a,b) Free energy values (a) and percentage GC content (b) of 20-nucleotide fragments of the 5′ UTR of CyclinD1 plotted along the length of the UTR from the TSS to the translation start (AUG). (c-f) Free energy values (c,e) and percentage GC content (d,f) of the 5′ UTR of ARF6 (c,d) and ARF6mut (e,f) plotted along the length of the UTR from the TSS to the translation start (AUG). Each asterisk denotes a structured region that was mutated in ARF6 to create ARF6mut. [file 13059_2014_476_MOESM7_ESM.pdf]

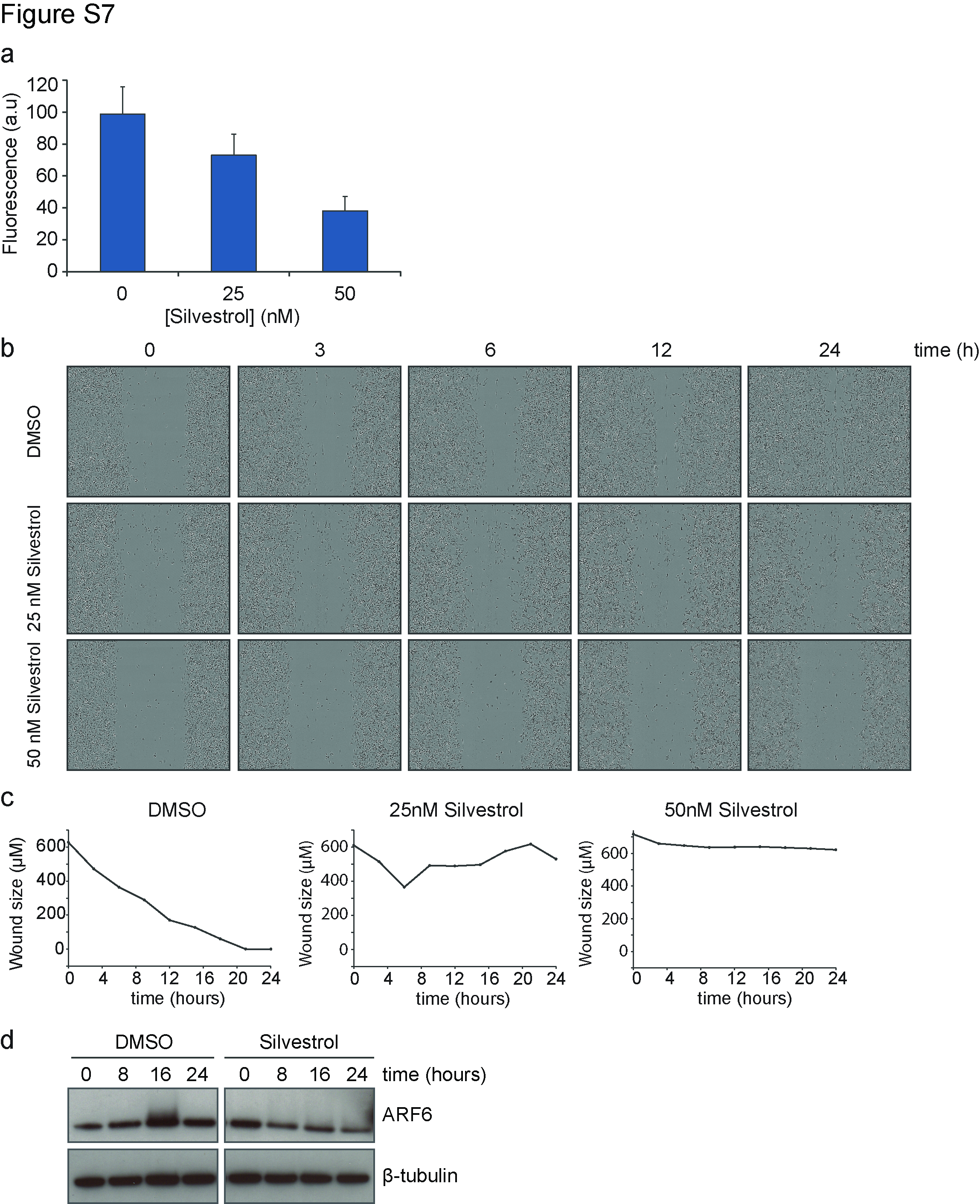

Supplement: Additional file 8: Figure S7. — Silvestrol treatment impairs migration of MDA-MB-231 cells. (a) Migration of MDA-MB-231 cells was measured in a trans-well migration assay in the presence of increasing amounts of Silvestrol. Plots represent Calcenein AM fluorescence of cells which migrated across a barrier. (b) MDA-MB-231 cells were grown to a confluent monolayer and scratched to produce a wound. Wound closure was monitored over time by microscopy. (c) Quantification of wound closure (represented in (b)) over time. (d) Western blot for Arf6 protein after treatment with 25 nM Silvestrol. [file 13059_2014_476_MOESM8_ESM.tiff]

Figure S8

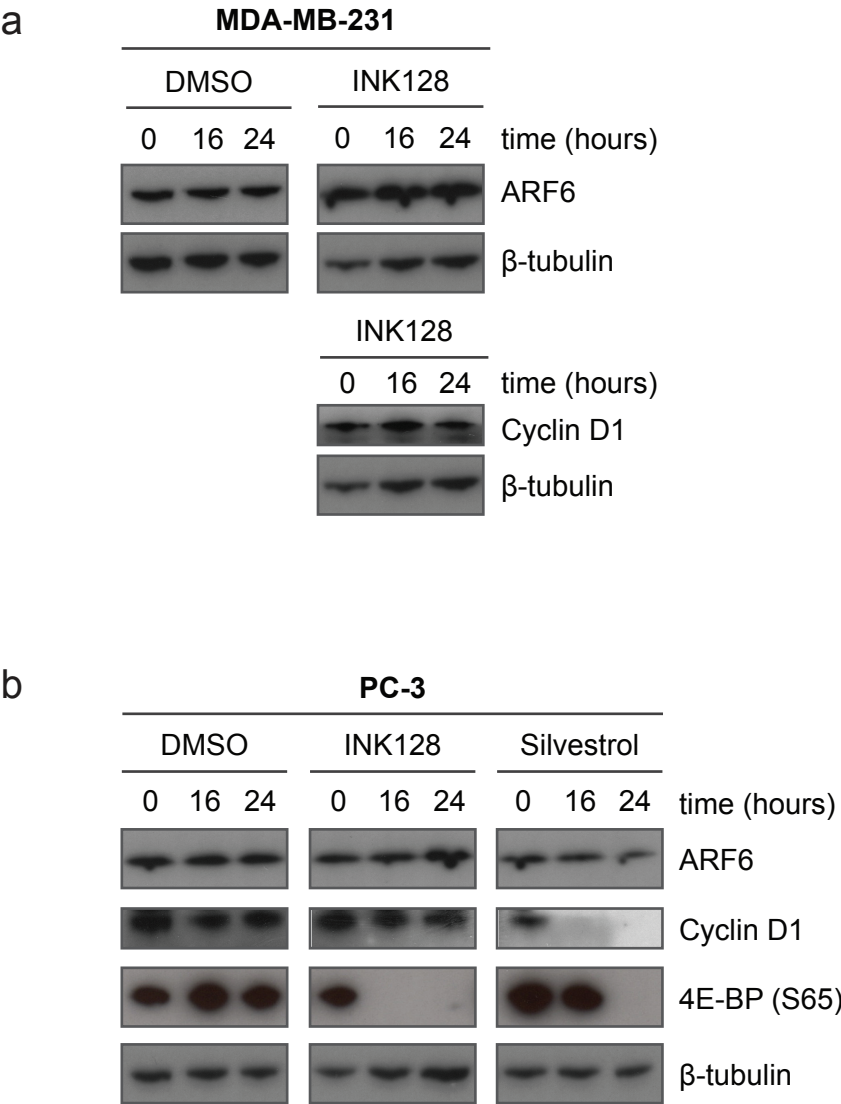

Supplement: Additional file 9: Figure S8. — Translation of Silvestrol-sensitive transcripts is not disrupted by mTOR inhibition. (a) MDA-MB-231 cells were treated with vehicle (DMSO) or 200 nM INK128 for the indicated times and Western blots were performed for Arf6 (top panels) and Cyclin D1 (bottom panels). (b) PC-3 cells were treated with vehicle (DMSO), 200 nM INK128 or 25 nM Silvestrol for the indicated times. Western blots were performed for Arf6, Cyclin D1 and phosphorylated 4E-BP1/2 (Ser65). [file 13059_2014_476_MOESM9_ESM.pdf]

Figure S9

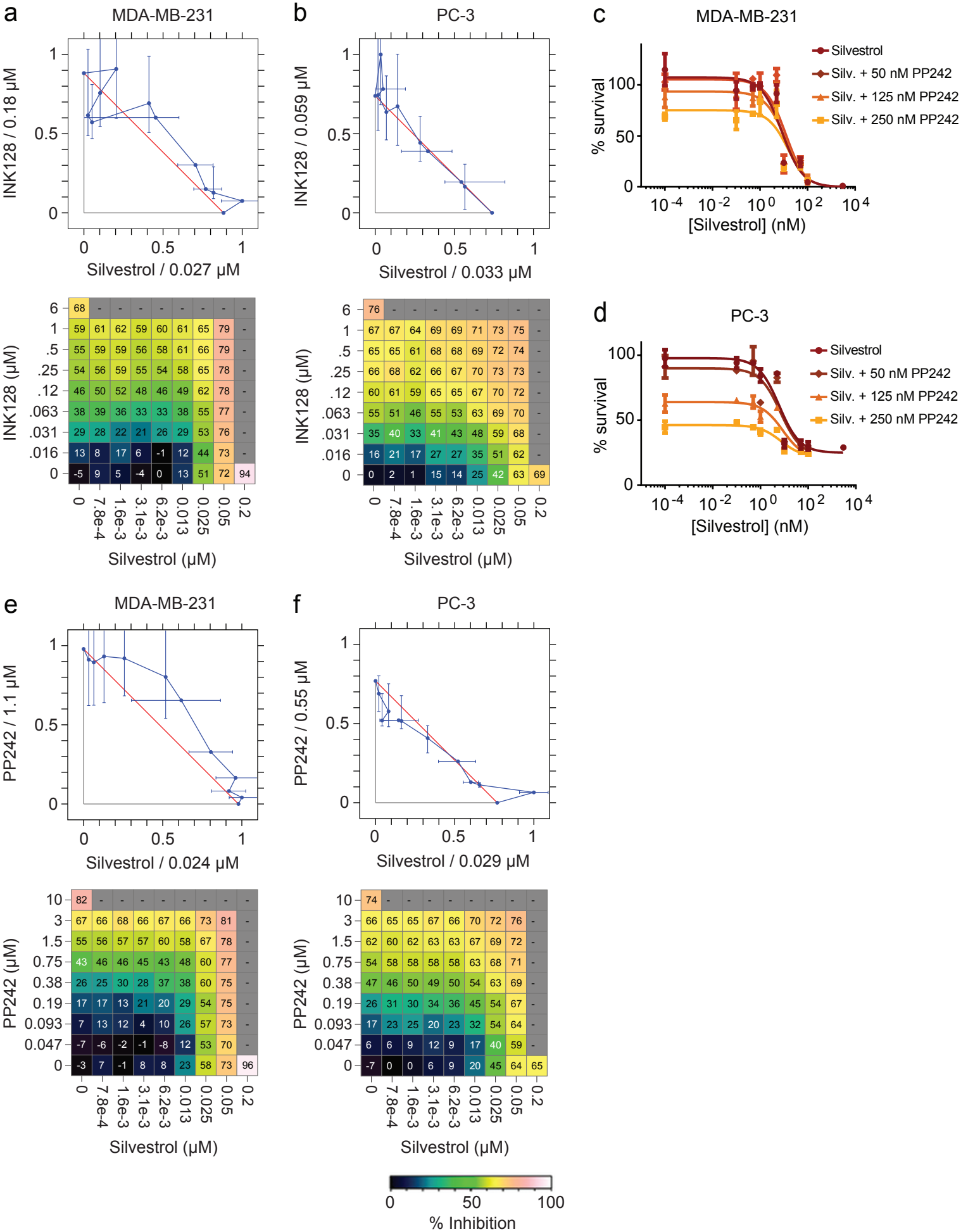

Supplement: Additional file 10: Figure S9. — Combinatorial treatment with Silvestrol and mTOR inhibitors show additivity in blocking cancer cell proliferation. (a,b) Isobolograms (top panels) and dose matrices (bottom panels) of MDA-MB-231 (a) and PC-3 (b) cells treated with serial combinations of Silvestrol and INK128. (c,d) MDA-MB-231 (c) or PC-3 (d) cells were treated with increasing concentrations of Silvestrol in combination with PP242 at fixed doses: 50 nM, 125 nM and 250 nM. Cell viability was measured by CellTiter-Glo after 3 days. (e,f) Isobolograms (top panels) and dose matrices (bottom panels) of MDA-MB-231 (e) and PC-3 (f) cells treated with serial combinations of Silvestrol and PP242. [file 13059_2014_476_MOESM10_ESM.pdf]
