# Supplementary material for: Transcriptome-wide characterization of the eIF4A signature highlights plasticity in translation regulation
Source: Genome Biol. 2014 Oct 2;15(10):476. doi: 10.1186/s13059-014-0476-1 (PMC4203936; doi:10.1186/s13059-014-0476-1)
Supplement: Additional file 2: Table S1. — Mass spectrometry of monosome fractions from MDA-MB-231 cells. LC-MS/MS mass spectrometry was performed using monosome fractions from (a) DMSO- or (b) Silvestrol-treated MDA-MB-231 cells. Proteins were identified by Mascot using peptide sequences as query; those with 98% confidence or greater are listed for each replicate sample. Table S2: Genes showing reduced TE in Silvestrol. Table of 284 genes identified by ribosome profiling as having reduced TE in the presence of 25 nM Silvestrol. Genes are organized based on fold change in TE (∆TE) values. Table S3: Genes showing increased TE in Silvestrol. Table of 146 genes identified by ribosome profiling as having increased TE in the presence of 25 nM Silvestrol. Genes are organized based on fold change in TE (∆TE) values. Table S4: 5′ UTR characteristics of Silvestrol-sensitive and -insensitive genes. Table showing free energy values (∆G), length and percentage GC content for 5′ UTRs from genes that are sensitive (decreased ∆TE) or insensitive to 25 nM Silvestrol. ∆G values were predicted using two algorithms, CONTRAfold or McCaskill [44]. The correlation coefficient for these data sets is r =0.914. Genes bearing variant 5′ UTR isoforms are indicated with an asterisk. Table S5: Table of vectors used in luciferase reporter assays. Vector pMH2 was constructed from pGL4.25 with CMV promoter in place of the minimal promoter. Vectors pCR300, pCR301, pCR302, pCR303, and pCR304 were constructed by adding the indicated 5′ UTRs to vector pMH2. See Materials and methods for cloning information. Table S6: Genes showing reduced TE in INK128. Table of 244 genes that showed reduced TE in MDA-MB-231 cells treated with 200 nM INK128 for 2 hours. The gene also found to have reduced TE after Silvestrol treatment is indicated by asterisks. [file 13059_2014_476_MOESM2_ESM.pdf]

Table S1A						
**Peptides discovered by mass spectrometry of monosome pellet | DMSO**						
Rank	Protein	Description	Score	Mass (Da)	No. of peptides	% Coverage
1	RSSA	40S ribosomal protein SA (p40) (34/67 kDa laminin receptor) (Colon carcinoma laminin-binding protein) (NEM/1CHD4) (Multidrug resistance-associated protein MGr1-Ag) - Homo sapiens (Human)	752	32833	82	45.4
2	RL4	60S ribosomal protein L4 (L1) - Homo sapiens (Human)	651	47667	128	43.8
3	RS16	40S ribosomal protein S16 - Homo sapiens (Human)	626	16435	105	63
4	RS8	40S ribosomal protein S8 - Homo sapiens (Human)	561	24190	57	56.7
5	RS19	40S ribosomal protein S19 - Homo sapiens (Human)	517	16051	89	50.3
6	RS7	40S ribosomal protein S7 - Homo sapiens (Human)	515	22113	90	66
7	RL7	60S ribosomal protein L7 - Homo sapiens (Human)	514	29207	71	56.5
8	RS3	40S ribosomal protein S3 - Homo sapiens (Human)	511	26671	120	77.8
9	RS4X	40S ribosomal protein S4, X isoform (Single copy abundant mRNA protein) (SCR10) - Homo sapiens (Human)	468	29579	92	46
10	RL7A	60S ribosomal protein L7a (Surfeit locus protein 3) (PLA-X polypeptide) - Homo sapiens (Human)	464	29977	88	43.6
11	RS5	40S ribosomal protein S5 - Homo sapiens (Human)	459	22862	82	50
12	ACTB	Actin, cytoplasmic 1 (Beta-actin) - Homo sapiens (Human)	444	41710	91	52
13	RL22	60S ribosomal protein L22 (Epstein-Barr virus small RNA-associated protein) (EBER-associated protein) (EAP) (Heparin-binding protein HBp15) - Homo sapiens (Human)	435	14778	57	68.8
14	RL5	60S ribosomal protein L5 - Homo sapiens (Human)	423	34341	80	42.1

15	RS21	40S ribosomal protein S21 - Homo sapiens (Human)	421	9106	25	34.9
16	ACTA	Actin, aortic smooth muscle (Alpha-actin-2) - Homo sapiens (Human)	398	41982	68	28.6
17	RLA0	60S acidic ribosomal protein P0 (L10E) - Homo sapiens (Human)	395	34252	81	49.2
18	RL10	60S ribosomal protein L10 (QM protein) (Tumor suppressor QM) (Laminin receptor homolog) - Homo sapiens (Human)	395	24561	88	61.7
19	RL12	60S ribosomal protein L12 - Homo sapiens (Human)	348	17808	51	40.6
20	RL15	60S ribosomal protein L15 - Homo sapiens (Human)	347	24131	54	33.8
21	RL30	60S ribosomal protein L30 - Homo sapiens (Human)	342	12776	64	72.2
22	RL3	60S ribosomal protein L3 (HIV-1 TAR RNA-binding protein B) (TARBP-B) - Homo sapiens (Human)	342	46080	86	42.9
23	RL13	60S ribosomal protein L13 (Breast basic conserved protein 1) - Homo sapiens (Human)	330	24247	70	32.2
24	TBB5	Tubulin beta chain (Tubulin beta-5 chain) - Homo sapiens (Human)	319	49639	89	39.2
25	RS18	40S ribosomal protein S18 (Ke-3) (Ke3) - Homo sapiens (Human)	317	17708	67	46.1
26	RS23	40S ribosomal protein S23 - Homo sapiens (Human)	315	15798	48	51
27	MYH9	Myosin-9 (Myosin heavy chain 9) (Myosin heavy chain, nonmuscle IIa) (Nonmuscle myosin heavy chain IIa) (NMMHC II-a) (NMMHC-IIA) (Cellular myosin heavy chain, type A) (Nonmuscle myosin heavy chain-A) (NMMHC-A) - Homo sapiens (Human)	293	226392	108	24
28	RS2	40S ribosomal protein S2 (S4) (LLRep3 protein) - Homo sapiens (Human)	273	31305	66	35.2

29	RS3A	40S ribosomal protein S3a - Homo sapiens (Human)	270	29926	74	46.6
30	RL6	60S ribosomal protein L6 (TAX-responsive enhancer element-binding protein 107) (TAXREB107) (Neoplasm-related protein C140) - Homo sapiens (Human)	261	32708	68	33
31	TBB2A	Tubulin beta-2A chain - Homo sapiens (Human)	256	49875	52	29.7
32	RL11	60S ribosomal protein L11 (CLL-associated antigen KW-12) - Homo sapiens (Human)	254	20240	53	27.5
33	RLA1	60S acidic ribosomal protein P1 - Homo sapiens (Human)	248	11507	28	51.8
34	RS13	40S ribosomal protein S13 - Homo sapiens (Human)	245	17212	73	62.3
35	TBB2C	Tubulin beta-2C chain (Tubulin beta-2 chain) - Homo sapiens (Human)	234	49799	53	32.4
36	RL24	60S ribosomal protein L24 (Ribosomal protein L30) - Homo sapiens (Human)	229	17768	54	37.6
37	RL18	60S ribosomal protein L18 - Homo sapiens (Human)	229	21621	48	38.8
38	RL23	60S ribosomal protein L23 (Ribosomal protein L17) - Homo sapiens (Human)	217	14856	64	53.6
39	GBLP	Guanine nucleotide-binding protein subunit beta 2-like 1 (Guanine nucleotide-binding protein subunit beta-like protein 12.3) (Receptor of activated protein kinase C 1) (RACK1) (Receptor for activated C kinase) - Homo sapiens (Human)	214	35055	65	32.2
40	RL27A	60S ribosomal protein L27a - Homo sapiens (Human)	207	16551	45	46.6
41	RS15A	40S ribosomal protein S15a - Homo sapiens (Human)	205	14830	49	61.5
42	RL13A	60S ribosomal protein L13a (23 kDa highly basic protein) - Homo sapiens (Human)	201	23562	51	31.5

43	RS14	40S ribosomal protein S14 - Homo sapiens (Human)	197	16263	39	41.7
44	RL18A	60S ribosomal protein L18a - Homo sapiens (Human)	197	20749	62	47.2
45	TCPG	T-complex protein 1 subunit gamma (TCP-1-gamma) (CCT-gamma) (hTRiC5) - Homo sapiens (Human)	194	60495	50	30.1
46	RL31	60S ribosomal protein L31 - Homo sapiens (Human)	192	14454	29	52.8
47	ALBU	Serum albumin precursor - Homo sapiens (Human)	187	69321	35	8.7
48	RS25	40S ribosomal protein S25 - Homo sapiens (Human)	186	13734	43	32
49	TCPB	T-complex protein 1 subunit beta (TCP-1-beta) (CCT-beta) - Homo sapiens (Human)	166	57452	16	23
50	RL19	60S ribosomal protein L19 - Homo sapiens (Human)	163	23451	30	21.9
51	RLA2	60S acidic ribosomal protein P2 (Renal carcinoma antigen NY-REN-44) - Homo sapiens (Human)	162	11658	32	52.2
52	TCPH	T-complex protein 1 subunit eta (TCP-1-eta) (CCT-eta) (HIV-1 Nef-interacting protein) - Homo sapiens (Human)	160	59329	48	28
53	RL32	60S ribosomal protein L32 - Homo sapiens (Human)	157	15850	41	57.8
54	RL8	60S ribosomal protein L8 - Homo sapiens (Human)	152	28007	39	37.4
55	TCPA	T-complex protein 1 subunit alpha (TCP-1-alpha) (CCT-alpha) - Homo sapiens (Human)	135	60306	22	24.3
56	TCPQ	T-complex protein 1 subunit theta (TCP-1-theta) (CCT-theta) (Renal carcinoma antigen NY-REN-15) - Homo sapiens (Human)	135	59583	27	32.7
57	RL36	60S ribosomal protein L36 - Homo sapiens (Human)	123	12246	31	37.1
58	RS20	40S ribosomal protein S20 - Homo sapiens (Human)	106	13364	44	48.7

59	RL14	60S ribosomal protein L14 (CAG-ISL 7) - Homo sapiens (Human)	100	23275	47	34.3
60	RL10A	60S ribosomal protein L10a (CSA-19) - Homo sapiens (Human)	99	24816	33	35.5
61	RL35	60S ribosomal protein L35 - Homo sapiens (Human)	98	14543	16	29.3
62	MYO1C	Myosin-Ic (Myosin I beta) (MMI-beta) (MMIb) - Homo sapiens (Human)	95	117965	21	11.7
63	RS11	40S ribosomal protein S11 - Homo sapiens (Human)	94	18419	55	50
64	TCPE	T-complex protein 1 subunit epsilon (TCP-1-epsilon) (CCT-epsilon) - Homo sapiens (Human)	93	59633	33	24.8
65	RS26	40S ribosomal protein S26 - Homo sapiens (Human)	87	13007	28	33.9
66	RS24	40S ribosomal protein S24 - Homo sapiens (Human)	86	15413	27	36.1
67	RS17	40S ribosomal protein S17 - Homo sapiens (Human)	85	15540	50	63
68	RL29	60S ribosomal protein L29 (Cell surface heparin-binding protein HIP) - Homo sapiens (Human)	79	17741	13	21.4
69	RS27L	40S ribosomal protein S27-like protein - Homo sapiens (Human)	75	9471	22	27.4
70	RL17	60S ribosomal protein L17 (L23) - Homo sapiens (Human)	75	21383	50	44
71	RL26	60S ribosomal protein L26 - Homo sapiens (Human)	73	17248	34	40.7
72	RL28	60S ribosomal protein L28 - Homo sapiens (Human)	72	15738	23	37.2
73	RS29	40S ribosomal protein S29 - Homo sapiens (Human)	71	6672	22	46.4
74	RL27	60S ribosomal protein L27 - Homo sapiens (Human)	71	15788	14	47.8
75	RL9	60S ribosomal protein L9 - Homo sapiens (Human)	69	21850	45	43.2
76	MLRM	Myosin regulatory light chain 2, nonsarcomeric (Myosin RLC) - Homo sapiens (Human)	62	19781	6	5.8

77	HSP7C	Heat shock cognate 71 kDa protein (Heat shock 70 kDa protein 8) - Homo sapiens (Human)	60	70854	18	11.8
78	RL23A	60S ribosomal protein L23a - Homo sapiens (Human)	59	17684	33	32.7
79	RL21	60S ribosomal protein L21 - Homo sapiens (Human)	57	18553	39	35.6
80	RS6	40S ribosomal protein S6 (Phosphoprotein NP33) - Homo sapiens (Human)	56	28663	26	34.1
81	UBIQ	Ubiquitin - Homo sapiens (Human)	51	8560	16	39.5
82	RS12	40S ribosomal protein S12 - Homo sapiens (Human)	50	14516	13	17.4
83	RS9	40S ribosomal protein S9 - Homo sapiens (Human)	50	22578	37	36.1
84	RL40	60S ribosomal protein L40 (CEP52) - Homo sapiens (Human)	50	6177	8	13.5
85	RS30	40S ribosomal protein S30 - Homo sapiens (Human)	47	6644	14	18.6
86	RS10	40S ribosomal protein S10 - Homo sapiens (Human)	45	18886	7	10.9
87	MYL6	Myosin light polypeptide 6 (Smooth muscle and nonmuscle myosin light chain alkali 6) (Myosin light chain alkali 3) (Myosin light chain 3) (MLC-3) (LC17) - Homo sapiens (Human)	45	16919	25	35.1
88	RL38	60S ribosomal protein L38 - Homo sapiens (Human)	41	8213	13	34.3
89	TCPZ	T-complex protein 1 subunit zeta (TCP-1-zeta) (CCT-zeta) (CCT-zeta-1) (Tcp20) (HTR3) (Acute morphine dependence-related protein 2) - Homo sapiens (Human)	41	57988	33	30.9
90	TBAK	Tubulin alpha-ubiquitous chain (Alpha-tubulin ubiquitous) (Tubulin K-alpha-1) - Homo sapiens (Human)	37	50120	35	20.4
91	RL34	60S ribosomal protein L34 - Homo sapiens (Human)	36	13284	28	21.4

92	DYHC	Dynein heavy chain, cytosolic (DYHC) (Cytoplasmic dynein heavy chain 1) (DHC1) (Dynein heavy chain 1, cytoplasmic 1) - Homo sapiens (Human)	36	532072	7	0.9
93	SYEP	Bifunctional aminoacyl-tRNA synthetase [Includes: Glutamyl-tRNA synthetase (EC 6.1.1.17) (Glutamate--tRNA ligase); Prolyl-tRNA synthetase (EC 6.1.1.15) (Proline--tRNA ligase)] - Homo sapiens (Human)	34	162923	6	6.3
94	RL35A	60S ribosomal protein L35a - Homo sapiens (Human)	33	12530	22	21.8
95	MVP	Major vault protein (MVP) (Lung resistance-related protein) - Homo sapiens (Human)	33	99266	25	24.1
96	SMN	Survival motor neuron protein (Component of gems 1) (Gemin-1) - Homo sapiens (Human)	33	31828	2	6.1
97	TCPD	T-complex protein 1 subunit delta (TCP-1-delta) (CCT-delta) (Stimulator of TAR RNA-binding) - Homo sapiens (Human)	32	57888	36	46.4
98	PSB1	Proteasome subunit beta type 1 precursor (EC 3.4.25.1) (Proteasome component C5) (Macropain subunit C5) (Multicatalytic endopeptidase complex subunit C5) (Proteasome gamma chain) - Homo sapiens (Human)	30	26472	6	7.5
99	TPP2	Tripeptidyl-peptidase 2 (EC 3.4.14.10) (Tripeptidyl-peptidase II) (TPP-II) (Tripeptidyl aminopeptidase) - Homo sapiens (Human)	29	138263	1	1
100	ATPB	ATP synthase subunit beta, mitochondrial precursor (EC 3.6.3.14) - Homo sapiens (Human)	28	56525	9	12.5

101	PSA4	Proteasome subunit alpha type 4 (EC 3.4.25.1) (Proteasome component C9) (Macropain subunit C9) (Multicatalytic endopeptidase complex subunit C9) (Proteasome subunit L) - Homo sapiens (Human)	27	29465	2	6.5
102	IGHG1	Ig gamma-1 chain C region - Homo sapiens (Human)	27	36083	8	3.6
103	RS28	40S ribosomal protein S28 - Homo sapiens (Human)	27	7836	6	40.6
104	RS15	40S ribosomal protein S15 (RIG protein) - Homo sapiens (Human)	25	17029	25	42.8
105	CLH1	Clathrin heavy chain 1 (CLH-17) - Homo sapiens (Human)	23	191493	1	1.1
106	PYR1	CAD protein [Includes: Glutamine-dependent carbamoyl-phosphate synthase (EC 6.3.5.5); Aspartate carbamoyltransferase (EC 2.1.3.2); Dihydroorotase (EC 3.5.2.3)] - Homo sapiens (Human)	23	242829	1	0.5
107	EF1A1	Elongation factor 1-alpha 1 (EF-1-alpha-1) (Elongation factor 1 A-1) (eEF1A-1) (Elongation factor Tu) (EF-Tu) (Leukocyte receptor cluster member 7) - Homo sapiens (Human)	23	50109	5	8
108	BAG2	BAG family molecular chaperone regulator 2 (BCL2-associated athanogene 2) (BAG-2) - Homo sapiens (Human)	23	23757	5	13.3
109	RL39	60S ribosomal protein L39 - Homo sapiens (Human)	23	6403	9	23.5

110	TCPW	T-complex protein 1 subunit zeta-2 (TCP-1-zeta-2) (CCT-zeta-2) (TCP-1-zeta-like) (CCT-zeta-like) (Testis-specific Tcp20) (Testis-specific protein TSA303) - Homo sapiens (Human)	22	57729	10	6.2
111	SYDC	Aspartyl-tRNA synthetase, cytoplasmic (EC 6.1.1.12) (Aspartate--tRNA ligase) (AspRS) (Cell proliferation-inducing gene 40 protein) - Homo sapiens (Human)	22	57100	6	8.2
112	MCE1	mRNA capping enzyme (HCE) (HCAP1) [Includes: Polynucleotide 5~-triphosphatase (EC 3.1.3.33) (mRNA 5~-triphosphatase) (TPase); mRNA guanylyltransferase (EC 2.7.7.50) (GTP--RNA guanylyltransferase) (GTase)] - Homo sapiens (Human)	21	68513	9	2.3
113	PSMD6	26S proteasome non-ATPase regulatory subunit 6 (26S proteasome regulatory subunit S10) (p42A) (Proteasome regulatory particle subunit p44S10) (Phosphonoformate immuno-associated protein 4) (Breast cancer-associated protein SGA-113M) - Homo sapiens (	21	45502	2	4.4
114	CH60	60 kDa heat shock protein, mitochondrial precursor (Hsp60) (60 kDa chaperonin) (CPN60) (Heat shock protein 60) (HSP-60) (Mitochondrial matrix protein P1) (P60 lymphocyte protein) (HuCHA60) - Homo sapiens (Human)	20	61016	11	4.9

115	PRS10	26S protease regulatory subunit S10B (Proteasome 26S subunit ATPase 6) (Proteasome subunit p42) - Homo sapiens (Human)	20	44145	1	3.1
116	PYGM	Glycogen phosphorylase, muscle form (EC 2.4.1.1) (Myophosphorylase) - Homo sapiens (Human)	20	97031	2	2
117	MCA1	Multisynthetase complex auxiliary component p43 [Contains: Endothelial monocyte-activating polypeptide 2 (EMAP-II) (Small inducible cytokine subfamily E member 1)] - Homo sapiens (Human)	20	34331	7	9.6
118	PSMD2	26S proteasome non-ATPase regulatory subunit 2 (26S proteasome regulatory subunit RPN1) (26S proteasome regulatory subunit S2) (26S proteasome subunit p97) (Tumor necrosis factor type 1 receptor-associated protein 2) (55.11 protein) - Homo sapiens (	19	100136	1	1.7
119	RUXE	Small nuclear ribonucleoprotein E (snRNP-E) (Sm protein E) (Sm-E) (SmE) - Homo sapiens (Human)	19	10797	5	12
120	RL36A	60S ribosomal protein L36a (60S ribosomal protein L44) (Migration-inducing gene 6 protein) - Homo sapiens (Human)	19	12433	6	6.6

Table S1B						
**Peptides discovered by mass spectrometry of monosome pellet | Silvestrol**						
Rank	Protein	Description	Score	Mass (Da)	No. of peptides	% Coverage
1	RSSA	40S ribosomal protein SA (p40) (34/67 kDa laminin receptor) (Colon carcinoma laminin-binding protein) (NEM/1CHD4) (Multidrug resistance-associated protein MGr1-Ag) - Homo sapiens (Human)	969	32833	102	51.9
2	MYH9	Myosin-9 (Myosin heavy chain 9) (Myosin heavy chain, nonmuscle IIa) (Nonmuscle myosin heavy chain IIa) (NMMHC II-a) (NMMHC-IIA) (Cellular myosin heavy chain, type A) (Nonmuscle myosin heavy chain-A) (NMMHC-A) - Homo sapiens (Human)	918	226392	216	31.1
3	RL7	60S ribosomal protein L7 - Homo sapiens (Human)	786	29207	127	64.5
4	RS16	40S ribosomal protein S16 - Homo sapiens (Human)	771	16435	116	66.4
5	RS3	40S ribosomal protein S3 - Homo sapiens (Human)	701	26671	153	77.8
6	RLA0	60S acidic ribosomal protein P0 (L10E) - Homo sapiens (Human)	674	34252	117	51.1
7	RL4	60S ribosomal protein L4 (L1) - Homo sapiens (Human)	659	47667	149	48.5
8	RS5	40S ribosomal protein S5 - Homo sapiens (Human)	640	22862	104	50
9	RL12	60S ribosomal protein L12 - Homo sapiens (Human)	597	17808	73	40.6
10	RS4X	40S ribosomal protein S4, X isoform (Single copy abundant mRNA protein) (SCR10) - Homo sapiens (Human)	576	29579	107	43.7
11	RS7	40S ribosomal protein S7 - Homo sapiens (Human)	551	22113	95	61.9
12	RS19	40S ribosomal protein S19 - Homo sapiens (Human)	533	16051	96	60.7

13	RL3	60S ribosomal protein L3 (HIV-1 TAR RNA-binding protein B) (TARBP-B) - Homo sapiens (Human)	523	46080	106	44.9
14	RL22	60S ribosomal protein L22 (Epstein-Barr virus small RNA-associated protein) (EBER-associated protein) (EAP) (Heparin-binding protein HBp15) - Homo sapiens (Human)	517	14778	73	71.9
15	ACTB	Actin, cytoplasmic 1 (Beta-actin) - Homo sapiens (Human)	496	41710	118	63.5
16	RS8	40S ribosomal protein S8 - Homo sapiens (Human)	494	24190	75	57.2
17	RL30	60S ribosomal protein L30 - Homo sapiens (Human)	475	12776	86	72.2
18	RS2	40S ribosomal protein S2 (S4) (LLRep3 protein) - Homo sapiens (Human)	463	31305	104	41.6
19	TBB5	Tubulin beta chain (Tubulin beta-5 chain) - Homo sapiens (Human)	462	49639	83	39.9
20	RL7A	60S ribosomal protein L7a (Surfeit locus protein 3) (PLA-X polypeptide) - Homo sapiens (Human)	449	29977	88	44.4
21	RL24	60S ribosomal protein L24 (Ribosomal protein L30) - Homo sapiens (Human)	444	17768	60	37.6
22	RL10	60S ribosomal protein L10 (QM protein) (Tumor suppressor QM) (Laminin receptor homolog) - Homo sapiens (Human)	441	24561	99	64.5
23	RL5	60S ribosomal protein L5 - Homo sapiens (Human)	437	34341	98	45.5
24	RL23	60S ribosomal protein L23 (Ribosomal protein L17) - Homo sapiens (Human)	435	14856	75	53.6
25	RS21	40S ribosomal protein S21 - Homo sapiens (Human)	418	9106	33	60.2
26	RS18	40S ribosomal protein S18 (Ke-3) (Ke3) - Homo sapiens (Human)	382	17708	78	45.4
27	RS17	40S ribosomal protein S17 - Homo sapiens (Human)	367	15540	71	69.6

28	RL13	60S ribosomal protein L13 (Breast basic conserved protein 1) - Homo sapiens (Human)	361	24247	81	39.3
29	RS13	40S ribosomal protein S13 - Homo sapiens (Human)	357	17212	90	58.3
30	GBLP	Guanine nucleotide-binding protein subunit beta 2-like 1 (Guanine nucleotide-binding protein subunit beta-like protein 12.3) (Receptor of activated protein kinase C 1) (RACK1) (Receptor for activated C kinase) - Homo sapiens (Human)	353	35055	86	48.6
31	ACTA	Actin, aortic smooth muscle (Alpha-actin-2) - Homo sapiens (Human)	351	41982	77	31.3
32	RL11	60S ribosomal protein L11 (CLL-associated antigen KW-12) - Homo sapiens (Human)	331	20240	54	27.5
33	RL27A	60S ribosomal protein L27a - Homo sapiens (Human)	326	16551	53	46.6
34	RL18	60S ribosomal protein L18 - Homo sapiens (Human)	320	21621	60	41
35	TBB3	Tubulin beta-3 chain (Tubulin beta-III) (Tubulin beta-4) - Homo sapiens (Human)	298	50400	33	20.4
36	TBB2C	Tubulin beta-2C chain (Tubulin beta-2 chain) - Homo sapiens (Human)	290	49799	48	35.7
37	RL6	60S ribosomal protein L6 (TAX-responsive enhancer element-binding protein 107) (TAXREB107) (Neoplasm-related protein C140) - Homo sapiens (Human)	285	32708	81	33
38	EF2	Elongation factor 2 (EF-2) - Homo sapiens (Human)	283	95277	72	32.5
39	RS20	40S ribosomal protein S20 - Homo sapiens (Human)	277	13364	41	63
40	RS23	40S ribosomal protein S23 - Homo sapiens (Human)	270	15798	56	54.5
41	RL15	60S ribosomal protein L15 - Homo sapiens (Human)	267	24131	70	39.7

42	RLA2	60S acidic ribosomal protein P2 (Renal carcinoma antigen NY-REN-44) - Homo sapiens (Human)	255	11658	38	52.2
43	RS3A	40S ribosomal protein S3a - Homo sapiens (Human)	251	29926	74	54.2
44	RLA1	60S acidic ribosomal protein P1 - Homo sapiens (Human)	244	11507	33	51.8
45	RS14	40S ribosomal protein S14 - Homo sapiens (Human)	240	16263	43	41.1
46	RL19	60S ribosomal protein L19 - Homo sapiens (Human)	233	23451	30	21.9
47	RL14	60S ribosomal protein L14 (CAG-ISL 7) - Homo sapiens (Human)	215	23275	55	39.9
48	RS15A	40S ribosomal protein S15a - Homo sapiens (Human)	209	14830	59	61.5
49	TCPH	T-complex protein 1 subunit eta (TCP-1-eta) (CCT-eta) (HIV-1 Nef-interacting protein) - Homo sapiens (Human)	208	59329	65	34.1
50	RL18A	60S ribosomal protein L18a - Homo sapiens (Human)	204	20749	86	58
51	RS25	40S ribosomal protein S25 - Homo sapiens (Human)	202	13734	50	32
52	RS24	40S ribosomal protein S24 - Homo sapiens (Human)	188	15413	34	40.6
53	RS11	40S ribosomal protein S11 - Homo sapiens (Human)	183	18419	73	63.3
54	RS15	40S ribosomal protein S15 (RIG protein) - Homo sapiens (Human)	173	17029	48	45.5
55	RL32	60S ribosomal protein L32 - Homo sapiens (Human)	160	15850	44	57.8
56	RL34	60S ribosomal protein L34 - Homo sapiens (Human)	159	13284	77	26.5
57	RL9	60S ribosomal protein L9 - Homo sapiens (Human)	158	21850	61	52.6
58	RL29	60S ribosomal protein L29 (Cell surface heparin-binding protein HIP) - Homo sapiens (Human)	153	17741	17	16.4
59	RL27	60S ribosomal protein L27 - Homo sapiens (Human)	146	15788	21	28.7

60	TCPE	T-complex protein 1 subunit epsilon (TCP-1-epsilon) (CCT-epsilon) - Homo sapiens (Human)	146	59633	34	30.7
61	RL31	60S ribosomal protein L31 - Homo sapiens (Human)	143	14454	37	52
62	MVP	Major vault protein (MVP) (Lung resistance-related protein) - Homo sapiens (Human)	143	99266	57	32.1
63	RS10	40S ribosomal protein S10 - Homo sapiens (Human)	139	18886	24	24.2
64	RL8	60S ribosomal protein L8 - Homo sapiens (Human)	138	28007	41	40.1
65	RS26	40S ribosomal protein S26 - Homo sapiens (Human)	134	13007	40	58.3
66	RL36	60S ribosomal protein L36 - Homo sapiens (Human)	134	12246	37	37.1
67	RL13A	60S ribosomal protein L13a (23 kDa highly basic protein) - Homo sapiens (Human)	123	23562	66	43.3
68	RL23A	60S ribosomal protein L23a - Homo sapiens (Human)	122	17684	56	33.3
69	RS27L	40S ribosomal protein S27-like protein - Homo sapiens (Human)	116	9471	25	27.4
70	TCPG	T-complex protein 1 subunit gamma (TCP-1-gamma) (CCT-gamma) (hTRiC5) - Homo sapiens (Human)	115	60495	44	32.7
71	RS12	40S ribosomal protein S12 - Homo sapiens (Human)	115	14516	17	20.5
72	TCPB	T-complex protein 1 subunit beta (TCP-1-beta) (CCT-beta) - Homo sapiens (Human)	109	57452	15	24.3
73	RL21	60S ribosomal protein L21 - Homo sapiens (Human)	102	18553	44	41.2
74	RL35	60S ribosomal protein L35 - Homo sapiens (Human)	95	14543	22	29.3
75	RL28	60S ribosomal protein L28 - Homo sapiens (Human)	92	15738	28	35
76	RL26	60S ribosomal protein L26 - Homo sapiens (Human)	92	17248	45	37.2

77	MYL6	Myosin light polypeptide 6 (Smooth muscle and nonmuscle myosin light chain alkali 6) (Myosin light chain alkali 3) (Myosin light chain 3) (MLC-3) (LC17) - Homo sapiens (Human)	91	16919	30	41.1
78	RS9	40S ribosomal protein S9 - Homo sapiens (Human)	90	22578	48	38.1
79	RS6	40S ribosomal protein S6 (Phosphoprotein NP33) - Homo sapiens (Human)	88	28663	34	34.1
80	RL17	60S ribosomal protein L17 (L23) - Homo sapiens (Human)	85	21383	59	43.5
81	MLRM	Myosin regulatory light chain 2, nonsarcomeric (Myosin RLC) - Homo sapiens (Human)	85	19781	7	5.8
82	TBAK	Tubulin alpha-ubiquitous chain (Alpha-tubulin ubiquitous) (Tubulin K-alpha-1) - Homo sapiens (Human)	84	50120	41	30.4
83	CH60	60 kDa heat shock protein, mitochondrial precursor (Hsp60) (60 kDa chaperonin) (CPN60) (Heat shock protein 60) (HSP-60) (Mitochondrial matrix protein P1) (P60 lymphocyte protein) (HuCHA60) - Homo sapiens (Human)	83	61016	14	4.9
84	RL10A	60S ribosomal protein L10a (CSA-19) - Homo sapiens (Human)	75	24816	45	44.7
85	UBIQ	Ubiquitin - Homo sapiens (Human)	73	8560	13	31.6
86	RS30	40S ribosomal protein S30 - Homo sapiens (Human)	73	6644	13	18.6
87	SMD1	Small nuclear ribonucleoprotein Sm D1 (snRNP core protein D1) (Sm-D1) (Sm-D autoantigen) - Homo sapiens (Human)	67	13273	12	18.5
88	RL35A	60S ribosomal protein L35a - Homo sapiens (Human)	63	12530	24	21.8

89	TCPD	T-complex protein 1 subunit delta (TCP-1-delta) (CCT-delta) (Stimulator of TAR RNA-binding) - Homo sapiens (Human)	62	57888	31	36.7
90	RS29	40S ribosomal protein S29 - Homo sapiens (Human)	57	6672	27	46.4
91	MYO1C	Myosin-Ic (Myosin I beta) (MMI-beta) (MMIb) - Homo sapiens (Human)	56	117965	24	13.7
92	TCPA	T-complex protein 1 subunit alpha (TCP-1-alpha) (CCT-alpha) - Homo sapiens (Human)	56	60306	20	25.9
93	ATPB	ATP synthase subunit beta, mitochondrial precursor (EC 3.6.3.14) - Homo sapiens (Human)	55	56525	18	29.9
94	TCPQ	T-complex protein 1 subunit theta (TCP-1-theta) (CCT-theta) (Renal carcinoma antigen NY-REN-15) - Homo sapiens (Human)	54	59583	21	24.5
95	HSP7C	Heat shock cognate 71 kDa protein (Heat shock 70 kDa protein 8) - Homo sapiens (Human)	49	70854	20	13.8
96	RL38	60S ribosomal protein L38 - Homo sapiens (Human)	34	8213	25	34.3
97	RL40	60S ribosomal protein L40 (CEP52) - Homo sapiens (Human)	34	6177	8	13.5
98	LYAR	Cell growth-regulating nucleolar protein - Homo sapiens (Human)	33	43588	4	7.9
99	IF6	Eukaryotic translation initiation factor 6 (eIF-6) (B4 integrin interactor) (CAB) (p27(BBP)) (B(2)GCN homolog) - Homo sapiens (Human)	30	26582	10	24.1

100	PSB1	Proteasome subunit beta type 1 precursor (EC 3.4.25.1) (Proteasome component C5) (Macropain subunit C5) (Multicatalytic endopeptidase complex subunit C5) (Proteasome gamma chain) - Homo sapiens (Human)	30	26472	3	7.5
101	TCPZ	T-complex protein 1 subunit zeta (TCP-1-zeta) (CCT-zeta) (CCT-zeta-1) (Tcp20) (HTR3) (Acute morphine dependence-related protein 2) - Homo sapiens (Human)	29	57988	13	19.6
102	IGHG1	Ig gamma-1 chain C region - Homo sapiens (Human)	29	36083	7	2.4
103	PRS6A	26S protease regulatory subunit 6A (Proteasome 26S subunit ATPase 3) (Tat-binding protein 1) (TBP-1) (Proteasome subunit P50) - Homo sapiens (Human)	28	49172	2	8.2
104	RL37A	60S ribosomal protein L37a - Homo sapiens (Human)	26	10268	17	54.3
105	SMD3	Small nuclear ribonucleoprotein Sm D3 (snRNP core protein D3) (Sm-D3) - Homo sapiens (Human)	24	13907	2	8.7
106	ALBU	Serum albumin precursor - Homo sapiens (Human)	24	69321	6	7.6
107	H13	Histone H1.3 (Histone H1c) - Homo sapiens (Human)	24	22336	7	14.5
108	PSA4	Proteasome subunit alpha type 4 (EC 3.4.25.1) (Proteasome component C9) (Macropain subunit C9) (Multicatalytic endopeptidase complex subunit C9) (Proteasome subunit L) - Homo sapiens (Human)	23	29465	1	3.4

109	EF1A1	Elongation factor 1-alpha 1 (EF-1-alpha-1) (Elongation factor 1 A-1) (eEF1A-1) (Elongation factor Tu) (EF-Tu) (Leukocyte receptor cluster member 7) - Homo sapiens (Human)	23	50109	8	21.4
110	RS28	40S ribosomal protein S28 - Homo sapiens (Human)	22	7836	7	29
111	RL39	60S ribosomal protein L39 - Homo sapiens (Human)	22	6403	7	23.5
112	PICK1	PRKCA-binding protein (Protein kinase C-alpha-binding protein) (Protein interacting with C kinase 1) - Homo sapiens (Human)	20	46571	9	4.6
113	SYLC	Leucyl-tRNA synthetase, cytoplasmic (EC 6.1.1.4) (Leucine--tRNA ligase) (LeuRS) - Homo sapiens (Human)	19	134379	5	4
114	PRS6B	26S protease regulatory subunit 6B (Proteasome 26S subunit ATPase 4) (MIP224) (MB67-interacting protein) (TAT-binding protein 7) (TBP-7) - Homo sapiens (Human)	18	47337	5	12.2
115	PRAM7	PRAME family member 7 - Homo sapiens (Human)	18	53617	2	1.3
116	TITIN	Titin (EC 2.7.11.1) (Connectin) (Rhabdomyosarcoma antigen MU-RMS-40.14) - Homo sapiens (Human)	17	3813810	20	0.2
117	PSB3	Proteasome subunit beta type 3 (EC 3.4.25.1) (Proteasome theta chain) (Proteasome chain 13) (Proteasome component C10-II) - Homo sapiens (Human)	17	22933	4	8.8

Table S2 | 284 genes translationally reduced in Silvestrol						
Refseq name	RF DMSO (rpkm)	RF Silvestrol (rpkm)	mRNA DMSO (rpkm)	mRNA Silvestrol (rpkm)	∆ TE	z-score
PAFAH1B1	108.22	11.80	19.51	28.85	-15.50	-3.64
ZNF664	16.37	1.92	14.28	18.90	-14.59	-3.55
ARF6	437.98	79.25	58.64	86.33	-13.14	-3.40
KIAA0754	9.54	1.20	12.01	17.67	-12.19	-3.29
HOMER1	5.13	0.55	1.67	2.02	-11.72	-3.23
CCND2	4.35	1.03	0.36	0.53	-11.53	-3.21
GSK3B	32.31	3.53	6.60	8.27	-11.39	-3.19
GPBP1L1	10.50	1.81	16.01	20.51	-9.76	-2.97
CDC42SE2	112.04	16.01	8.94	11.46	-9.42	-2.91
ARHGEF11	6.95	1.07	9.79	11.21	-9.13	-2.87
CNTROB	8.29	1.59	23.55	32.41	-8.95	-2.84
ASPHD1	13.17	1.69	9.06	9.71	-8.93	-2.84
TLE1	19.37	2.96	17.42	17.75	-8.78	-2.81
RAD17	3.89	0.77	3.75	5.48	-8.76	-2.81
SRGAP2B	65.16	9.39	18.97	18.37	-8.68	-2.80
CDC42BPA	22.27	4.30	14.21	21.23	-8.40	-2.75
UBL3	27.36	5.76	5.48	6.74	-8.35	-2.74
ARHGEF9	5.77	0.90	4.15	5.94	-8.34	-2.74
TAOK1	26.89	3.32	10.97	10.08	-8.31	-2.73
DDX6	118.73	25.11	18.38	26.63	-8.23	-2.72
ZNF45	2.24	0.58	4.40	6.53	-8.19	-2.71
ANKRD42	7.46	1.13	2.06	2.21	-8.10	-2.69
MORC2	17.93	3.84	8.49	11.88	-8.08	-2.69
XPO1	162.63	34.48	44.30	63.89	-8.06	-2.69
FLNC	68.16	9.09	45.65	36.19	-7.96	-2.67
HCFC1	35.39	6.49	24.21	29.75	-7.94	-2.67
CAB39	141.76	24.38	25.54	34.34	-7.91	-2.66
TBC1D5	18.97	3.23	5.47	6.56	-7.47	-2.58
SRGAP2	17.05	4.05	10.22	12.95	-7.44	-2.57
RNF4	90.87	11.42	33.15	28.37	-7.41	-2.57
BHLHB9	3.55	0.64	1.18	1.60	-7.37	-2.56
C10orf2	10.53	2.37	8.51	11.81	-7.22	-2.53
NCOA1	7.32	1.28	3.68	4.87	-7.17	-2.52
BCL2	4.08	0.99	0.96	1.55	-7.04	-2.49
ARHGEF12	32.72	5.98	30.52	40.75	-7.03	-2.49
BUD31	270.46	51.82	69.17	86.62	-7.02	-2.49
PPP1R9B	7.06	1.50	24.98	22.03	-6.95	-2.47
MKKS	7.79	2.02	29.99	40.25	-6.87	-2.45
NR2F1	3.47	0.95	1.78	2.66	-6.83	-2.45
SAMD12	4.71	1.65	0.59	0.64	-6.78	-2.43
SMURF2	80.95	19.66	59.16	76.32	-6.70	-2.42
VEPH1	1.78	0.45	0.76	0.94	-6.68	-2.41
H1F0	290.32	64.80	87.37	100.97	-6.64	-2.40

FYN	17.50	3.44	10.75	12.01	-6.58	-2.39
QKI	127.51	23.89	14.75	16.08	-6.58	-2.39
HPS4	6.74	1.49	6.23	9.13	-6.50	-2.37
PTENP1	7.08	1.30	1.97	2.18	-6.44	-2.36
CASKIN2	6.12	1.60	6.94	8.10	-6.42	-2.36
PPP2CB	61.56	8.25	36.74	30.14	-6.38	-2.35
APAF1	15.48	3.64	4.84	5.72	-6.36	-2.34
HNRNPD	602.22	141.88	92.44	117.42	-6.30	-2.33
HEXIM1	123.08	28.23	28.88	39.71	-6.28	-2.32
YAP1	97.40	21.89	30.98	34.04	-6.07	-2.27
SNX33	14.00	2.26	13.51	11.62	-6.06	-2.27
SYTL4	7.23	1.93	2.84	3.43	-6.05	-2.27
PLXNA2	1.91	0.29	4.73	5.46	-6.01	-2.26
PPP1R3D	8.29	1.81	3.30	3.65	-6.01	-2.26
SMEK2	28.71	7.87	8.92	12.69	-6.01	-2.26
TARDBP	56.75	23.16	10.06	12.09	-5.95	-2.25
ARAP2	1.23	0.38	0.73	0.98	-5.94	-2.24
CNOT6	22.67	5.69	9.94	14.15	-5.91	-2.24
TSC22D4	14.30	2.35	33.17	25.87	-5.91	-2.24
MED13L	8.62	2.21	8.78	13.14	-5.91	-2.24
GRLF1	20.90	4.76	20.71	25.66	-5.86	-2.22
AHDC1	4.95	1.51	7.95	10.02	-5.73	-2.19
PDZD2	0.47	0.17	0.86	1.15	-5.51	-2.13
FAM196B	3.27	1.29	2.52	2.73	-5.49	-2.13
GNAI1	36.65	12.24	5.05	7.57	-5.49	-2.13
CNOT1	36.57	8.23	26.43	29.18	-5.46	-2.12
JOSD1	22.29	4.36	30.84	29.69	-5.39	-2.10
TMEM65	41.54	12.49	4.82	6.72	-5.35	-2.09
C10orf18	16.49	5.46	9.62	13.67	-5.32	-2.08
ENOX2	6.10	1.47	3.04	3.44	-5.31	-2.08
SOX4	4.78	1.23	3.14	3.66	-5.30	-2.08
MAGI1	10.53	2.32	7.61	9.13	-5.28	-2.07
CENPN	56.19	14.75	13.49	16.18	-5.21	-2.05
MPP5	92.86	25.94	16.79	22.39	-5.16	-2.04
TCF12	31.91	10.18	9.19	12.41	-5.16	-2.04
MAP3K11	6.96	1.20	48.78	37.37	-5.16	-2.04
TPX2	357.83	112.81	93.42	119.76	-5.13	-2.03
FAM49B	142.36	38.82	31.20	44.90	-5.11	-2.02
ELK3	45.94	10.24	26.50	30.79	-5.11	-2.02
WNK1	22.90	6.86	14.98	19.83	-5.09	-2.02
OTUD4	4.82	1.20	2.51	3.53	-5.08	-2.02
FOSL2	119.13	33.92	47.35	60.90	-5.07	-2.01
MAN1A2	5.50	1.20	5.13	7.07	-5.06	-2.01
SIPA1L1	12.22	3.72	14.44	19.09	-5.04	-2.00
ARMCX3	31.45	9.62	8.44	11.23	-5.02	-2.00
ZHX1	10.21	3.10	5.74	8.49	-4.98	-1.99
KIAA2018	0.58	0.17	1.69	2.39	-4.97	-1.98

JAK2	2.23	0.76	1.00	1.50	-4.96	-1.98
CDK6	89.23	25.08	8.75	11.17	-4.95	-1.98
GPRASP2	7.96	1.66	3.86	3.22	-4.93	-1.97
RTN4IP1	7.37	2.02	2.06	2.19	-4.91	-1.96
RBBP7	636.60	156.92	144.47	172.97	-4.90	-1.96
CSNK1G1	9.77	3.58	3.02	4.09	-4.89	-1.96
STXBP5	4.84	1.20	2.50	2.62	-4.87	-1.95
WNK3	2.54	0.71	0.89	0.93	-4.85	-1.95
WHSC1L1	6.30	1.94	4.15	5.17	-4.85	-1.95
PPP1CC	473.90	130.02	98.51	135.02	-4.83	-1.94
ZHX3	15.52	4.96	8.50	10.89	-4.82	-1.94
KIF4B	8.10	2.64	5.03	4.99	-4.81	-1.94
SCYL2	10.37	3.13	4.71	6.14	-4.81	-1.93
TEAD1	95.34	28.64	25.39	33.27	-4.81	-1.93
FAM43A	3.10	0.58	7.26	6.26	-4.81	-1.93
CSNK1G2	11.41	2.50	63.32	46.95	-4.79	-1.93
ANKRD13C	24.26	6.42	6.62	9.18	-4.78	-1.93
LMO4	86.76	22.62	8.30	11.14	-4.78	-1.93
MTMR15	5.01	1.50	3.35	3.98	-4.78	-1.92
TAOK2	5.03	1.18	12.41	14.10	-4.73	-1.91
CDK8	9.64	2.83	3.89	4.62	-4.73	-1.91
PEG10	45.26	12.48	8.29	9.50	-4.72	-1.91
C18orf55	28.93	6.58	10.43	11.85	-4.70	-1.90
SPIN3	6.05	2.47	1.84	2.58	-4.70	-1.90
TOR1AIP2	75.20	18.07	13.36	12.21	-4.69	-1.90
MST4	25.72	7.48	4.41	5.23	-4.69	-1.90
FAM55C	5.91	1.34	5.09	6.47	-4.67	-1.89
ROCK1	23.69	7.71	7.51	9.37	-4.67	-1.89
CSNK1A1	104.98	33.60	38.72	49.61	-4.61	-1.87
OXNAD1	15.58	4.86	3.23	3.73	-4.60	-1.87
FOXJ2	10.54	2.78	5.81	7.39	-4.60	-1.87
CEP164	8.54	3.22	10.40	14.76	-4.53	-1.85
FRYL	13.84	5.38	6.38	9.54	-4.52	-1.84
SET	59.22	11.28	76.53	75.00	-4.52	-1.84
PXMP3	20.47	5.30	5.84	6.77	-4.51	-1.84
ALKBH3	34.23	10.55	16.55	19.68	-4.50	-1.84
C18orf25	24.52	9.15	7.30	10.90	-4.50	-1.84
DCLRE1A	6.51	2.64	3.26	4.82	-4.49	-1.84
GABPB2	6.39	2.75	3.05	4.00	-4.48	-1.83
MUDENG	23.11	7.06	7.71	11.03	-4.47	-1.83
NUDT4	14.79	5.41	1.12	1.05	-4.47	-1.83
STK11	12.51	2.76	37.28	32.28	-4.46	-1.83
C3orf63	23.79	7.87	8.32	11.41	-4.46	-1.83
PTPN21	6.58	2.12	7.16	9.68	-4.45	-1.82
RAI1	7.19	2.88	9.81	12.40	-4.44	-1.82
CLOCK	15.91	3.67	7.16	6.72	-4.43	-1.82
ZDHHC5	11.79	2.50	32.06	37.00	-4.42	-1.81

TAF7	195.99	61.64	77.99	108.96	-4.42	-1.81
TTLL5	8.60	3.07	8.62	11.54	-4.42	-1.81
ANKRA2	6.82	2.41	4.17	4.79	-4.41	-1.81
ZFP91	44.06	14.89	26.44	37.82	-4.41	-1.81
ZNF740	23.35	7.99	8.44	10.02	-4.40	-1.81
NONO	145.82	42.87	35.86	36.04	-4.40	-1.80
MAP3K7IP3	7.11	2.15	6.23	8.56	-4.38	-1.80
CSNK2A1	57.42	30.47	25.21	23.66	-4.36	-1.79
LOC284441	76.64	18.71	3.66	3.02	-4.36	-1.79
CDC2L5	13.70	5.07	8.43	12.31	-4.36	-1.79
NXT1	73.25	13.60	53.64	45.23	-4.35	-1.79
IPO8	39.62	12.52	11.59	14.23	-4.34	-1.78
TCEAL8	93.69	32.20	12.76	17.05	-4.33	-1.78
UBE2H	134.32	28.55	23.33	23.37	-4.32	-1.78
FASTKD1	2.46	0.80	1.68	1.87	-4.31	-1.78
PIK3R3	4.75	1.54	1.20	1.75	-4.30	-1.77
NFE2L1	23.90	4.69	65.43	62.65	-4.30	-1.77
BCL9	4.22	1.41	3.96	4.67	-4.30	-1.77
KLHL2	5.77	2.49	6.98	10.32	-4.30	-1.77
ZNF609	6.96	2.70	6.12	7.35	-4.29	-1.77
PUM2	45.26	16.75	19.83	28.02	-4.29	-1.77
FAM126A	34.62	12.15	8.68	12.88	-4.28	-1.77
RAB10	280.66	79.92	32.52	37.36	-4.27	-1.76
DUSP16	5.36	2.03	4.12	5.80	-4.27	-1.76
USP9X	59.40	16.01	20.04	21.86	-4.27	-1.76
BBX	55.07	18.10	12.88	15.26	-4.25	-1.76
FKTN	9.55	2.15	4.01	4.45	-4.24	-1.75
CHST10	5.74	1.32	8.55	8.95	-4.24	-1.75
PTER	38.21	13.83	4.89	6.72	-4.23	-1.75
WDTC1	12.48	2.14	18.26	12.75	-4.23	-1.75
SMARCD3	13.51	2.78	12.01	8.50	-4.20	-1.74
PITPNA	80.43	20.72	25.34	26.42	-4.20	-1.74
TMEM184C	10.56	1.95	9.35	10.81	-4.20	-1.74
ABCE1	40.40	11.51	11.04	11.44	-4.19	-1.74
TRIM44	70.92	23.82	20.78	29.98	-4.19	-1.74
KIAA1211	1.03	0.46	0.42	0.59	-4.19	-1.73
SH3BP5L	12.60	3.62	21.64	19.55	-4.18	-1.73
ITPKB	1.09	0.41	1.28	1.09	-4.18	-1.73
ATN1	14.79	3.57	32.48	30.03	-4.17	-1.73
C10orf46	33.78	12.17	11.18	15.33	-4.14	-1.72
XPO6	21.38	5.76	39.64	38.15	-4.14	-1.72
MAPKAP1	47.36	10.24	46.26	37.89	-4.14	-1.72
ZNF521	1.01	0.36	0.63	0.84	-4.13	-1.71
GNG12	621.40	143.59	47.62	45.73	-4.13	-1.71
H2AFY	366.70	86.90	156.92	147.19	-4.13	-1.71
CCDC80	8.09	1.70	16.04	16.60	-4.12	-1.71
FAM135A	1.93	1.02	1.28	1.76	-4.12	-1.71

HS2ST1	17.97	4.51	6.99	9.29	-4.12	-1.71
TOB2	22.18	9.78	7.21	9.94	-4.11	-1.71
XRCC3	24.06	7.27	26.77	24.93	-4.11	-1.71
BTBD7	7.56	2.59	5.05	6.70	-4.10	-1.70
MAML2	18.01	6.35	8.44	11.55	-4.10	-1.70
RCBTB1	15.71	5.85	5.11	6.94	-4.09	-1.70
TCHP	32.27	13.77	10.67	15.00	-4.08	-1.70
BTBD3	22.69	9.22	6.86	10.11	-4.08	-1.70
HNRNPH3	305.88	112.80	38.07	47.44	-4.08	-1.70
KYNU	6.40	2.38	1.15	1.40	-4.07	-1.69
AFF3	4.58	2.38	2.03	2.71	-4.07	-1.69
ARSJ	4.06	1.07	7.41	7.22	-4.07	-1.69
ZNF672	5.52	1.68	20.37	19.82	-4.05	-1.68
KLHL8	16.55	6.63	3.15	3.97	-4.04	-1.68
SPATS2	16.65	6.93	7.13	10.58	-4.03	-1.68
ANKIB1	15.66	4.91	11.64	15.30	-4.03	-1.68
SAMD4B	22.91	5.82	21.45	19.79	-4.02	-1.67
YME1L1	115.37	47.11	28.96	42.63	-4.01	-1.67
ROCK2	44.87	18.35	10.29	14.62	-4.01	-1.67
FBXL19	2.32	0.64	9.21	8.03	-4.00	-1.67
ZFYVE1	13.38	4.48	8.64	10.64	-3.98	-1.66
HIPK1	22.65	8.84	9.62	13.82	-3.98	-1.66
LUZP1	25.09	9.00	9.85	11.38	-3.98	-1.66
WDR24	5.50	1.42	7.36	6.58	-3.97	-1.66
SAP30	42.94	19.53	25.66	30.45	-3.96	-1.65
SMAD5	74.74	28.52	10.14	13.53	-3.95	-1.65
RAB9B	8.73	1.44	1.36	1.03	-3.95	-1.65
KIAA2026	0.54	0.22	0.95	1.38	-3.95	-1.65
SETD3	36.86	12.54	19.68	24.17	-3.94	-1.65
L3MBTL3	4.18	2.38	1.15	1.67	-3.94	-1.65
PTPN11	7.72	2.24	11.35	9.82	-3.94	-1.64
PNMA2	43.83	9.91	11.43	9.43	-3.94	-1.64
JRK	7.57	2.24	5.94	6.49	-3.93	-1.64
WDR26	79.72	30.23	17.21	24.33	-3.93	-1.64
GRK5	4.23	1.20	7.05	7.43	-3.92	-1.64
C1orf109	32.96	16.54	9.69	13.85	-3.92	-1.64
JPH1	1.75	0.70	0.59	0.83	-3.91	-1.63
LOC552889	94.29	24.34	20.50	19.69	-3.90	-1.63
ERI1	77.38	30.18	7.72	11.30	-3.90	-1.63
UBE2D2	284.11	85.40	46.80	49.96	-3.89	-1.63
FAM168B	154.49	55.32	31.81	44.67	-3.89	-1.62
ARMCX5	17.52	6.77	5.10	6.98	-3.87	-1.62
EXOC5	13.60	6.76	4.57	6.65	-3.86	-1.62
NUCKS1	522.60	171.82	70.17	87.45	-3.85	-1.61
LRSAM1	17.54	5.14	11.44	11.98	-3.85	-1.61
CAB39L	8.58	3.00	2.37	3.10	-3.84	-1.61
DBF4B	6.51	2.59	6.64	8.33	-3.84	-1.61

CNKSR3	13.79	5.54	4.85	6.36	-3.84	-1.61
TAF4B	2.44	0.87	1.85	2.60	-3.83	-1.60
ADARB1	3.18	1.65	3.08	3.81	-3.83	-1.60
NEK11	3.31	1.29	1.54	1.95	-3.82	-1.60
BAZ1A	21.87	9.90	6.27	8.53	-3.82	-1.60
ARL6	14.65	7.43	1.87	2.71	-3.81	-1.60
CSNK1E	67.61	19.77	39.35	42.01	-3.81	-1.60
SHB	26.98	7.45	17.03	16.77	-3.81	-1.59
SPIN1	74.58	23.20	20.11	23.32	-3.79	-1.59
PFTK1	7.56	3.65	1.83	2.62	-3.79	-1.59
CDC2L6	9.85	3.06	3.22	3.74	-3.79	-1.59
NCK2	15.77	4.85	17.39	22.12	-3.79	-1.59
KIAA0430	6.96	2.13	5.90	5.87	-3.79	-1.59
NSUN7	2.89	0.94	1.09	1.61	-3.79	-1.59
ANLN	276.65	112.54	79.13	89.50	-3.78	-1.59
FAM178A	19.05	8.14	7.43	8.73	-3.77	-1.58
SYNCRIP	82.75	32.85	14.93	18.33	-3.76	-1.58
SAMD9L	6.81	1.77	3.39	4.12	-3.76	-1.58
GRB10	35.31	10.49	20.09	20.85	-3.76	-1.58
KIAA0355	6.10	2.58	4.80	6.35	-3.76	-1.57
PITPNC1	9.08	2.87	4.94	5.27	-3.75	-1.57
UBP1	16.85	7.15	19.07	24.99	-3.75	-1.57
NR2F2	101.53	45.16	27.70	39.53	-3.74	-1.57
USP7	38.42	14.99	12.49	16.72	-3.74	-1.57
NLRP10	2.43	1.17	0.44	0.47	-3.74	-1.57
PPP2R5A	20.59	6.12	10.32	10.81	-3.73	-1.57
C7orf49	49.09	18.47	42.03	47.31	-3.73	-1.57
RAD21	241.17	120.68	55.46	82.81	-3.73	-1.56
DGKH	3.01	1.57	0.96	1.36	-3.71	-1.56
KPNB1	566.33	189.14	146.78	174.65	-3.70	-1.56
IRS1	26.45	10.28	15.91	20.83	-3.70	-1.55
PUS1	24.02	9.46	17.13	20.54	-3.69	-1.55
DNAJC13	13.01	5.17	7.35	9.95	-3.68	-1.54
RECQL	73.13	27.61	9.44	10.77	-3.66	-1.54
HNRNPH2	145.79	54.78	22.85	27.50	-3.66	-1.54
C1orf58	108.82	47.30	11.89	17.52	-3.65	-1.54
DHX32	34.51	11.37	15.99	19.50	-3.65	-1.53
CUL1	60.55	19.45	26.00	28.64	-3.64	-1.53
DLG1	65.12	25.44	21.44	24.92	-3.63	-1.53
LRRC1	16.82	5.73	4.62	5.61	-3.63	-1.52
YWHAH	389.85	124.73	83.99	103.22	-3.63	-1.52
PIK3CA	12.08	3.93	5.10	5.31	-3.61	-1.52
SAPS3	118.43	51.30	22.58	33.64	-3.61	-1.52
CCND1	1090.52	395.22	170.53	233.37	-3.61	-1.52
LEF1	3.89	1.77	1.36	1.90	-3.60	-1.51
EFCAB4B	4.77	1.61	3.14	3.10	-3.59	-1.51
HNRPLL	58.64	21.24	15.14	16.84	-3.59	-1.51

TFCP2	18.83	8.06	6.25	8.31	-3.59	-1.51
NOD1	1.28	0.43	2.53	3.13	-3.57	-1.50
POLR2A	67.46	27.39	28.64	34.14	-3.57	-1.50
GINS1	92.87	33.02	12.12	14.09	-3.57	-1.50
MKL2	7.47	3.26	2.27	2.99	-3.57	-1.50
MLLT6	3.53	1.01	17.16	14.26	-3.57	-1.50

Table S3 | 146 genes translationally increased in Silvestrol						
Refseq name	RF DMSO (rpkm)	RF Silvestrol (rpkm)	mRNA DMSO (rpkm)	mRNA Silvestrol (rpkm)	Δ TE	z-score
ANXA2	580.18	945.60	196.63	140.00	19.00	4.64
C17orf62	24.34	24.23	39.46	28.72	4.56	2.56
ABCC3	2.23	3.35	12.87	9.90	4.09	2.41
S100A4	3695.17	5143.25	1702.79	1227.52	3.71	2.26
TACSTD2	28.16	37.64	52.82	35.58	3.69	2.26
TMEM179B	38.15	65.22	60.23	40.21	3.69	2.26
IGFBP7	424.59	658.30	964.28	670.28	3.59	2.22
PPL	1.68	3.02	5.28	4.06	3.54	2.20
PPAP2C	26.13	34.81	48.27	33.97	3.52	2.19
ENDOD1	10.62	13.49	17.97	13.92	3.52	2.19
ICMT	37.63	50.62	42.99	29.22	3.43	2.15
PIGU	11.44	15.76	20.97	15.79	3.33	2.11
LEPROTL1	25.20	31.82	10.27	7.02	3.21	2.05
COL6A1	9.96	15.05	34.46	24.01	3.18	2.04
LSR	6.77	9.31	18.43	13.28	3.14	2.02
MAN2B2	6.50	9.33	18.23	12.41	3.10	2.00
TNFSF10	78.90	87.16	34.59	36.59	3.05	1.98
PIGT	33.28	53.30	111.83	74.68	3.04	1.97
TM9SF4	30.20	38.49	37.40	25.89	2.98	1.95
POLR2L	2729.24	4800.23	588.55	412.64	2.97	1.94
ABCC1	10.03	15.86	20.42	15.24	2.90	1.91
TMEM222	36.60	51.82	34.23	24.74	2.87	1.89
S100A6	31483.13	54735.59	10200.04	7135.10	2.86	1.89
SH3BGRL3	1713.32	2605.80	457.86	305.30	2.86	1.89
ACTB	2360.49	2010.27	1728.67	1407.16	2.86	1.89
C22orf39	24.07	48.01	11.52	8.29	2.85	1.88
SLC39A13	35.28	58.46	92.64	65.94	2.83	1.87
RETSAT	15.92	23.07	26.54	22.52	2.83	1.87
PEX11B	20.82	30.20	11.76	8.22	2.81	1.86
POFUT2	11.74	16.00	22.68	15.46	2.81	1.86
AIG1	28.34	42.25	15.14	12.81	2.77	1.84
GPRC5A	152.19	199.35	93.83	63.71	2.76	1.83
POR	12.04	17.85	32.79	24.81	2.75	1.83
SLC35E1	16.42	22.86	25.09	18.00	2.74	1.82
SGSH	4.83	8.00	12.28	9.02	2.73	1.82
CCDC107	22.41	39.14	61.54	45.63	2.71	1.80
SUMF2	78.50	112.69	125.75	95.46	2.70	1.80
TNFSF9	9.04	15.98	12.56	11.31	2.70	1.80
CLPTM1	31.14	43.69	92.24	66.01	2.68	1.79
RCE1	15.55	23.02	23.97	20.32	2.68	1.79
DOLPP1	16.15	30.63	16.89	13.90	2.67	1.78
HEG1	12.78	16.92	38.01	32.07	2.66	1.78
ZMYM6	46.68	61.73	26.20	22.49	2.66	1.78

ERAP1	16.99	18.39	9.87	6.83	2.64	1.77
SSNA1	67.31	113.32	159.63	112.77	2.63	1.76
KDELR1	299.29	333.95	203.54	157.24	2.61	1.75
P4HB	224.00	329.00	482.32	338.26	2.61	1.75
RPN1	78.61	112.92	143.75	108.28	2.61	1.75
RPS4X	71.28	115.15	221.44	162.67	2.60	1.75
DNAJC30	26.13	46.21	8.79	6.71	2.59	1.74
KDELR2	222.33	315.33	112.43	106.04	2.59	1.74
C19orf56	656.86	952.13	248.70	172.88	2.58	1.74
RABAC1	192.49	241.07	199.58	136.61	2.58	1.73
TMBIM1	80.83	84.70	127.02	85.11	2.57	1.73
MMAB	49.02	75.29	14.42	11.58	2.56	1.72
TMEM92	22.12	24.05	8.46	6.09	2.53	1.71
SIAE	7.51	9.91	7.82	7.08	2.53	1.71
TNFRSF12A	318.88	471.65	374.89	310.04	2.52	1.70
SIRT3	10.85	18.97	7.67	5.41	2.52	1.70
TMED10	120.92	170.86	36.06	29.65	2.52	1.70
LIPG	16.11	15.93	12.32	9.94	2.51	1.70
APOL1	16.04	12.89	10.43	7.29	2.51	1.70
RHBDD2	11.69	15.06	27.86	25.41	2.50	1.69
C19orf10	274.73	347.78	338.15	261.22	2.49	1.68
RPL4	100.35	90.55	94.16	65.84	2.49	1.68
MED9	27.09	48.43	11.33	9.80	2.49	1.68
NDUFAF3	224.44	386.56	77.41	55.37	2.49	1.68
C16orf58	31.30	43.70	41.47	28.72	2.48	1.68
SPPL2B	4.42	6.36	10.24	9.60	2.47	1.67
NUDT8	18.44	33.97	16.34	14.38	2.47	1.67
CTSH	14.90	17.59	28.83	21.92	2.46	1.67
DPP7	22.93	33.31	125.37	85.31	2.46	1.66
LTBP1	2.44	2.71	7.26	5.69	2.46	1.66
URM1	166.17	231.29	93.59	65.99	2.45	1.66
MSRB2	89.29	114.25	34.91	23.53	2.45	1.66
RPLP2	764.19	854.45	589.50	597.22	2.45	1.66
LEMD2	23.04	37.07	53.82	42.93	2.45	1.66
TBL2	27.67	36.64	35.02	25.05	2.45	1.66
C20orf108	13.58	22.25	10.17	9.27	2.45	1.66
MCAM	11.91	17.65	25.37	17.60	2.45	1.66
RNF121	16.69	19.27	18.91	13.74	2.45	1.66
MYADM	127.98	194.51	84.97	80.03	2.45	1.66
ZNF511	54.37	80.33	105.65	72.18	2.44	1.65
MOCS3	12.85	22.53	6.06	4.39	2.43	1.65
CD40	35.66	48.26	45.86	36.26	2.43	1.65
VEGFB	9.06	17.66	105.33	74.54	2.43	1.65
LEPRE1	21.66	29.60	50.45	37.09	2.43	1.65
CEBPD	27.02	78.33	33.35	43.99	2.43	1.65
GDE1	9.88	14.69	28.53	27.03	2.42	1.64
WFS1	18.01	28.23	23.83	19.31	2.42	1.64

ALG8	34.71	50.29	46.74	39.96	2.41	1.64
ERP29	62.61	84.39	103.07	82.65	2.41	1.64
C2orf79	133.85	187.04	113.36	80.48	2.40	1.63
LMF2	16.79	26.81	49.92	40.56	2.39	1.63
LSS	135.63	158.92	99.81	67.60	2.39	1.63
B4GALT7	6.64	8.96	27.04	20.24	2.37	1.61
SLC27A1	6.23	8.52	14.08	11.00	2.36	1.61
DHCR24	64.22	66.25	102.11	74.87	2.36	1.61
PTK6	10.76	16.28	8.36	6.45	2.36	1.60
TXNDC5	179.03	269.27	226.74	172.15	2.35	1.60
POLD4	104.89	135.60	100.16	69.39	2.35	1.60
PPIB	482.97	645.32	493.22	409.82	2.34	1.59
KIAA0090	18.91	25.72	19.91	13.37	2.33	1.59
SLC17A5	16.69	19.23	18.45	15.57	2.32	1.58
TPP1	22.98	28.87	28.57	20.83	2.32	1.58
C4orf48	64.81	98.03	139.75	96.33	2.32	1.58
EFEMP1	43.30	54.05	36.42	30.77	2.32	1.58
COMMD9	84.75	120.27	19.87	14.13	2.32	1.58
LAYN	20.72	23.80	48.12	34.30	2.32	1.58
MARCH6	30.42	37.44	27.46	20.68	2.32	1.58
ADPRHL2	64.31	98.89	61.59	42.42	2.30	1.57
SIGMAR1	100.64	115.36	117.51	79.97	2.30	1.57
SSR2	267.94	276.87	356.75	241.53	2.30	1.56
RPL14	389.17	552.34	199.95	143.62	2.29	1.56
PRCP	39.77	48.02	64.18	43.35	2.27	1.55
TNFRSF10A	21.37	29.01	16.37	12.68	2.27	1.55
DHRS7B	25.07	36.09	34.24	26.72	2.27	1.55
OVCA2	42.89	63.96	75.71	55.85	2.26	1.54
C9orf5	14.91	22.21	20.64	20.08	2.26	1.54
COX8A	2107.61	2939.84	950.56	764.02	2.25	1.53
LAMP1	70.92	111.73	101.26	88.41	2.25	1.53
PPT1	64.50	94.80	37.72	33.31	2.25	1.53
UQCRQ	1203.23	1392.77	141.86	94.85	2.25	1.53
KCNN4	24.98	29.19	43.07	33.20	2.24	1.53
ERGIC3	123.93	147.48	282.35	192.77	2.24	1.53
ITGB4	10.12	11.48	50.27	33.55	2.24	1.53
HDHD1A	38.54	50.69	19.12	13.52	2.24	1.53
POFUT1	39.19	55.61	43.83	32.58	2.24	1.53
PIGO	7.23	10.17	14.00	10.54	2.23	1.52
MRPL53	220.60	317.76	99.49	81.02	2.23	1.52
SURF1	26.91	41.44	61.84	43.38	2.23	1.52
CRELD1	7.29	7.76	12.73	10.63	2.23	1.52
SIVA1	139.87	204.86	273.31	187.29	2.23	1.52
CHMP6	48.80	63.63	39.07	26.51	2.22	1.52
ZNF593	77.15	123.25	78.92	52.99	2.22	1.52
GUSB	12.19	16.65	36.56	28.22	2.22	1.52
ARL6IP5	171.95	177.43	51.35	36.91	2.22	1.52

PRDX4	135.30	182.90	260.14	199.52	2.22	1.51
RNASEK	570.51	751.96	145.07	112.03	2.21	1.51
FDX1L	42.83	73.17	56.60	41.18	2.21	1.51
MPDU1	89.33	123.71	76.72	62.49	2.21	1.51
RPS19BP1	87.76	128.05	119.24	81.55	2.21	1.51
PCSK9	18.83	19.78	39.86	38.49	2.20	1.50
ERAL1	101.68	154.06	81.72	57.45	2.20	1.50
COX7A2	495.75	710.79	492.55	354.05	2.20	1.50
PDDC1	64.70	100.34	28.41	22.07	2.20	1.50

Table S4 | 5'UTR characteristics of Silvestrol-sensitive and -insensitive genes

Gene name	ΔG value CONTRAfold	ΔG value McCaskill	5'UTR length	%GC content	ΔTE	Gene classification
ABCE1	-92.02	-50.38	565	55.22	-4.19	Sensitive
ADRBK1	-115.24	-89.19	340	84.71	-2.53	Sensitive
AGPAT6	-158.9	-51.63	977	51.28	-2.41	Sensitive
AHDC1*	-178.62	-69.57	673	66.12	-5.73	Sensitive
	-202.1	-124.3	1019	64.87		
ALKBH3	-130.85	-44.21	461	58.13	-4.50	Sensitive
ANKIB1	-100.41	-66.71	426	62.21	-4.03	Sensitive
ANKRA2	-135.92	-56.27	709	53.17	-4.41	Sensitive
ANKRD13C	-35.11	-35.61	364	67.03	-4.78	Sensitive
ANKRD42	-65.6	-44.43	212	64.62	-8.10	Sensitive
AP5M1 (MUDENG)	-77.61	-39.15	456	51.97	-4.47	Sensitive
APAF1	-210.47	-82.05	786	66.03	-6.36	Sensitive
ARAP2	-134.33	-49.57	540	61.85	-5.94	Sensitive
ARF3*	-76.35	-10.28	385	64.94	-2.66	Sensitive
	-70.12	-29.55	289	62.28		
ARF6	-264.47	-78.72	597	72.86	-13.14	Sensitive
ARHGEF11	-212.84	-103.55	1090	38.90	-9.13	Sensitive
ARHGEF12	-38.29	-7.42	216	51.85	-7.03	Sensitive
ARHGEF7	-120.98	-41.09	300	80.33	-2.80	Sensitive
ARHGEF9	-150	-74.61	851	54.05	-8.34	Sensitive
ARMCX2*	-28.74	-12.06	504	53.17	-3.02	Sensitive
	-101.39	-26.46	602	54.98		
	-48.19	-11.36	405	50.37		
ARMCX3*	-74.61	-57.26	916	56.11	-5.02	Sensitive
	-139	-10.84	596	56.21		
	-100.22	-35.23	506	56.13		
ARSJ	-237.51	-76.48	917	58.34	-4.07	Sensitive
ASPHD1*	-22.1	-2.1	302	61.26	-8.93	Sensitive
	-6.6	-3.18	164	58.54		
	-6.6	-3.18	164	58.54		
ASTN2	-43.28	-23.29	257	59.53	-3.20	Sensitive
ATN1*	-68.76	-21.66	287	52.96	-4.17	Sensitive
	-42.85	-19.43	284	56.34		
	-46.99	-23.64	215	49.30		
ATXN7L3	-19.11	-11.54	141	53.19	-3.21	Sensitive
BBX	-41.3	-14.44	337	51.63	-4.25	Sensitive
BCL9	-111.33	-35.63	790	50.51	-4.30	Sensitive
BHLHB9*	-67.86	-14.3	578	52.94	-7.37	Sensitive
	-109.71	-48.51	689	50.36		
	-91.92	-45.42	635	48.35		
	-125.22	-44.8	543	54.88		
	-106.48	-62.06	638	51.72		
BMPR2	-127.95	-75.94	589	59.59	-2.79	Sensitive

BTBD7	-47.16	-6.28	358	63.69	-4.10	Sensitive
BUD31*	-132.12	-47.52	483	53.00	-7.02	Sensitive
	-69.09	-19.02	579	57.34		
C10orf2	-43.96	-17.54	235	51.49	-7.22	Sensitive
C16orf48	-102.54	-53.5	372	76.34	-2.87	Sensitive
C6orf106	-75.2	-32.88	294	70.41	-3.04	Sensitive
CAB39*	-135.64	-70.41	479	74.95	-7.91	Sensitive
	-177.73	-85.36	604	59.11		
	-76.55	-29.02	348	61.21		
CAB39L*	-61.84	-16.92	548	41.61	-3.84	Sensitive
	-57.03	-31.39	395	42.78		
	-67.33	-22.9	436	45.41		
	-45.62	-17.14	501	40.52		
CACUL1	-204.28	-68.42	534	70.79	-4.14	Sensitive
CAMK1D	-140.17	-51.95	387	74.68	-3.02	Sensitive
CASKIN2	-98.08	-47.85	637	64.68	-6.42	Sensitive
CCDC80*	-61.92	-37.43	1004	46.02	-4.12	Sensitive
	-29.59	-6.92	438	43.38		
CCND1	-46.89	-14.01	277	64.62	-3.61	Sensitive
CCND2	-98.85	-44.15	319	63.01	-11.53	Sensitive
CCNI	-138.63	-44.5	627	64.75	-2.85	Sensitive
CDC42BPA	-288.01	-182.49	1342	53.06	-8.40	Sensitive
CDC42SE2*	-86.84	-32	596	45.30	-9.42	Sensitive
	-83.95	-39.1	493	51.52		
	-65.91	-17.29	571	43.96		
CDK6*	-108.33	-48.12	462	69.05	-4.95	Sensitive
	-147.97	-45.38	534	70.04		
CDK8	-150.46	-52.59	553	71.61	-4.73	Sensitive
CENPN	-170.77	-87.39	840	66.90	-5.21	Sensitive
CHMP4B	-58.3	-11.34	215	78.60	-3.33	Sensitive
CHST10*	-91.24	-38.6	436	69.27	-4.24	Sensitive
	-81.72	-56.4	317	57.41		
CLOCK*	-23.91	-10.09	301	35.55	-4.43	Sensitive
	-95.59	-30.29	976	42.11		
	-134.83	-85.77	696	54.17		
CMTM4	-105.86	-35.88	232	84.48	-3.08	Sensitive
CNN3*	-31.87	-15.5	133	64.66	-3.33	Sensitive
	-209.33	-107.71	434	73.27		
CNOT1	-51.89	-31.92	383	54.83	-5.46	Sensitive
CNOT6*	-56.59	-18.06	399	72.43	-5.91	Sensitive
	-121.41	-47.65	474	61.39		
CNTROB	-195.69	-87.19	975	58.15	-8.95	Sensitive
CSNK1E*	-13.05	-8.65	161	54.66	-3.81	Sensitive
	-80.8	-34.79	342	82.16		
	-40.35	-2.36	311	81.67		
	-104.11	-35.99	516	52.71		
	-36.95	-31.06	143	74.83		

CSNK1G1	-70.9	-29.77	474	52.11	-4.89	Sensitive
CSNK1G2	-137.85	-45.48	572	72.73	-4.79	Sensitive
CTNND1	-85.78	-40.45	586	49.32	-2.79	Sensitive
CTXN1	-118.8	-70.86	270	80.00	-3.17	Sensitive
CUL1*	-64.67	-39.27	400	58.75	-3.64	Sensitive
	-76.32	-25.66	576	46.18		
CUL3	-75.46	-36.78	389	76.61	-3.07	Sensitive
DARS2	-145.97	-66.24	777	52.51	-3.48	Sensitive
DDIT4L	-87.62	-58.13	265	66.04	-4.16	Sensitive
DDX6*	-42.16	-25.02	356	41.85	-8.23	Sensitive
	-36.49	-21.82	411	43.07		
	-40.72	-11.22	342	40.35		
DENND4B	-215.2	-89.16	469	78.04	-3.06	Sensitive
DGCR8	-93.5	-37.9	479	58.87	-3.07	Sensitive
DHX32	-54.04	-36.03	541	40.48	-3.65	Sensitive
DPYSL3	-99.6	-50.58	422	63.98	-2.38	Sensitive
DVL3	-103.43	-53.21	298	77.85	-3.05	Sensitive
DYNLL2	-120.61	-41.71	328	72.56	-3.49	Sensitive
EFCAB4B	-65.46	-7.77	524	60.50	-3.59	Sensitive
EFNB2	-61.1	-33.77	200	68.50	-2.10	Sensitive
EIF4EBP2	-15.74	-7.96	73	75.34	-2.26	Sensitive
ELK3	-95.2	-40.26	376	58.78	-5.11	Sensitive
ENOX1*	-86.3	-60.53	628	57.96	-3.13	Sensitive
	-44.88	-5.56	306	44.77		
ENOX2*	-97.4	-23.15	468	54.91	-5.31	Sensitive
	-1.9	-1.88	72	45.83		
EPB49*	-92.66	-52.3	322	72.05	-2.48	Sensitive
	-118.22	-50.5	512	66.80		
EXOC3*	6.24	-3.27	107	55.14	-3.47	Sensitive
	-8.5	-8.07	239	71.97		
EXT1	-137.4	-56.61	857	59.28	-2.54	Sensitive
FAM120AOS	-189.94	-81.81	933	49.52	-3.57	Sensitive
FAM126A	-75.33	-38.15	283	58.66	-4.28	Sensitive
FAM168B*	-80.15	-26.03	308	71.10	-3.89	Sensitive
	-32.35	-13.18	158	66.46		
FAM176A*	-58.45	-26.91	488	50.00	-3.08	Sensitive
	-89.47	-53.94	475	64.63		
	-71.7	-17.08	283	55.48		
	-105.71	-33.25	588	61.39		
FAM208A	0	-4.03	61	68.85	-4.46	Sensitive
FAM3A*	-155.78	-47.72	417	70.02	-1.66	Sensitive
	-66.02	-33.69	238	66.39		
	-129.98	-83.91	517	69.83		
FAM43A	-294.38	-141.83	984	65.55	-4.81	Sensitive
	-47.12	-34.43	427	52.93		
	-115.64	-56.85	451	62.31		
	-89.47	-36.23	324	66.05		

FAM49B*	-45.52	-24.9	305	50.82	-5.11	Sensitive
	-41.87	-15.97	371	51.48		
	-88.93	-43.5	599	53.92		
	-56.93	-32.36	234	58.97		
FAM53B*	-58.06	-22.91	262	62.60	-3.81	Sensitive
	-202.95	-106.08	767	54.50		
FAN1	-40.97	-32.5	341	51.61	-4.78	Sensitive
FASTKD1	-23.69	-8.16	397	42.82	-4.31	Sensitive
FBF1	-74.93	-27.84	324	67.28	-4.91	Sensitive
FBLIM1*	-145.84	-86.84	493	60.65	-3.56	Sensitive
	-210.96	-103.93	690	69.28		
FBXL14	-73.2	-36.76	149	73.15	-3.54	Sensitive
FBXL19	-60.11	-32.12	208	63.94	-4.00	Sensitive
FKTN	-4.3	0.53	174	41.38	-4.24	Sensitive
FLNC	-40.32	-22.81	311	73.63	-7.96	Sensitive
FLYWCH1	-153.44	-65.12	393	71.50	-2.08	Sensitive
FNBP1	-18.94	-61.99	269	76.58	-3.33	Sensitive
FOSL2	-265.92	-122.09	913	67.91	-5.07	Sensitive
FOXJ2	-303.38	-101.19	1195	64.44	-4.60	Sensitive
FOXP4*	-6.9	-6.39	62	56.45	-3.16	Sensitive
	-178.92	-45.95	508	79.53		
FYN*	-191.84	-96.63	565	72.39	-6.58	Sensitive
	-61.54	-10.74	272	55.51		
	0	0	61	39.34		
GBP2*	-70.31	-28.55	319	47.96	-2.26	Sensitive
	-109.35	-54.44	888	48.42		
GINS1	-4	-8.61	144	61.11	-3.57	Sensitive
GNAI1	-83.3	-48.06	423	72.10	-5.49	Sensitive
GNAO1	-63.2	-22.81	310	64.19	-2.86	Sensitive
GNB1*	-24.32	-16.22	203	53.69	-2.59	Sensitive
	-56.28	-18.04	382	68.85		
GNG12	-38.3	-15.76	250	56.40	-4.13	Sensitive
GPBP1L1*	-176.8	-87	1272	41.59	-9.76	Sensitive
	-196.68	-68.83	1411	43.59		
GPR137	-41.4	-17.56	155	62.58	-2.08	Sensitive
GPR161*	-14.8	-14.12	129	58.14	-2.65	Sensitive
	-80.8	-42.74	359	58.22		
	-163	-46.57	607	58.65		
	-76.72	-27.33	364	60.99		
GPRASP2*	-126.32	-64.59	670	52.09	-4.93	Sensitive
	-222.53	-88.79	969	53.04		
	-187	-89.67	881	53.46		
GPRIN1	-68.31	-29.56	228	73.25	-2.71	Sensitive
GRB10*	-2.05	-2.38	81	46.91	-3.76	Sensitive
	-112.56	-6.78	520	62.31		
GRK5	-24.2	-17.04	379	69.39	-3.92	Sensitive
GSK3B_001	-34.05	-14.66	282	41.84	-76.39	Sensitive

GSK3B_002	-34.05	-14.66	282	41.84	-11.39	Sensitive
H1F0	-92.91	-36.61	488	70.08	-6.64	Sensitive
H2AFV	-23.8	-6.83	142	80.99	-2.71	Sensitive
H2AFY*	-184.52	-98.11	644	71.58	-4.13	Sensitive
	-19.4	-9.79	182	55.49		
HCFC1	-300.55	-197.79	1017	65.09	-7.94	Sensitive
HEXIM1	-364.36	-174.07	1924	54.83	-6.28	Sensitive
HNRNPC*	-83.74	-34.39	290	54.48	-4.04	Sensitive
	-66.33	-21.89	571	50.61		
	-43.4	-14.28	214	53.27		
	-37.41	-21.34	240	50.83		
HNRNPD	-66.4	-45.76	328	68.60	-6.30	Sensitive
HNRNPH3	-39.2	-15.04	215	53.02	-4.08	Sensitive
HNRPLL	-93.34	-72.12	390	72.82	-3.59	Sensitive
HOMER1	-338.1	-168.78	1493	59.81	-11.72	Sensitive
HPS4	-65.82	-44.27	350	42.57	-6.50	Sensitive
HS2ST1	-126.83	-78.93	408	69.12	-4.12	Sensitive
IFT27 (RABL4)	-104.55	-70.52	496	66.94	-2.46	Sensitive
IGF1R	-181.18	-52.92	661	64.60	-3.12	Sensitive
IPO8	-88.35	-17.27	389	65.04	-4.34	Sensitive
JAK2*	-159.81	-49.63	544	66.73	-4.96	Sensitive
	-31.35	-16.64	163	57.67		
JOSD1	-89.8	-51.84	730	52.33	-5.39	Sensitive
JRK	-169.64	-72.14	564	63.48	-3.93	Sensitive
KANK2	-28.08	-7.93	129	58.91	-3.15	Sensitive
KIAA0430	-10.1	-4.79	257	60.70	-3.79	Sensitive
KIAA0494	-303.39	-159.21	1027	61.05	-2.70	Sensitive
KIAA0754	-22.59	0.64	245	45.71	-12.19	Sensitive
KIAA2018*	-152.94	-71.9	461	63.34	-4.97	Sensitive
	0	0	68	42.65		
KIF3C*	-118.4	-60.65	630	67.78	-2.82	Sensitive
	-247.3	-76.69	623	72.23		
KIF4A	-5	0	132	59.85	-3.55	Sensitive
KIF4B	-22.14	-16.72	155	55.48	-4.81	Sensitive
KPNB1	-86.71	-30.22	457	68.71	-3.70	Sensitive
LHFPL2*	-41.85	-8.75	361	59.00	-1.61	Sensitive
	-200.79	-102.16	726	63.77		
LMO4*	-244.33	-139.6	830	71.20	-4.78	Sensitive
	-28.88	-15.3	200	65.00		
LPHN2*	-5.71	-3.11	232	34.91	-2.11	Sensitive
	-32.01	-18.62	266	42.48		
LRP1	-148.85	-20.69	516	65.89	-2.42	Sensitive
LRRC1	-75.2	-25.55	327	74.01	-3.63	Sensitive
LRSAM1*	-127.54	-47.03	422	63.51	-3.85	Sensitive
	-123.62	-40.73	654	61.62		
	0	0	127	56.69		
	-170.1	-45.99	852	62.09		

LUZP1*	-17.1	-10.25	278	49.28	-3.98	Sensitive
	-79.74	-22.42	449	53.45		
MAML2*	-222.34	-99.85	1335	45.54	-4.10	Sensitive
	-169.52	-102.02	1113	46.36		
MAN1A2	-122.19	-59.46	785	54.27	-5.06	Sensitive
MAP2K1	-281.53	-133	581	73.67	-2.89	Sensitive
MAP3K11	-226.8	-93.31	536	69.78	-5.16	Sensitive
MAP3K3	-139.51	-66.39	370	80.00	-2.53	Sensitive
MAP4K4	-150.61	-50.3	387	73.90	-3.53	Sensitive
MAP7D1	-55.14	-29.19	266	78.57	-3.32	Sensitive
MAPK14	-145.09	-66.62	438	71.92	-2.91	Sensitive
MAPK9	-96.06	-35.86	321	73.83	-2.98	Sensitive
MAPKAP1*	-131.66	-59.24	383	64.49	-4.14	Sensitive
	-5.23	-7.03	119	42.02		
MARK1	-52.89	-5.26	316	74.68	-4.20	Sensitive
MBNL3	-11.51	-3.45	129	34.88	-2.78	Sensitive
MED13L	-100.7	-66.13	257	84.44	-5.91	Sensitive
MGAT2	-167.61	-90.59	548	70.07	-2.29	Sensitive
MIB1	-105.67	-93.32	314	78.98	-3.25	Sensitive
MID2	-139.5	-72.4	598	70.07	-3.49	Sensitive
MKKS*	-83.14	-57.86	813	41.45	-6.87	Sensitive
	-92.56	-37.64	937	38.63		
MLLT6	-13.88	-4.87	141	75.18	-3.57	Sensitive
MMP14	-40.74	-17.41	311	61.41	-2.82	Sensitive
MOB3A	-81.96	-21.57	368	60.60	-3.13	Sensitive
MORC2	-120.79	-67.68	1414	37.41	-8.08	Sensitive
MPP5	-163.23	-54.6	711	55.98	-5.16	Sensitive
MREG*	-118.74	-29.71	346	71.97	-2.19	Sensitive
	-115.47	-57.24	244	79.10		
MSI2	-36.53	-28.22	259	72.20	-2.36	Sensitive
MST4	-104.38	-52.45	303	68.65	-4.69	Sensitive
NCK2*	-87.7	-69.09	492	63.82	-3.79	Sensitive
	-10.55	-8.03	182	51.65		
NCOA1*	-30.61	-11	308	42.21	-7.17	Sensitive
	-120.69	-52.59	702	53.99		
NFE2L1	-107.86	-53.03	666	53.90	-4.30	Sensitive
NLRC5	-83.59	-19.86	275	60.36	-2.08	Sensitive
NOD1	-131.58	-60.04	576	53.65	-3.57	Sensitive
NOG	-72.47	-33.86	575	75.83	-3.43	Sensitive
NONO*	-50.83	-4.3	255	53.33	-4.40	Sensitive
	-33.82	-7.79	148	52.70		
	-49.69	-13.63	362	48.90		
NOTCH2	-75.02	-33.22	270	72.96	-2.95	Sensitive
NPAS2	-153.11	-69.14	335	75.22	-2.73	Sensitive
NR1H2	-84.54	-29.38	326	62.88	-3.10	Sensitive
NR2C2AP	-89.88	-42	396	58.08	-2.35	Sensitive
NR2F1	-369.06	-149.97	1737	64.25	-6.83	Sensitive

NRP2	-231.49	-96.85	841	55.17	-3.53	Sensitive
NSUN7	-92.66	-28.89	545	68.81	-3.79	Sensitive
NUCKS1	-76.41	-29.03	353	63.46	-3.85	Sensitive
NUDT4	-132.32	-92.18	477	74.00	-4.47	Sensitive
NUP62*	-115.48	-45.89	486	59.47	-3.35	Sensitive
	-85.16	-28.93	307	61.89		
	-67.94	-40.62	331	62.84		
NXPE3 (FAM55C)*	-117.62	-46.67	642	47.82	-4.67	Sensitive
	-26.74	-6.32	247	42.91		
	-66.61	-33.84	951	36.49		
	-84.98	-40.3	1006	52.49		
NXT1	-117.47	-27.11	437	69.57	-4.35	Sensitive
ODC1*	-136.44	-28.78	561	67.02	-3.16	Sensitive
	-73.95	-35.29	484	49.38		
OSBPL2	-58.81	-34.18	252	53.97	-3.30	Sensitive
OTUD4	-32.89	-19.12	188	48.40	-5.08	Sensitive
OXNAD1	-118.31	-75.16	515	53.20	-4.60	Sensitive
PABPC4	-154.22	-87.52	500	64.80	-3.38	Sensitive
PAFAH1B1	-138.72	-56.27	501	57.09	-15.50	Sensitive
PDE6D	-75.4	-33.71	245	68.57	-2.58	Sensitive
PDE7B	-25.61	-5.66	353	42.21	-3.01	Sensitive
PDXK	-40.95	-37.49	233	77.68	-2.80	Sensitive
PDZD2*	-61.87	-31.87	375	56.80	-5.51	Sensitive
	-128.2	-50.25	438	58.45		
PEG10	-144.24	-63.78	529	58.98	-4.72	Sensitive
PIGC*	-45.77	-10.59	384	51.30	-2.51	Sensitive
	-36.83	-4.14	331	50.76		
	-161.33	-60.46	1514	45.51		
PIGN*	-36.89	-17.93	498	46.59	-2.35	Sensitive
	-51.82	-22.52	421	47.98		
PIK3CA	-60.2	-10.75	207	56.04	-3.61	Sensitive
PIK3R3*	-176.41	-81.28	740	54.73	-4.30	Sensitive
	-46.17	-21.05	307	61.24		
	-31.59	-8.89	150	66.67		
PITPNA	-79.87	-57.98	306	80.39	-4.20	Sensitive
PLEKHA4	-60.95	-27.23	601	52.91	-65.86	Sensitive
PLXNA2	-228.56	-62.77	808	67.33	-6.01	Sensitive
PNMA2	-164.9	-67.44	945	57.99	-3.94	Sensitive
PPP1CC	-61.13	-27.07	241	66.80	-4.83	Sensitive
PPP1R3D	-91.6	-57.58	416	62.98	-6.01	Sensitive
PPP2R5A*	-189.58	-59.14	624	76.44	-3.73	Sensitive
	-124.56	-92.96	439	75.40		
PPP2R5B*	-193.17	-100.45	672	70.54	-3.17	Sensitive
	-160	-69.36	646	69.20		
PPP4C*	-64.99	-39.51	218	75.69	-3.39	Sensitive
	-77.31	-49.59	203	72.41		
PPP6R1 (SAPS1)	-101.42	-24.91	292	78.42	-3.05	Sensitive

PRMT2*	-235.33	-101.45	812	61.58	-2.84	Sensitive
	-69.2	-45.84	255	58.82		
	-175.74	-56.77	1004	57.27		
PSEN2	-132.68	-23.96	486	57.41	-2.12	Sensitive
PSME3	-91.42	-47.26	276	69.57	-2.70	Sensitive
PTER*	-15.5	-0.67	103	38.83	-4.23	Sensitive
	-28.39	-11.31	228	46.49		
	-20.7	-11.08	174	48.85		
	-43.64	-23.68	296	52.70		
PTPN11	-76.78	-53.83	248	77.42	-3.94	Sensitive
PTPN21*	-22.56	-2.09	255	58.43	-4.45	Sensitive
	-82.93	-44.02	335	65.37		
PTPN9	-185.39	-91.3	563	68.21	-2.37	Sensitive
PUM2*	0	0	73	39.73	-4.29	Sensitive
	-31.43	-11.02	174	60.92		
QKI	-148.71	-91.81	601	79.03	-6.58	Sensitive
RAB10	-68.64	-40.66	549	68.31	-4.27	Sensitive
RAB24*	-140.14	-50.22	470	70.43	-2.55	Sensitive
	-77.95	-13.16	382	70.16		
RAB27A*	-55.19	-23.82	280	50.00	-2.74	Sensitive
	-47.68	-19.83	302	36.42		
	-23.7	-9.39	243	39.51		
	-43.1	-22.95	283	39.22		
RAB9B	-65.43	-29.56	335	52.24	-3.95	Sensitive
RAD17*	-140.85	-92.8	728	47.25	-8.76	Sensitive
	0	0	112	32.14		
RAD51	-48.36	-33.37	318	65.09	-3.19	Sensitive
RANBP1	-17.52	-10.89	152	80.26	-1.85	Sensitive
RBBP7	-126.06	-63.14	411	68.86	-4.90	Sensitive
RFX5*	-45.9	-23.23	229	51.97	-2.78	Sensitive
	-36.25	-25.9	301	51.50		
	-43.95	-28.99	328	52.74		
RNF10*	-115.2	-41.83	511	72.41	-3.19	Sensitive
	-114.69	-32.77	459	71.24		
RNF4*	-101.82	-25.05	529	51.80	-7.41	Sensitive
	-315.94	-154.97	1565	45.37		
	-219.96	-106.66	920	56.74		
ROCK1	-313.52	-156.76	991	67.51	-4.67	Sensitive
RTN4IP1	-42.76	-47.48	516	50.00	-4.91	Sensitive
SAMD12	-49.87	-30.77	187	55.61	-6.78	Sensitive
SAMD4B	-176.67	-88.17	1085	55.39	-4.02	Sensitive
SAMD9L*	-215.78	-90.09	1267	42.54	-3.76	Sensitive
	-156.56	-44.55	868	45.16		
	-93.99	-34	672	43.30		
SCYL2	-130.8	-76.36	487	64.89	-4.81	Sensitive
SDC1*	-66.19	-14.15	295	73.22	-2.60	Sensitive
	-98.95	-63.82	441	75.06		

SDC2*	-296.21	-106.42	971	63.34	-2.29	Sensitive
	-132.99	-69.25	469	65.03		
SENP3	-62.79	-16.97	370	72.16	-2.98	Sensitive
SET	-123.74	-61.97	407	76.41	-4.52	Sensitive
SETD3	-80.9	-36.14	210	69.52	-3.94	Sensitive
SEZ6L2	-122.59	-43.96	578	62.11	-2.79	Sensitive
SGMS1	-119.11	-89.36	1010	53.56	-3.36	Sensitive
SGMS2*	-31.2	-14.08	439	37.81	-3.46	Sensitive
	-89.88	-34.02	607	49.26		
	-99.06	-57.11	679	41.83		
SH2D5	-123.38	-70.04	448	72.54	-2.29	Sensitive
SH3BP5L	-312.79	-153.71	1280	57.89	-4.18	Sensitive
SHB	-187.44	-118.81	616	75.32	-3.81	Sensitive
SIPA1L1	-38.65	-9.8	398	38.19	-5.04	Sensitive
SLC41A1	-330.32	-145.9	1065	60.85	-3.53	Sensitive
SLC45A3	-103.84	-10.69	346	66.47	-2.22	Sensitive
SLIT2	-117.34	-57.74	302	62.25	-2.92	Sensitive
SMAD3*	-128.4	-64.27	360	78.33	-3.10	Sensitive
	-83.88	-48.37	273	75.82		
SMAP2	-187.01	-107.95	474	73.42	-2.22	Sensitive
SMARCD3	-172.51	-107.67	461	68.98	-4.20	Sensitive
SMEK2	-93.29	-63.28	378	63.76	-6.01	Sensitive
SMURF2	-125.4	-30.37	238	80.25	-6.70	Sensitive
SNX33	-299.23	-188.56	1247	59.90	-6.06	Sensitive
SOX4*	-126.5	-71.7	844	56.28	-5.30	Sensitive
	-58.34	-20.68	664	54.22		
SPHK1	-185.2	-105.37	547	71.85	no data	Sensitive
SPIN1*	-36.8	-38.92	328	56.10	-3.79	Sensitive
	-137.2	-25.62	706	50.85		
SPTBN2*	-14.35	-9.61	122	61.48	no data	Sensitive
	-14.35	-9.61	122	61.48		
	-29.66	-24.17	382	60.73		
	-29.66	-24.17	382	60.73		
SRC*	-21.77	-7.85	152	63.16	-2.44	Sensitive
	-126.4	-51.19	399	65.41		
	-22.4	-8.77	89	57.30		
	-150.33	-67.68	503	59.24		
ST3GAL1*	-202.98	-114.63	1078	52.04	-3.25	Sensitive
	-13.52	-30.95	427	50.35		
	-177.82	-96.6	1067	52.11		
	-128.66	-59.94	788	51.40		
STAT6*	-65.1	-46.09	376	56.12	-2.45	Sensitive
	-64.4	-39.34	336	56.55		
	-48.02	-16.86	199	64.82		
	-58.4	-29.25	301	57.14		
STK11	-404.13	-207.7	1165	73.99	-4.46	Sensitive
STXBP5	-51.91	-13.24	158	70.89	-4.87	Sensitive

SYTL4*	-48	-1.32	238	51.68	-6.05	Sensitive
	-57.61	-28.16	237	51.48		
	-82.1	-39.03	397	45.84		
	-45.81	-7.16	382	49.48		
TAB3 (MAP3K7IP3)	-76.53	-15.18	597	43.55	-4.38	Sensitive
	-16.57	-9.82	182	39.01		
	-35.01	-16.59	228	45.18		
TAF4B	-238.03	-46.91	1048	61.74	-3.83	Sensitive
TAF6*	-133.85	-56.58	576	60.94	-2.86	Sensitive
	-117.6	-60.41	304	64.80		
	-45.9	-5.71	145	64.14		
TAF7	-96.22	-52.69	769	49.02	-4.42	Sensitive
TAOK1	-133.01	-56.62	569	68.19	-8.31	Sensitive
TAOK2	-385.55	-212.64	1093	66.15	-4.73	Sensitive
TATDN2*	-212.41	-91.84	1101	62.49	-2.30	Sensitive
	-19.75	-3.6	212	53.77		
TBC1D5*	-238.88	-122.18	1715	42.74	-7.47	Sensitive
	-6.28	3.76	227	50.22		
TBC1D8B	-12.91	-4.18	224	57.14	-3.13	Sensitive
TBL1X*	-153.64	-143.29	690	72.90	-2.41	Sensitive
	-63.32	-37.97	418	54.07		
TCEAL8*	-18.1	-10.25	196	41.33	-4.33	Sensitive
	-33.45	-14.03	287	44.25		
TCF12*	-65.03	-27.15	285	65.26	-5.16	Sensitive
	-81.44	-37.24	346	66.18		
TEAD1*	-38.65	-32.25	204	52.94	-4.81	Sensitive
	-101.83	-68.39	501	64.87		
TFPT	-75.38	-44.47	456	57.46	-2.79	Sensitive
TGFB1	-244.93	-117.73	932	71.78	-2.12	Sensitive
TIMM21	-19.7	-8.86	348	47.41	-4.70	Sensitive
TLE1	-340.85	-132.35	1115	70.22	-8.78	Sensitive
TMEM184C	-94.37	-57.84	624	56.73	-4.20	Sensitive
TMEM56	-63.28	-30.47	341	69.79	-2.89	Sensitive
TMEM65	-161.32	-109.84	585	72.99	-5.35	Sensitive
TMEM99	-50.27	-23.14	354	49.72	-2.89	Sensitive
TOR1AIP2_001	-78.06	-41.11	438	50.46	-4.69	Sensitive
TOR1AIP2_002	-78.06	-41.11	438	50.46	-2.60	Sensitive
TPX2	-62.05	-32.9	578	53.98	-5.13	Sensitive
TRAF3*	-70.2	-34.27	325	62.77	-3.48	Sensitive
	-76.86	-41.98	267	74.53		
TRIM37	-141.25	-58.84	510	68.04	-3.27	Sensitive
TRIM44	-104.97	-56.59	357	76.47	-4.19	Sensitive
TRIM8	-78.9	-37.04	173	74.57	-3.03	Sensitive
TRMT2B*	-123.46	-66.45	417	61.87	-2.89	Sensitive
	-269.84	-76.35	823	59.05		
	-66.07	-26.36	406	58.87		
TSC22D4	-248.84	-73.19	805	66.96	-5.91	Sensitive

TTLL5	-37.23	-6.93	255	59.22	-4.42	Sensitive
TYK2	-166.67	-60	532	60.53	-2.77	Sensitive
UBASH3B	-192.8	-95.19	425	67.76	-2.64	Sensitive
UBE2D2*	-14.7	-9.33	116	75.00	-3.89	Sensitive
	-88.49	-47.42	676	74.56		
UBE2E3*	-110.2	-50.5	268	72.39	-2.26	Sensitive
	-67.53	-38.52	434	68.66		
UBE2H*	-152.9	-96.6	444	75.45	-4.32	Sensitive
	-152.9	-96.6	444	75.45		
UBE2M	-229.05	-100.24	645	76.28	-2.55	Sensitive
UBE4B	-30.31	-10.8	125	56.00	-2.85	Sensitive
UBL3	-312.42	-184.48	1196	63.46	-8.35	Sensitive
UBQLN2	-111.91	-20.73	331	73.11	-3.25	Sensitive
UCP2	-67	-45.86	893	62.04	-3.39	Sensitive
USP9X	-157.16	-75.5	683	62.08	-4.27	Sensitive
VANGL1*	-62.3	-37.28	265	49.43	-2.26	Sensitive
	-48.6	-36.89	321	57.63		
	-6.8	-0.27	62	38.71		
VEPH1*	-72.41	-17.07	367	48.50	-6.68	Sensitive
	-53.26	-15.41	358	46.93		
WDR12	-119.17	-46.91	800	46.38	-2.65	Sensitive
WDR24	-153.55	-81.65	810	63.70	-3.97	Sensitive
WDTC1	-92.21	-36.22	433	63.28	-4.23	Sensitive
WHSC1L1	-193.12	-87.62	568	69.19	-4.85	Sensitive
WLS	-84.42	-40.05	304	58.22	-1.63	Sensitive
WNK1	-245.3	-51.88	693	75.90	-5.09	Sensitive
WNK3	-42.46	-50.58	489	56.24	-4.85	Sensitive
WWC1	-0.76	0	53	77.36	-3.09	Sensitive
XPO1*	-57.61	-15.17	193	57.51	-8.06	Sensitive
	-121.82	-125.15	778	59.25		
	-74.11	-49.04	457	52.30		
XPO6	-188.24	-118.5	551	72.05	-4.14	Sensitive
XRCC3*	-36.7	-34.67	391	54.73	-4.11	Sensitive
	-68.92	-38.56	451	59.20		
	-36.2	-15.74	359	52.92		
	-55.1	-18.26	413	56.17		
	-209.42	-107.45	847	58.32		
XRRA1	-80.61	-45.59	382	59.95	-2.93	Sensitive
YAP1	-185.39	-78.18	438	73.06	-6.07	Sensitive
YWHAH	-35.19	-6.02	323	79.57	-3.63	Sensitive
ZDHHC5	-228.8	-157.35	1412	49.93	-4.42	Sensitive
ZFP91	-68.91	-25.46	221	76.92	-4.41	Sensitive
ZFYVE1	-117.21	-70.65	771	51.49	-3.98	Sensitive
ZFYVE20*	-147.7	-50.12	664	51.51	-3.42	Sensitive
	-141	-71.4	523	53.92		
ZHX1*	-68.01	-53.79	575	49.91	-4.98	Sensitive
	-84.9	-37.59	668	54.34		

	-101.27	-45.29	591	46.19		
ZHX3*	-10.62	-14.41	428	44.39	-4.82	Sensitive
	-19.48	-22.57	410	43.90		
	-59.99	-7.04	399	54.14		
	-79.62	-43.82	955	45.65		
ZMAT3	-264.53	-146.24	792	65.03	-1.84	Sensitive
ZNF45	-187.17	-89.68	1141	51.80	-8.19	Sensitive
ZNF521*	-46.61	-25.96	248	51.61	-4.13	Sensitive
	-56.4	-31.41	199	54.77		
ZNF528	-54.4	-14.66	476	45.59	-4.50	Sensitive
ZNF664*	-173.58	-64.51	999	49.65	-14.59	Sensitive
	-469.85	-209.05	1880	59.89		
	-160.92	-65.16	1076	51.67		
	-147.72	-82.64	1030	50.58		
ZNF672	-199.69	-95.06	796	63.07	-4.05	Sensitive
ZNF740	-104.56	-28.54	495	53.33	-4.40	Sensitive
ACACA	-63.5	-15.73	482	62.03	1.12	Insensitive
ACIN1	-50.6	-21.62	328	54.57	-1.16	Insensitive
ACSS3	-153.73	-77.81	910	51.10	1.08	Insensitive
ADAMTSL1	-33.42	-17.58	339	53.69	1.20	Insensitive
ADAT3	-36.17	-14.71	268	69.40	-1.10	Insensitive
AKAP10	-69.82	-25.79	158	67.72	-1.17	Insensitive
AKIRIN1	-19.15	-18.78	158	74.68	1.16	Insensitive
ALDH6A1	-18.73	-4.75	99	68.69	1.19	Insensitive
AMDHD2	-1.9	-2.05	69	75.36	1.16	Insensitive
AMMECR1	-69.95	-44.31	168	78.57	-1.14	Insensitive
ANAPC7	0	0	1	100.00	1.04	Insensitive
ANKRD44	-55.36	-25.18	183	66.12	1.14	Insensitive
ANKRD52	-59.9	-30.84	122	83.61	1.06	Insensitive
ANXA7	-16.1	0	57	68.42	1.00	Insensitive
AP1S3	-30.34	-9.23	132	71.21	-1.02	Insensitive
AP5Z1	-24.9	-7.46	94	70.21	1.15	Insensitive
ARHGAP17	-14.2	-9.07	70	84.29	-1.01	Insensitive
ARRDC2	-26	-12.6	143	65.73	1.04	Insensitive
ASB8	-16.81	-5.33	170	58.24	-1.09	Insensitive
ATP6V0A2	-73.28	-51.62	248	79.84	1.06	Insensitive
ATPAF1	-30.21	-8.49	105	84.76	1.04	Insensitive
AVEN	-18.7	-9.85	130	80.00	-1.08	Insensitive
BBS2	-87.61	-38.38	421	66.51	-1.05	Insensitive
BCAR1	-27.16	0	127	85.83	-1.07	Insensitive
BCKDHB	-7.7	-7.53	47	70.21	1.17	Insensitive
BFSP1	-14.82	-9.82	40	90.00	1.16	Insensitive
BNIP3L	-77.5	-33.92	233	70.82	1.07	Insensitive
BOD1	-19.61	-17.74	224	68.75	1.01	Insensitive
BRE	-38.8	-8.61	138	63.04	1.06	Insensitive
BTRC	-25.28	-14.59	118	72.03	-1.00	Insensitive
C11orf1	-56.77	-23.7	337	55.49	-1.04	Insensitive

C15orf61	-88.02	-24.31	181	83.43	1.16	Insensitive
C19orf12	-12.4	-20.73	127	66.93	1.11	Insensitive
C9orf156	-3.5	-3.08	77	62.34	1.19	Insensitive
CARKD	0	0	14	71.43	-1.03	Insensitive
CARS2	-19.1	-4.07	64	75.00	1.12	Insensitive
CASP2	-106.3	-51.27	241	72.20	1.08	Insensitive
CASP6	-34.47	-17.9	78	71.79	-1.13	Insensitive
CAT	-19.6	-8.82	89	62.92	1.14	Insensitive
CCDC137	-98.85	-15.39	403	73.95	-1.11	Insensitive
CCDC6	-74.5	-9.31	232	71.98	1.09	Insensitive
CCDC71	-40.8	-18.08	107	70.09	1.16	Insensitive
CCDC75	-17.8	-4.54	145	60.00	-1.06	Insensitive
CCDC90A	-17.77	-7.63	139	75.54	1.15	Insensitive
CCDC94	-4.1	-5.37	68	48.53	1.15	Insensitive
CCNO	-60.4	-33.16	157	68.15	1.01	Insensitive
CDK2AP2	-177.94	-73.41	549	71.58	1.15	Insensitive
CDK5RAP1	-59	-29.71	154	63.64	-1.01	Insensitive
CDKAL1	-6.71	-3.21	167	44.31	-1.10	Insensitive
CEP72	-17.45	-6.94	90	74.44	-1.18	Insensitive
CEP85	2.44	0	131	59.54	1.01	Insensitive
CHAC1	-88.52	-30.73	309	68.61	1.07	Insensitive
CHCHD1	0	-0.48	13	61.54	1.03	Insensitive
CIZ1	-34.71	0.52	163	79.75	1.05	Insensitive
CLMN	-59.8	-35.43	114	85.09	1.04	Insensitive
CTNNBL1	-34.6	-24.99	117	68.38	-1.02	Insensitive
CUL4A	-54.56	-41.87	249	66.67	-1.08	Insensitive
CUL4B	-74.76	-20.7	402	46.52	-1.08	Insensitive
CWF19L1	-22.61	-10.92	87	54.02	1.01	Insensitive
CYTH2	-121.73	-46.48	300	66.67	1.17	Insensitive
DCAF10	-119.31	-30.69	365	67.67	-1.03	Insensitive
DCAF15	0	0	21	57.14	-1.01	Insensitive
DCP1B	-7.3	-5.73	80	60.00	1.07	Insensitive
DCTN1	-99.89	-54.79	318	61.32	1.05	Insensitive
DDB2	-31.38	-17.9	195	60.51	1.08	Insensitive
DDIT4	-26.81	-28.45	201	65.67	1.10	Insensitive
DDX24	-18.13	-15.44	132	65.15	1.13	Insensitive
DDX47	0	0	22	54.55	-1.03	Insensitive
DERA	-82.6	-7.65	212	73.58	1.17	Insensitive
DHTKD1	-18.7	-8.22	62	69.35	1.02	Insensitive
DHX15	-26.15	-9.19	157	59.87	-1.09	Insensitive
DHX30	-129.4	-83.85	407	66.58	1.04	Insensitive
DHX40	-20.35	-9.59	147	63.27	-1.01	Insensitive
DIS3L	-16.8	-9.66	50	84.00	-1.16	Insensitive
DMAP1	-3.46	-0.23	78	73.08	1.12	Insensitive
DNAJB2	-48.34	-41.45	288	74.65	1.11	Insensitive
DOCK2	-18.86	-9.8	80	77.50	1.18	Insensitive
DOCK7	0	0	18	83.33	1.00	Insensitive

DPY19L3	-63.7	-22.53	215	66.51	1.03	Insensitive
DSP	-23.8	-21.18	341	73.61	-1.18	Insensitive
DTD1	-31.07	-24.64	180	77.22	1.02	Insensitive
ECI2	0	0	37	78.38	-1.05	Insensitive
EDC4	-51.1	-28.48	239	74.90	1.01	Insensitive
EDF1	-5.4	2.78	28	78.57	1.14	Insensitive
EHHADH	-15.4	-2.57	76	65.79	-1.06	Insensitive
ELP3	-68.61	-17.76	377	57.82	1.07	Insensitive
ELP4	-4.44	-1.51	35	45.71	1.09	Insensitive
ERCC8	-6.7	-1.83	43	62.79	-1.08	Insensitive
FAM120B	1.34	3.18	108	66.67	1.06	Insensitive
FAM63B	-28.4	-20.37	82	68.29	1.12	Insensitive
FIG4	-27.3	-16.32	124	65.32	1.13	Insensitive
GABARAPL2	-9.2	0	136	75.00	1.18	Insensitive
GDI2	-51.62	-39.58	291	69.76	1.03	Insensitive
GID4	-41.7	-27.14	173	65.32	1.16	Insensitive
GINS3	-30.17	-27.72	208	60.10	1.10	Insensitive
GLIPR1	-32.66	-8.8	201	49.75	1.14	Insensitive
GLYR1	-22.1	-12.53	77	74.03	1.08	Insensitive
GMPPB	-95.9	-44.4	225	73.78	1.02	Insensitive
GNB5	-9.6	-6.49	66	51.52	1.20	Insensitive
GOLGA2	0	0	13	76.92	1.05	Insensitive
GPD1L	-67.65	-35.34	201	76.12	-1.08	Insensitive
GPKOW	-18.87	-11.66	79	58.23	1.02	Insensitive
GPR126	-65.65	-11.22	401	69.08	1.06	Insensitive
HARS2	-42.9	-19.86	223	61.43	1.02	Insensitive
HEATR5A	-19.51	-20.74	185	53.51	1.19	Insensitive
HES4	-53.96	-0.84	138	84.06	1.09	Insensitive
HIF1AN	-5.7	-8.48	100	56.00	1.11	Insensitive
HIST1H2AA	-9.4	-6.39	35	51.43	1.04	Insensitive
HSBP1L1	-28.61	-18.5	175	78.86	1.10	Insensitive
HTT	-55.2	-22.27	145	73.10	1.08	Insensitive
IDH3A	-19.3	0	83	75.90	-1.11	Insensitive
IFT172	-31.5	-20.88	104	60.58	1.07	Insensitive
ILF2	-14.11	-10.21	126	59.52	1.01	Insensitive
INTS2	-19.21	-13.09	76	63.16	-1.03	Insensitive
ITPKC	-10.1	-5.34	33	75.76	1.02	Insensitive
KANSL3	-16.4	-9.77	77	63.64	1.11	Insensitive
KAT5	-59.92	-42.95	234	65.81	1.07	Insensitive
KBTBD4	-8.1	0	64	67.19	-1.01	Insensitive
KDM4C	-142.8	-73.42	565	64.96	-1.12	Insensitive
KDM8	0.41	-1.74	118	59.32	-1.04	Insensitive
KIAA1109	0	0	45	31.11	1.07	Insensitive
KIAA1328	0	-1.22	22	59.09	1.18	Insensitive
KIAA1549	-16.2	-10.39	49	85.71	-1.02	Insensitive
KLHL5	-27.35	-18.84	283	39.22	-1.02	Insensitive
KRBA1	-166.59	-68.22	399	66.17	-1.19	Insensitive

LEMD3	0	0	26	42.31	1.06	Insensitive
LGALS3	-106.4	-69.81	261	78.16	1.10	Insensitive
LIMS2	-25.48	-15.7	158	77.85	1.10	Insensitive
LIPE	-31.16	-10.46	277	49.46	-1.01	Insensitive
LRIG3	-58.12	-28.45	287	64.46	-1.06	Insensitive
LTA4H	0	2.51	142	57.75	1.10	Insensitive
MAN2C1	-11.1	-6.76	47	70.21	-1.12	Insensitive
MAP2K4	-24.7	-11.82	63	69.84	1.18	Insensitive
MAP3K12	-39.07	-9.52	226	69.47	-1.12	Insensitive
MAVS	-18.47	-27.97	128	67.97	1.00	Insensitive
MBLAC2	-102.59	-39.78	476	69.54	1.13	Insensitive
MCCC2	-35.68	-3.67	129	68.22	1.00	Insensitive
MCM4	-89.72	-22.8	209	73.68	1.04	Insensitive
MCM6	-6.23	0	61	57.38	1.01	Insensitive
MED19	0	-2.05	22	81.82	1.18	Insensitive
METAP1	-33.59	-14.04	134	76.87	-1.04	Insensitive
MLXIP	-24.71	-1.67	132	84.09	1.13	Insensitive
MPLKIP	-11.7	-6.97	92	57.61	1.10	Insensitive
MRPL19	2.4	-0.18	25	56.00	-1.03	Insensitive
MRPL46	-7.3	-2.08	42	61.90	1.08	Insensitive
MRPS28	0	0	23	43.48	1.10	Insensitive
MRPS5	-96.3	-43.46	209	75.12	1.14	Insensitive
MSN	-30.2	-5.06	172	61.63	-1.08	Insensitive
MSRA	-40.86	-32.24	249	78.31	1.11	Insensitive
MTA1	-46.1	-5.7	214	85.05	1.06	Insensitive
MTA3	-65.8	-13.39	229	79.04	1.12	Insensitive
MTG1	-14.15	-5.17	50	76.00	1.15	Insensitive
MTHFD1	-132.07	-60.64	387	59.69	-1.15	Insensitive
MTRR	0	0	30	56.67	1.06	Insensitive
MUM1	-22.13	-11.67	101	64.36	-1.07	Insensitive
MUS81	-105.16	-35.75	349	67.91	-1.09	Insensitive
MUTYH	-45.7	-15.15	111	72.07	1.03	Insensitive
MYO18A	-64.57	-28.11	181	68.51	1.16	Insensitive
N6AMT1	-0.9	0	26	61.54	1.19	Insensitive
NAA40	-15.43	-10.97	101	69.31	-1.00	Insensitive
NBR1	-15.2	0	83	73.49	1.11	Insensitive
NDNL2	-35.75	-22.57	124	74.19	1.01	Insensitive
NDOR1	-2.3	0	83	66.27	-1.08	Insensitive
NFATC2IP	0	0	75	66.67	-1.06	Insensitive
NLN	-71.8	-36.05	178	79.78	1.01	Insensitive
NOP2	-144.97	-70.16	489	63.80	-1.01	Insensitive
NOP56	-118.35	-64.75	516	73.06	-1.02	Insensitive
NPRL3	0.26	-13.75	403	77.42	1.16	Insensitive
NSFL1C	-231	-116.14	868	60.02	1.14	Insensitive
NSMCE4A	-12.4	-6.85	52	57.69	-1.15	Insensitive
NT5C2	-11	-11.65	88	56.82	1.12	Insensitive
NUB1	-3.46	-4.65	77	71.43	1.06	Insensitive

NUDT21	-35.34	-15.36	173	65.90	1.11	Insensitive
NUP214	-48.65	-23.99	144	68.75	-1.12	Insensitive
OCRL	-50.61	-23.78	165	75.15	1.06	Insensitive
ODF2	-40.97	-14.53	585	58.63	-1.15	Insensitive
OGFOD2	-122.31	-55.17	739	61.57	-1.05	Insensitive
OSBP	-68.3	-50.79	480	63.75	1.04	Insensitive
PABPN1	-41.93	-10.32	181	60.77	1.03	Insensitive
PARP4	-21	-15.12	106	68.87	1.04	Insensitive
PDCD11	-20.59	0	94	64.89	-1.15	Insensitive
PDHB	-11.9	1.25	43	58.14	-1.01	Insensitive
PDIA4	-86.97	-20.69	233	75.54	1.05	Insensitive
PDLIM3	-11.6	0	74	78.38	1.04	Insensitive
PDLIM7	-26.4	-13.44	67	74.63	1.07	Insensitive
PEAR1	-25.91	-13.54	116	73.28	1.02	Insensitive
PHF13	-103.51	-48.43	382	75.65	1.14	Insensitive
PHKG2	-75.82	-44.93	239	69.87	1.02	Insensitive
PHLDB1	-75.19	-16.88	411	51.09	-1.03	Insensitive
PHLPP1	-69.35	-37.76	234	71.79	-1.08	Insensitive
PIK3C3	-1.72	0	86	51.16	1.04	Insensitive
PIR	-45.57	-20.33	204	64.71	1.20	Insensitive
PISD	-91.23	-38.26	420	60.71	1.11	Insensitive
PKNOX1	-39.82	-15.52	211	59.24	-1.04	Insensitive
PLOD2	-30	-10.99	178	78.09	-1.02	Insensitive
PLRG1	-27.1	-15.61	127	60.63	-1.04	Insensitive
PLSCR4	-68.08	-35.57	241	61.41	1.06	Insensitive
PMM2	-10.2	0.91	66	60.61	1.18	Insensitive
POLR1A	-11.17	-18.47	379	46.17	1.03	Insensitive
POLR1E	-0.6	2.04	113	66.37	1.07	Insensitive
POP5	-8.3	1.32	36	75.00	-1.03	Insensitive
POU2F2	-34.4	-10.29	67	73.13	1.02	Insensitive
PPAN	0	-0.22	27	66.67	1.04	Insensitive
PPP1R7*	-114.34	-28.29	474	66.24	1.13	Insensitive
	-101.19	-55.1	308	66.88		
PPP1R8	-30	-15.97	58	65.52	1.09	Insensitive
PRIM1	-2	-4.36	37	59.46	-1.10	Insensitive
PRR11	-75.58	-42.05	312	54.17	-1.09	Insensitive
PRRC2B	-4.5	-1.93	55	47.27	-1.04	Insensitive
PSMA1	-70.59	-18.64	397	60.71	-1.13	Insensitive
PSMC5	-49.91	-31.58	308	54.22	-1.16	Insensitive
PSMD11	-106.83	-47.53	263	72.24	1.19	Insensitive
PSMD9	-32.5	-9.7	126	69.05	-1.03	Insensitive
PSPC1	-12.5	-11.86	160	47.50	-1.17	Insensitive
PTCD3	-11.7	-4.47	66	56.06	-1.01	Insensitive
PTX3	-23.9	-8.56	145	51.72	-1.07	Insensitive
PWP1	-31.7	-22.08	168	63.10	1.15	Insensitive
RAB36	-3.3	0.14	40	67.50	-1.11	Insensitive
RBL2	-49	-10.45	137	77.37	1.19	Insensitive

REXO1	-47.45	-23.68	95	88.42	1.07	Insensitive
REXO4	-64.6	-30.07	200	74.00	-1.12	Insensitive
ROBO4	-67.37	-35.81	486	53.91	-1.02	Insensitive
RPAP1	-9.8	0	117	64.10	1.04	Insensitive
RRP12	-32.12	-14.23	112	53.57	-1.08	Insensitive
RRP1B	-1.96	-7.69	117	80.34	-1.20	Insensitive
RSPH3	-47.28	-4.96	190	68.42	1.16	Insensitive
RWDD1	-102.07	-49.33	216	71.30	-1.08	Insensitive
SAP30BP	-54.65	-32.82	257	59.14	-1.11	Insensitive
SERGEF	-34.6	-15.06	152	79.61	1.13	Insensitive
SF3A1	-31.7	-4.04	155	70.32	1.06	Insensitive
SF3B2	-16.4	-8.7	49	71.43	1.19	Insensitive
SGCB	-70.45	-32.77	223	77.13	1.13	Insensitive
SGPP1	-3.6	-24.28	95	75.79	1.05	Insensitive
SLBP	-181.04	-43.76	367	78.47	1.06	Insensitive
SLC25A28	-13.5	-6.19	29	86.21	-1.02	Insensitive
SLC25A37	-54.21	-30.33	198	72.73	1.13	Insensitive
SLC25A43	-133.2	-60.55	344	70.93	1.07	Insensitive
SLC35F3	-37.46	-18.29	145	73.79	1.12	Insensitive
SMARCA1	-27.08	-1.45	130	66.92	-1.19	Insensitive
SMG9	-64.17	-6.82	343	65.89	-1.16	Insensitive
SMYD4	-57	-24.71	168	75.00	1.01	Insensitive
SNTB2	-10	-5.67	38	71.05	1.14	Insensitive
SNUPN	-48.77	-23.71	283	57.95	-1.15	Insensitive
SNX1	0	0	14	64.29	-1.04	Insensitive
SNX6	-12.2	-6.37	31	80.65	1.14	Insensitive
SOD1	-39.87	-8.23	148	63.51	1.08	Insensitive
SP1	-22.33	-4.54	98	70.41	-1.01	Insensitive
SPAG7	-6.82	-4.86	68	58.82	1.14	Insensitive
SPECC1	0	0	51	43.14	-1.04	Insensitive
SRP68	-40.24	-26.08	162	73.46	1.07	Insensitive
SRSF7	-29.23	-11.84	238	60.08	-1.08	Insensitive
STK35	-114.36	-36.14	271	64.94	-1.12	Insensitive
STX18	-7.11	-3.4	64	76.56	1.09	Insensitive
STX1A	-2.7	0	27	85.19	1.17	Insensitive
TAMM41	-31.55	-5.14	283	65.72	-1.03	Insensitive
TBC1D1	-82.99	-46.15	355	67.61	-1.13	Insensitive
TCEB3	-99.28	-64.92	271	76.75	-1.02	Insensitive
TCP1	-87.95	-29.91	281	66.55	-1.05	Insensitive
TERF2IP	-34.7	-13.98	97	63.92	1.04	Insensitive
TEX2	-18.35	0	85	68.24	1.02	Insensitive
TMEM186	-7	-2.49	34	64.71	1.02	Insensitive
TMEM206	-68.2	-35.82	138	73.19	-1.03	Insensitive
TMEM9B	-206.59	-34.99	460	70.87	1.05	Insensitive
TMX3	-108.7	-60.74	317	65.30	1.19	Insensitive
TNS1	-117.27	-52.97	453	64.02	-1.18	Insensitive
TOMM5	-9.95	-12.18	64	73.44	-1.11	Insensitive

TOP1MT	-7	-4.12	35	68.57	-1.10	Insensitive
TRAPPC11	-51.43	-31.68	202	68.32	-1.13	Insensitive
TRMT1	-6.37	-4.57	129	61.24	-1.05	Insensitive
TTC39C	-98.1	-52.1	236	78.39	1.15	Insensitive
TTC8	-2.87	0	46	73.91	-1.09	Insensitive
TUFT1	-14.4	-7.9	62	67.74	1.18	Insensitive
TULP3	0	0	41	68.29	-1.15	Insensitive
TXNDC17	-68.79	-43.3	325	60.31	1.11	Insensitive
TXNRD1	-3.2	-0.55	24	62.50	-1.11	Insensitive
UBA2	-17.6	-11.6	70	80.00	-1.09	Insensitive
UBAP2L	-26.85	-14.53	167	55.69	-1.03	Insensitive
UBR4	0	0	28	64.29	1.14	Insensitive
UFC1	-29.68	-23.52	246	45.12	1.15	Insensitive
URGCP	-89.96	-19.57	494	64.17	-1.19	Insensitive
USP13	-47.8	-39.53	471	77.71	-1.16	Insensitive
USP46	-46.73	-22.78	185	71.89	-1.03	Insensitive
VIM	-65.22	-22.1	413	63.20	-1.13	Insensitive
WDR76	-17.2	-13.06	70	72.86	1.11	Insensitive
XPO5	-57.4	-16.04	211	74.88	-1.03	Insensitive
XRCC5	-83.68	-20.23	461	61.17	-1.07	Insensitive
ZBTB25	-91.35	-49.1	192	75.52	1.13	Insensitive
ZC3HC1	-14.7	-4.21	85	58.82	1.04	Insensitive
ZKSCAN3	-22.41	-18.82	216	57.41	-1.00	Insensitive
ZMIZ2	-60.4	-39.05	123	71.54	1.09	Insensitive
ZNF346	-10	-8	43	65.12	1.01	Insensitive
ZNF354C	-117.99	-67.05	306	71.57	-1.19	Insensitive
ZNF48	-85.01	-61.27	376	81.12	-1.05	Insensitive
ZNF668	-93.9	-55.38	684	57.60	-1.19	Insensitive
ZNF687	-55.8	-16.88	146	71.92	-1.06	Insensitive
ZYX	-163.74	-96.82	345	77.10	1.00	Insensitive

Table S5 | Table of vectors used in luciferase assays

vector name	5'UTR	parent vector
pMH2	N/A (CMV alone)	pGL4.25
pCR300	*CyclinD1*	pMH2
pCR301	*Rock1*	pMH2
pCR302	*Pfn2*	pMH2
pCR303	*Arf6*	pMH2
pCR304	*Arf6mut*	pMH2

Table S6 | Genes with reduced TE in the presence of INK128		
Gene name	log2(change in TE)	z-score (log2 (change in TE))
AASS	0.515372161	-2.0395347
AHCY	0.381309714	-2.401865638
AKT1S1	0.653703097	-1.665667305
ALDH9A1	0.429112605	-2.272668635
ASNS	0.595163021	-1.823883727
ATF4	0.685502922	-1.579721832
ATG7	0.460978503	-2.186544586
ATP5G3	-0.064317838	-3.606264427
ATPIF1	0.502521217	-2.074266981
BCL3	0.482912569	-2.127263327
BCORL1	0.610838431	-1.781517754
BHLHE40	0.593750062	-1.827702535
BRK1	0.527600913	-2.006484019
BTBD2	0.530887913	-1.997600235
CCNI	0.57492541	-1.878579973
CCNL1	0.228752408	-2.814182681
CDC5L	0.508880783	-2.057078965
CERK	0.448981958	-2.218967681
CIRBP	0.690557996	-1.56605947
COX6C	0.508414235	-2.058339905
CRTAP	0.254475902	-2.744659724
CSRNP1	0.38389789	-2.394870568
CTDSPL	0.664870654	-1.635484719
CUL2	0.700818061	-1.538329565
DENND1A	0.496502172	-2.09053467
DIP2A	0.697422896	-1.547505686
DLGAP4	0.698087521	-1.545709403
DMWD	0.425077475	-2.283574392
DNAJB1	0.637762038	-1.708751249
ECSIT	0.592881792	-1.830049211
EEF1A1	-0.081836105	-3.653611095
EEF1B2	0.40124211	-2.347994297
EEF1D	0.166259321	-2.983082916
EEF1G	-0.653979552	-5.199944735
EEF2	-0.034649952	-3.526080951
EGR1	-0.067876474	-3.615882362
EIF2S3	0.665826944	-1.632900151
EIF3E	0.500802989	-2.078910841
EIF3F	0.499692936	-2.081910984
EIF3H	0.18850202	-2.922967514
EIF3L	0.149025969	-3.029659543
EIF4B	0.528458273	-2.00416683
EXOC3	0.298924632	-2.624528022
FAM160B2	0.584498981	-1.852705457
FASN	0.339551358	-2.514726059
FTH1	-0.23187713	-4.059127378

FTL	-0.482845172	-4.737419384
G0S2	0.472929654	-2.154244178
GADD45A	0.51619605	-2.037307973
GADD45B	0.447799212	-2.222164292
GAMT	0.361140657	-2.456376603
GAPDH	0.521495419	-2.022985354
GLTSCR2	-0.282271408	-4.19532813
GLUL	0.479862971	-2.135505484
GNB2L1	0.23608559	-2.79436327
GSG2	-0.839007043	-5.700019035
HIST1H1C	0.690761053	-1.565510668
HIST1H1E	0.536462713	-1.982533208
HIST1H2AB	0.084054103	-3.205259181
HIST1H2AC	0.141741579	-3.049347084
HIST1H2AG	-0.143710514	-3.820839227
HIST1H2AH	0.148467388	-3.031169222
HIST1H2AI	0.202009418	-2.886461032
HIST1H2AJ	0.263397961	-2.720546051
HIST1H2AK	0.123703321	-3.098099132
HIST1H2AM	0.704435747	-1.528552035
HIST1H2BB	0.023022406	-3.370209714
HIST1H2BC	-0.124527712	-3.768993816
HIST1H2BD	0.032080481	-3.34572843
HIST1H2BE	0.0048643	-3.419285676
HIST1H2BJ	-0.022009033	-3.491916305
HIST1H2BK	0.005526469	-3.41749603
HIST1H2BL	-0.134517661	-3.795993678
HIST1H2BM	-0.284579931	-4.201567381
HIST1H2BN	0.043257137	-3.315521251
HIST1H2BO	0.086075273	-3.199796559
HIST1H3A	-0.007089192	-3.451592411
HIST1H3B	0.120455075	-3.106878176
HIST1H3C	0.18618935	-2.929217973
HIST1H3H	-0.158673708	-3.861280292
HIST1H3J	-0.131141283	-3.786868332
HIST1H4A	-0.022874327	-3.494254938
HIST1H4B	-0.101136414	-3.705774092
HIST1H4C	0.017922981	-3.383991943
HIST1H4H	0.127767249	-3.087115543
HIST1H4J	-0.061787609	-3.59942597
HIST1H4K	-0.090411681	-3.676788327
HIST2H2AB	0.578927694	-1.867762989
HIST2H2AC	0.469507436	-2.163493416
HIST2H2BE	0.135952249	-3.064993922
HIST2H2BF	0.095403083	-3.174586262
HIST2H3D	-0.138777005	-3.807505419
HIST4H4	0.101804121	-3.157286159
HMGA1	0.622346315	-1.750415365
HNRNPA1	0.407312141	-2.331588808

HNRNPH2	0.442148449	-2.237436624
HSPG2	0.608509473	-1.787812235
IER2	0.48752991	-2.114784027
IER3	0.377543704	-2.412044043
IFI16	0.708722419	-1.516966435
IFRD1	-0.201998716	-3.978374908
IGBP1	0.646955565	-1.683903878
IMPDH2	0.669181035	-1.623835041
JUNB	0.478030815	-2.140457257
KLF10	0.265737587	-2.714222738
LGALS1	0.66033614	-1.647740162
MAN2B1	0.640596831	-1.701089646
ME3	0.705006429	-1.527009651
MECOM	0.366454641	-2.442014484
MIDN	0.62847093	-1.733862351
MKLN1	0.599480156	-1.812215795
MPST	0.617218743	-1.764273668
MTA1	0.699371763	-1.542238478
MTMR12	0.147019402	-3.035082697
MTMR4	0.653231976	-1.666940605
NAP1L1	0.595638598	-1.822598384
NFKBIA	0.663634847	-1.638824738
NFKBIZ	0.078553631	-3.220125322
NOA1	0.622476906	-1.750062416
NTMT1	0.702174808	-1.534662681
NUAK2	0.517484928	-2.033824519
ODC1	0.671141203	-1.618537289
OSTC	0.53645977	-1.982541162
PABPC1	-0.856197574	-5.74647993
PCBP2	0.561050026	-1.916081011
PFDN5	0.390049765	-2.378243878
PNPLA6	0.566814611	-1.900501051
PRKCI	0.664776836	-1.635738281
PRPS2	0.389325027	-2.38020263
PRR11	0.566515319	-1.901309949
PSAT1	0.639474723	-1.70412237
PTEN	0.618051916	-1.762021849
QARS	0.541325208	-1.96939133
RAB13	0.641574365	-1.698447662
RABGGTB	0.350308436	-2.485652876
RASSF7	0.700975863	-1.537903073
RBBP4	0.599079809	-1.813297814
RNF130	0.705728941	-1.525056916
RPL10	0.085950646	-3.200133389
RPL10A	0.42569437	-2.281907108
RPL11	-0.455321888	-4.66303213
RPL12	-0.888154455	-5.832849878
RPL13	-0.13915362	-3.808523297
RPL13A	-0.34415006	-4.36256773

RPL14	0.465864032	-2.173340454
RPL15	0.060996083	-3.267578154
RPL18	0.147019791	-3.035081646
RPL18A	-0.556498233	-4.936481711
RPL19	0.504792863	-2.068127397
RPL22	0.160557898	-2.998492168
RPL23	-0.030828091	-3.515751597
RPL23A	0.091896455	-3.184063635
RPL24	0.675473743	-1.606827722
RPL26	-0.703112067	-5.332735316
RPL27	0.402928004	-2.343437827
RPL27A	-0.208171869	-3.995059105
RPL28	-0.354925074	-4.391689389
RPL29	-0.168506136	-3.887854422
RPL3	-0.291901526	-4.221355476
RPL30	0.019488821	-3.379759943
RPL31	-0.689485049	-5.295905538
RPL32	-0.031707175	-3.5181275
RPL34	-0.455485437	-4.663474154
RPL35	0.131784083	-3.076259235
RPL35A	0.151980188	-3.021675168
RPL36	0.157756394	-3.0060638
RPL37	-0.369045847	-4.429853641
RPL37A	-0.142784497	-3.818336478
RPL39	-0.17468209	-3.904546189
RPL4	0.370444445	-2.43123123
RPL41	0.291959604	-2.643352422
RPL5	0.304937075	-2.608278176
RPL6	0.358856591	-2.462549754
RPL7	-0.303516199	-4.252746484
RPL7A	0.202521201	-2.885077835
RPL8	0.290008941	-2.648624484
RPL9	-0.02789642	-3.507828162
RPLP0	-0.714836678	-5.364423454
RPLP1	-0.722811299	-5.385976484
RPLP2	0.002934486	-3.424501389
RPS10	-0.165526796	-3.879802151
RPS11	-0.327114019	-4.316524376
RPS12	-0.996375867	-6.125340181
RPS13	0.041024394	-3.321555692
RPS14	-0.053667237	-3.577479019
RPS15	0.459748549	-2.189868786
RPS15A	0.099822792	-3.162641103
RPS16	-0.100841846	-3.704977962
RPS18	-1.070227344	-6.324938768
RPS19	-0.638198856	-5.157294205
RPS2	-0.659948995	-5.216078365
RPS20	-0.090437308	-3.676857589
RPS21	-0.176664748	-3.909904724

RPS23	-0.05994671	-3.594450568
RPS24	-0.490941625	-4.759301689
RPS25	0.017329264	-3.385596584
RPS26	0.697016628	-1.548603708
RPS27	-0.875204843	-5.797850927
RPS27A	-0.013277107	-3.468316505
RPS28	-0.274810052	-4.175162303
RPS29	-0.207116507	-3.992206776
RPS3	-0.160188939	-3.865375511
RPS3A	-0.690391791	-5.298356192
RPS4X	0.047530492	-3.303971643
RPS5	-0.387931976	-4.480897232
RPS6	-0.194281617	-3.957517884
RPS7	0.53953441	-1.974231324
RPS8	0.081781073	-3.211402505
RPS9	-0.251601967	-4.112437749
RPSA	-0.51552888	-4.82575373
SHMT2	0.595588827	-1.8227329
SLC25A1	0.688213473	-1.572396019
SLIRP	0.582427728	-1.858303438
SNRPD2	0.593938005	-1.827194581
SORT1	0.637260793	-1.710105965
SPATS2L	0.637176577	-1.710333576
STAG1	0.697812249	-1.546453381
SUCLG1	0.700189144	-1.540029341
TAF6L	0.644524581	-1.690474105
TAOK1**	0.490896854	-2.105684178
TBC1D16	0.530782325	-1.997885608
TBCA	0.369125813	-2.4347951
TBCC	0.651668901	-1.671165132
TFPI	0.602394801	-1.804338376
TKT	0.584451965	-1.852832527
TMEM258	-0.120889753	-3.759161495
TNFRSF10D	0.349653114	-2.487424016
TPM4	0.161990985	-2.994618959
TPT1	-0.868682011	-5.780221651
TRIB3	0.358573618	-2.463314546
TROAP	0.588405167	-1.842148197
TSC2	0.629396075	-1.731361959
UBA52	0.418625461	-2.301012268
UCP2	0.525172512	-2.013047265
UQCC2	0.100237135	-3.161521257
UQCRB	0.488362294	-2.112534341
UQCRH	0.607242363	-1.791236857
VPS51	0.670526226	-1.620199389
YBX1	0.096422693	-3.171830559
YBX3	0.29096718	-2.646034649
ZC3H12A	-0.055790523	-3.58321763
ZMYM3	0.550212414	-1.945371854

ZNF358	0.390291796	-2.377589741
ZNF644	0.390368078	-2.377383573

**TAOK1 also has reduced TE in the presence of Silvestrol
